# The genome sequence of the protostome *Daphnia pulex *encodes respective orthologues of a neurotrophin, a Trk and a p75NTR: Evolution of neurotrophin signaling components and related proteins in the bilateria

**DOI:** 10.1186/1471-2148-9-243

**Published:** 2009-10-06

**Authors:** Karen HS Wilson

**Affiliations:** 1 University of Gothenburg, The Sven Lovén Centre for Marine Sciences - Kristineberg, S-450 34 Fiskebäckskil, Sweden

## Abstract

**Background:**

Neurotrophins and their Trk and p75NTR receptors play an important role in the nervous system. To date, neurotrophins, Trk and p75NTR have only been found concomitantly in deuterostomes. In protostomes, homologues to either neurotrophin, Trk or p75NTR are reported but their phylogenetic relationship to deuterostome neurotrophin signaling components is unclear. *Drosophila *has neurotrophin homologues called Spätzles (Spz), some of which were recently renamed neurotrophins, but direct proof that these are deuterostome neurotrophin orthologues is lacking. Trks belong to the receptor tyrosine kinase (RTK) family and among RTKs, Trks and RORs are closest related. Flies lack Trks but have ROR and ROR-related proteins called NRKs playing a neurotrophic role. Mollusks have so far the most similar proteins to Trks (*Lymnaea *Trk and *Aplysia *Trkl) but the exact phylogenetic relationship of mollusk Trks to each other and to vertebrate Trks is unknown. p75NTR belongs to the tumor necrosis factor receptor (TNFR) superfamily. The divergence of the TNFR families in vertebrates has been suggested to parallel the emergence of the adaptive immune system. Only one TNFR representative, the *Drosophila *Wengen, has been found in protostomes. To clarify the evolution of neurotrophin signaling components in bilateria, this work analyzes the genome of the crustacean *Daphnia pulex *as well as new genetic data from protostomes.

**Results:**

The *Daphnia *genome encodes a neurotrophin, p75NTR and Trk orthologue together with Trkl, ROR, and NRK-RTKs. *Drosophila *Spz1, 2, 3, 5, 6 orthologues as well as two new groups of Spz proteins (Spz7 and 8) are also found in the *Daphnia *genome. Searching genbank and the genomes of *Capitella*, *Helobdella *and *Lottia *reveals neurotrophin signaling components in other protostomes.

**Conclusion:**

It appears that a neurotrophin, Trk and p75NTR existed at the protostome/deuterostome split. In protostomes, a "neurotrophin superfamily" includes Spzs and neurotrophins which respectively form two paralogous families. Trks and Trkl proteins also form closely related paralogous families within the protostomian RTKs, whereby Trkls are absent in deuterostomes. The finding of p75NTR in several protostomes suggests that death domain TNFR superfamily proteins appeared early in evolution.

## Background

In mammals, four paralogous neurotrophins play a role in embryonic neural development[1], adult neuroplasticity[2,3] and regeneration[4] of the nervous system: Nerve Growth Factor (NGF), Brain Derived Neurotrophic Factor (BDNF), Neurotrophin 3 (NT3) and Neurotrophin 4/5 (NT4/5). The neurotrophin signaling system is also involved in the immune system[5]. The biological effects of the neurotrophins are mediated by three paralogous Trks (TrkA, TrkB, TrkC) belonging to the Trk family of Receptor Tyrosine Kinases (RTK) as well as by the 75 kDa neurotrophin receptor p75NTR. Each Trk receptor preferably binds to a different neurotrophin, whereby TrkA, TrkB and TrkC can be activated by NGF, BDNF/NT4/5 and NT3 respectively. In contrast, the p75NTR receptor is non selective and has a similar affinity for all neurotrophins. Additional functional complexity results from formation of heterodimeric complexes between p75NTR and TrkA, TrkB or TrkC. These complexes alter the signaling properties of both partners of the complex[6]. Paralogues of p75NTR, known as neurotrophin receptor homologue NRH1 in fish, birds and amphibians, and NRH2 in mammals also form complexes with, and alter signaling by TrkA, TrkB and TrkC. The multiplicity of neurotrophins and Trk receptors in vertebrates apparently arose as a result of two rounds of genome duplications that occurred at the root of vertebrate evolution, the first duplication occurring before the emergence of agnathan fish and the second occurring after the emergence of cartilaginous fish [7-9]. The NRH paralogues of p75NTR may have been generated by a similar mechanism[10]. If the multiple vertebrate paralogues of the components of the signaling system arose by genome duplication events what was the evolutionary origin of the genes that were duplicated by those events?

Until recently, the neurotrophin/Trk/p75NTR signaling system was not found outside the vertebrates. Indeed, no component of this signaling system was present in the genome sequence of two protostomes, the insect *Drosophila melanogaster *and the nematode *Caenorhabditis elegans *[11]. An invertebrate deuterostome, the tunicate *Ciona intestinalis *did not have the signaling system either, which led to hypothesize that neurotrophins, Trks and p75NTR were vertebrate innovations. This hypothesis was challenged, however, when the genome of two additional invertebrate deuterostomes, the cephalochordate *Branchiostoma floridae *(amphioxus)[12,13] and the echinoderm *Strongylocentrotus purpuratus *(purple sea urchin)[14,15] were shown to include the neurotrophin/Trk signaling system in their sequence. Analysis of genomic data from *Strongylocentrotus *as well as EST data from another invertebrate deuterostome, the hemichordate *Saccoglossus kowalevskii *(acorn worm) additionally identified a p75NTR in both of those species[10]. This suggested that the neurotrophin/Trk/p75NTR system arose at least at the root of deuterostomes[8,10]. Clues to an earlier origin of the neurotrophin/Trk/p75NTR system arise from proteins in some protostomes bearing homology to either one of the signaling components of the neurotrophin/Trk/p75NTR system. Nevertheless, none of the neurotrophin/Trk/p75NTR homologues have so far been found concomitantly in a protostomian species. Moreover, when a homologue of a component of the neurotrophin/Trk/p75NTR signaling system has occasionally been described, its phylogenetic relationship to either the neurotrophin or Trk, or p75NTR, has remained uncertain. For example, insects have structural neurotrophin homologues called Spätzle (Spz) proteins[16]. In *Drosophila*, 6 Spz proteins have been identified and are named Spz (or Spz1), Spz2, 3 etc. Spz proteins share a sequence homologous to the characteristic cysteine knot which is known to induce the particular neurotrophin three dimensional fold[17]. Because Spz, Spz2 and Spz5 proteins are organized in the same way as neurotrophins, with a signal peptide and a pro-domain that can be proteolytically removed from the mature protein containing the Cys knot[18], Zhu et al. [19] recently investigated their function in the *Drosophila *nervous system. The researchers demonstrated that *Drosophila *Spz, Spz2 and Spz5 have neurotrophic properties comparable to neurotrophins. In the Zhu paper, *Drosophila *Spz2 and Spz5 were renamed *Drosophila *Neurotrophin 1 (DNT1) and *Drosophila *Neurotrophin 2 (DNT2) respectively, but the exact phylogenetic relationship of Spz, DNT1 and DNT2 to deuterostome neurotrophins could not be resolved. Whether Spzs signal through Trk receptors, which would be expected if they were neurotrophin orthologues is also not known, and the sequence of a Trk receptor orthologue has so far not been found in flies. Spz, the representative of the Spz family is moreover known to signal through the Toll receptor, a receptor involved in the nervous and innate immune systems and in dorso-ventral patterning in the *Drosophila *embryo[20,21].

Trks are modular protein receptors consisting of a conserved extracellular part, a transmembrane domain and a cytoplasmic part. The cytoplasmic part contains a tyrosine kinase domain which is common to many representatives of the RTK family, to which Trks belong. Among RTKs, Trks and RORs share the most similar tyrosine kinase domain[22]. In insects where Trks have not been found, RORs (*Drosophila *ROR and a *Drosophila *ROR-like protein called Neurospecific Receptor Kinase (NRK)) have been shown to have a neurotrophic role[23]. As for protostomian Trk-related sequences, the closest related Trk receptors have been found in mollusks. In the mollusk *Aplysia californica*, a protein called ApTrkl was found to have a tyrosine kinase domain most similar to that of Trk receptors than to any other tyrosine kinase domain[24]. ApTrkl is also expressed, like Trks, in sensory neurons. Nonetheless, the ApTrkl ectodomain and long intracytoplasmic C-terminal extension are totally unrelated to the Trk family of neurotrophic receptors. Accordingly, ApTrkl is not responsive to mammalian neurotrophin stimulation but to serotonin. In another mollusk, the snail *Lymnaea stagnalis*, a Trk related receptor called LTrk, which is specifically expressed in the central nervous system, was found to have most of the characteristics of vertebrate Trk receptors[25,26]. Like ApTrkl, LTrk has a tyrosine kinase domain more related to Trks than to any other RTK. In addition, the LTrk extracellular part displays characteristics of a vertebrate Trk including a leucine rich repeat (LRR) motif flanked by two cysteine clusters. In this extracellular part, however, LTrk lacks two immunoglobulin (Ig) domains of the C2 type, which are a major interface for neurotrophin binding in vertebrates. LTrk has instead an Ig-like domain of the C1 type, which is exclusively common to molecules involved in immune system function, such as immunoglobulins, major histocompatibility complex molecules and T cell receptors. The phylogenetic relationship of *Lymnaea *Trk and *Aplysia *Trkl to each other and to deuterostome Trks is not well understood and neurotrophin like ligands have not been found so far in mollusks.

Finally, no p75NTR has been reported in protostomes. Despite being a neurotrophin receptor, p75NTR does not belong, as do Trk receptors, to the RTK family. Instead p75NTR is a member of the Tumor Necrosis Factor Receptor (TNFR) Superfamily (SF) (TNFRSF) [27]. To date, the only TNFRSF representative in protostomes is the *Drosophila *TNFR Wengen[28]. Wengen has some degree of similarity to p75NTR but it lacks a so-called "death domain" in the intracellular part of the protein. The death domain is present in p75NTR orthologues and in a few vertebrate TNFRs. In this study, the genome of *Daphnia pulex *was investigated for components of the neurotrophin/Trk/p75NTR signaling system. The search was subsequently extended to other protostomes by surveying genbank nucleotide and EST data, as well as the recent genome sequences of *Capitella *sp. I, *Lottia gigantea *and *Helobdella robusta*. The *Daphnia *genome encodes a neurotrophin, a p75NTR and a Trk orthologue together with Trkl, ROR and NRK-RTKs. *Drosophila *Spz1, 2, 3, 5, 6 orthologues as well as two new groups of Spz proteins (Spz7 and Spz8) are also present in the *Daphnia *genome. Neurotrophin signaling components are also found in other protostomes and the evolution of these components in the bilateria is discussed.

## Results and discussion

### Neurotrophin and Spz genes in *Daphnia *and protostomes: Evolution in the bilateria

#### Daphnia Spz genes

##### Daphnia Spz genes

**Figure 1:**
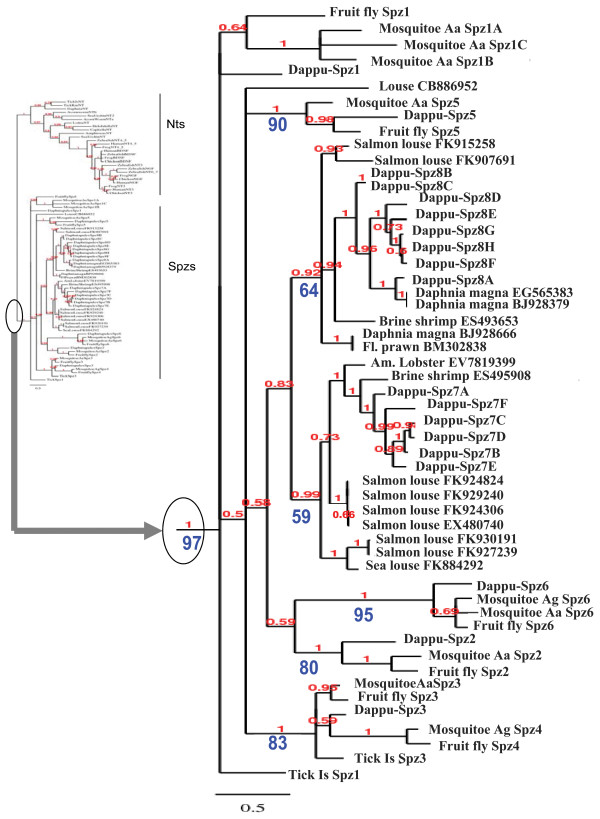
**Phylogenetic tree of Neurotrophin/Spz genes: Spz family**. The full Spz/neurotrophin(Nt) tree is shown on the left side, while on the right side, an enlarged view of the Spz family is depicted. BI phylogenetic tree is shown, but analyses with ML are also represented via bootstrap support values on the tree. BI pp (posterior probability) values figure directly above the branches while ML bootstrap values are directly below the branches. Fruit fly: *Drosophila melanogaster*, Moquitoe Aa: *Aedes aegypti*, Mosquitoe Ag: *Anopheles gambiae*, Dappu: *Daphnia pulex*, Salmon louse: *Lepeophtheirus salmonis*, Sea louse: *Caligus rogercresseyi*, Brine shrimp: *Artemia franciscana*, Fl. prawn: *Penaeus chinensis*, Am. Lobster: *Homarus americanus*, Louse: *Pediculus humanus corporis, TickIs: Ixodes scapularis*, TickRm: *Rhipicephalus microplus*, *Helobdella*: *Hellobdella robusta*, *Capitella*: *Capitella *Sp. I, *Lottia*: *Lottia gigantea*, Human: *Homo sapiens*, Frog: *Xenopus leavis*, Chicken: *Gallus gallus*, Zebrafish: *Danio rerio*, Amphioxus: *Branchiostoma floridae*, Acorn worm: *Saccoglossus kowalevskii*, Sea urchin: *Strongylocentrotus purpuratus*.

**Figure 2:**
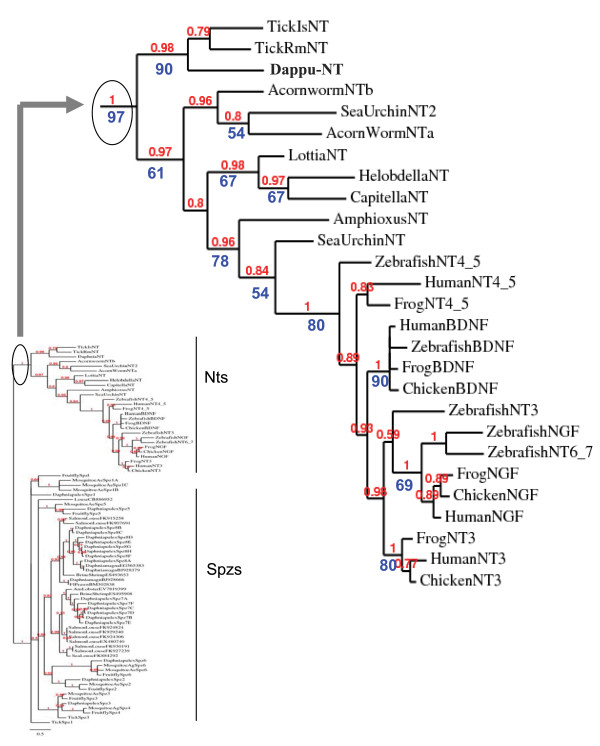
**Phylogenetic analysis of Neurotrophin/Spz genes: Neurotrophin family**. The full Spz/neurotrophin tree is shown on the left side, while on the right side, an enlarged view of the neurotrophin family is depicted. The species names and explanation of numerical values along the branches are described in the legend of Figure 1.

**Figure 3:**
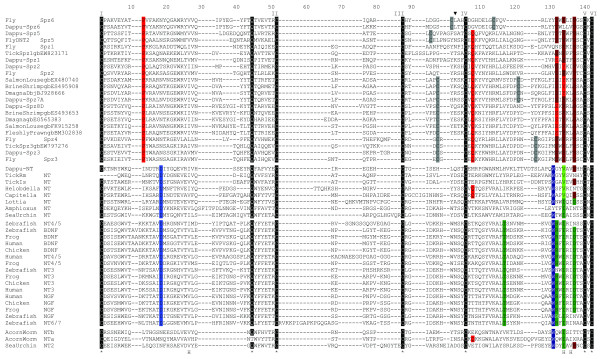
**Alignment of neurotrophin Cys knots to Spz C-106 sequences**. An arrow head (above alignment) indicates where partial Spz6 sequences were removed. I to VI designate conserved "Cys knot" cysteines (white on black). "H" (below alignment) indicates a hydrophobic site. Cys unique to Spz subfamilies are in grey. Highlights indicate frequent characteristic residues of either Spzs (red tones) or neurotrophins (blue-green tones). Canonical invertebrate deuterostome neurotrophins (amphioxus and sea urchin NT) are separated from those belonging to the 2^nd ^sea urchin neurotrophin "NT2" and the two acorn worm ESTs ("NTa" and "NTb"). Dmagna: *Daphnia magna*. Dappu: *Daphnia pulex*.

In insects, such as the fruit fly *Drosophila melanogaster *and the mosquito *Aedes aegypti*, 6 Spz genes, numbered from 1 to 6 have been characterized, whereby the so-called Spz (or Spz1) is viewed as the Spz family representative. Spzs are the closest neurotrophin related proteins in protostomes, and *Drosophila *Spz2 (DNT1) and Spz5 (DNT2) are also termed "neurotrophins" although the orthology to vertebrate neurotrophins has not been strictly demonstrated[19]. Searching for a neurotrophin/Trk/p75NTR system in *Daphnia *included a quest for Spz genes. tBLASTN with, as queries, the amino acid sequences defining the Cys knot (also called C-106) of the 6 insect Spz paralogues yielded *Daphnia *sequence hits to the insect Spzs with low e-value. Further analysis revealed putative *Daphnia pulex *sequences for Spz, Spz2, 3, 5 and 6 respectively and these were named Dappu-Spz1, Dappu-Spz2, Dappu-Spz3, Dappu-Spz5 and Dappu-Spz6. Spz4 was not found in the *Daphnia pulex *genome, but Dappu-Spz3 could be orthologous to both Spz3 and Spz4. Indeed, Spz3s and Spz4s form very close families within Spzs and phylogenetic analyses in this manuscript with both Maximum Likelihood (ML) and Bayesian Inference (BI) show that Spz3s and Spz4s cluster via a node supported by high ML bootstrap and BI posterior probability (pp) values (83% and 1 respectively). These values are similar to those supporting nodes for other respective Spz paralogues (e.g node for Spz5s is supported by ML bootstrap: 90% or BI pp: 1). Moreover, a ML analysis places Dappu-Spz3 with *Drosophila*, *Aedes *and *Ixodes *(tick) Spz3, with 65% bootstrap values, while BI places Dappu-Spz3 closer to *Anopheles gambiae *(mosquito) and *Drosophila *Spz4, but with low pp value (0.59) (Figure 1 and Figure 2). Results for the search of *Daphnia *Spz paralogues are summarized in Table 1 which includes also the scaffold coordinates of the genes, the models predicting the Spz genes and the genbank numbers of supporting ESTs. In addition to known Spz paralogues, searching the *Daphnia *genome with insect Spz2s as queries yielded multiple additional hits with low e-values. The Spz2-like sequences were mostly located on Scaffold 9, but also on other scaffolds (Table 1). For the purpose of this analysis, only Spz2-like sequences with supporting ESTs, were catalogued and further considered. To understand the relationship of the additional Spz2-like sequences to other Spz genes and to the neurotrophins, phylogenetic trees were derived from an alignment of the sequence stretch defining the Cys knot (called C-106 for Spz proteins). The reason for only analyzing the Cys knot was that Spz genes can undergo differential splicing and in some cases, such as for *Drosophila *Spz, alternative splicing results in a high plasticity of the protein N terminal region prior to the Cys knot [29]. Without enough knowledge of the full repertoire of Spz splice variants in *Daphnia *and other species, the use of the Cys knot encoding sequence was devised. This approach was also taken to characterize Spz paralogues in *Aedes aegypti*[30]. Because Spz6 sequences are longer than the other Spz paralogues in the C-106 region, their sequences had to be truncated to allow a correct alignment. An arrow head at amino acid 102 within the alignment in Figure 3 shows the junction where Spz6 sequence fragments were removed. Figure 3 depicts only a portion of the sequences used for the phylogenetic analysis and is for illustration purposes only. The full alignment used for the phylogenetic analysis is appended to this manuscript as supplementary material "see additional file 1". In order to conduct unbiased analyses, phylogenetic trees including and excluding Spz6 were done, and both have the same topology (the tree without Spz6 is not shown as it is redundant). Phylogenetic analyses were conducted with either ML or BI. Figure 1 and/or Figure 2 depict the best BI tree topology where Spz6s are included. Support values for the nodes obtained by both ML and BI figure on the tree, whereby BI pp values figure directly above the branches, while ML bootstrap values are depicted below the branches. In the phylogenetic analysis, Dappu-Spz2, 3 (or 3-4), 5 and 6 cluster with their respective arthropod orthologues with over 80% ML bootstrap values and with constant BI pp values of 1. The orthology of Dappu-Spz1 to other Spz1 (or Spz) orthologues is less supported, but phylogenetic analysis indicates that the Dappu-Spz1 belongs to the Spz family, yet differs from all the other known *Daphnia *Spz paralogues, in particular Spz2s. The pattern formed by the Cysteines within the Cys knot of Dappu-Spz1 suggests that Dappu-Spz1 is a putative Spz (Spz1) orthologue. Indeed, apart from an extra Cys adjacent to the fifth Cys which is responsible for the Cys knot, Spzs (Spz1s) and Spz2s have the particularity of having no additional Cys than those forming the Cys knot, within the Cys knot domain. Since Dappu-Spz1 has the typical Cys arrangement of Spz1s and Spz2s and Dappu-Spz1 can be ruled out as a Spz2 orthologue, Dappu-Spz1 is probably a Spz1 orthologue presenting a high degree of sequence divergence to known Spz1s.

**Table 1:** Catalogue of Spätzle genes found in the *Daphnia pulex *genome.

Gene	Scaffold coordinates	Gene predictions	ESTs
Dappu-Spz	3:3255174-3258541	Dappu-221000	[GenBank:FE349417.1, GenBank:FE349418.1, GenBank:FE355503.1, GenBank:FE355502.1]

Dappu-Spz2	20:559252-563545	Manual prediction	[GenBank:FE361273.1, GenBank:FE325461.1, GenBank:FE325462.1, GenBank:FE413050.1, GenBank:FE413051.1]

Dappu-Spz3	13:218704-222507	Dappu-314968	[GenBank:FE283425.1]

Dappu-Spz5	98:491231-493718	Dappu1-228297	[GenBank:FE334520.1, GenBank:FE334519.1, GenBank:FE389946.1, GenBank:FE389945.1, GenBank:FE380627.1, GenBank:FE380626.1, GenBank:FE376962.1]

Dappu-Spz6	21:959226-964216	Dappu-224270	[GenBank:FE399977.1, GenBank:FE399976.1, GenBank:FE359418.1, GenBank:FE359417.1, GenBank:FE407285.1, GenBank:FE407284.1] *Daphnia magna*: [GenBank:DW724455.1]

Dappu-Spz7A	9:533308-534396	Dappu-313483	[GenBank:FE363720.1, GenBank:FE363719.1, GenBank:FE357035.1]

Dappu-Spz7B	9:513290-514485	Dappu-313475	[GenBank:FE367435.1, GenBank:FE367434.1]

Dappu-Spz7C	9:898918-899886	Dappu-313565	[GenBank:FE380921.1, GenBank:FE380920.1, GenBank:FE309270.1, GenBank:FE309269.1]

Dappu-Spz7D	9:986553-987770 9:1028429-1029516 697:5567-6654	Dappu-313586* Dappu-313593* Dappu-335681*	[GenBank:FE319054.1, GenBank:FE319053.1, GenBank:FE385764.1, GenBank:FE385763.1, GenBank:FE355807.1]

Dappu-Spz7E	6553:13-1284	Dappu-123301	[GenBank:FE352671.1, GenBank:FE299965.1, GenBank:FE299964.1]

Dappu-Spz7F	9:488906-490265	Dappu-309050	*Daphnia magna*: [GenBank:BJ926602.1, GenBank:BJ925927.1, GenBank:BJ925817.1, GenBank:BJ928192.1, GenBank:BJ926968.1, GenBank:FD467132.1, GenBank:DW724571.1]

Dappu-Spz8A	51:574691-575397	Dappu-323037	*Daphnia magna*: [GenBank:EG565383.1, GenBank:BJ928378.1]

Dappu-Spz8B	190166-191014	Dappu-313416	[GenBank:FE328688.1, GenBank:FE328687.1, GenBank:FE322916.1, GenBank:FE322915.1, GenBank:FE416776.1, GenBank:FE416775.1, GenBank:FE420380.1, GenBank:FE420379.1, GenBank:FE353236.1, GenBank:FE353235.1]

Dappu-Spz8C	9:531897-532563	Dappu-313482	[GenBank:FE303670.1, GenBank:FE303669.1, GenBank:FE394292.1, GenBank:FE394291.1, GenBank:FE400682.1, GenBank:FE400681.1]

Dappu-Spz8D	9:530904-531771	Dappu-313481	[GenBank:FE416560.1, GenBank:FE416559.1]

Dappu-Spz8E	46:449347-450049	Dappu-106989	[GenBank:FE373132.1]

Dappu-Spz8F	9:515269-516146	Dappu-313476	[GenBank:FE385611.1, GenBank:FE385610.1, GenBank:FE300572.1, GenBank:FE300571.1, GenBank:FE386869.1, GenBank:FE386868.1]

Dappu-Spz8G	9:1027151-1027987 9:1043641-1044429 9:988211-989048 9:820325-820906 9:803754-804335 697:4289-5125	Dappu-313592* Dappu-313596* Dappu-313587* Dappu-313556* Dappu-313550* Dappu335680*	[GenBank:FE383908.1, GenBank:FE383907.1]

Dappu-Spz8H	51:580305-581023	Dappu-323043	[GenBank:FE347509.1, GenBank:FE347508.1]

Only Spätzle gene predictions with supporting ESTs are reported. An asterisk (*) indicates identical sequences in multiple copies on the same or different scaffolds. All EST numbers are for *Daphnia pulex *unless otherwise stated by *Daphnia magna*.

In the phylogenetic analysis, the Spz2-like sequences that were retrieved by tBLASTN using Spz2 as query form two groups of sequences. Representatives of the first group were named Dappu-Spz7A, 7B etc. while representatives of the second group were named Dappu-Spz8A, 8B etc. to distinguish them from the 6 Spz representatives known to date.

##### Daphnia Spz7s and Spz8s define new Spz paralogues in crustaceans

**Figure 4:**
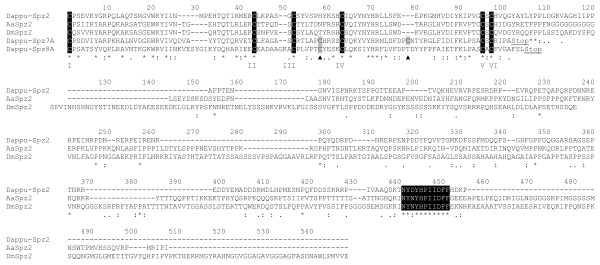
**Alignment of the C-terminal sequence of *Daphnia pulex *Spz7 and 8 to arthropod Spz2s**. Alignment of the Cys knot sequence and C-terminal of one respective representative of *Daphnia pulex *Spz7 (Dappu-Spz7A) and Spz8 (Dappu-Spz8A) to Spz2 sequences of *Daphnia *(Dappu-Spz2), *Aedes *(Aa: *Aedes aegypti*) and *Drosophila *(Dm: *Drosophila melanogaster*). Conserved Cys forming the Cys knot are in white on black background and are numbered below from I to VI. Conserved residues between the sequences are indicated below by an asterisk (*). Arrowheads indicate Cys (also highlighted in grey) characteristically conserved in both Spz7 and Spz8, or only conserved in Spz8. The unique C-terminal extension of Spz2 proteins is present in Dappu-Spz2 as well as a conserved "NY(D/N)YHPIIDFF" sequence motif (in white on black) and these are absent in Spz7 and Spz8 representatives.

To check whether Spz7 and/or Spz8 sequences could be found in other species than *Daphnia*, tBLASTN searches were conducted on genbank with representatives of the Spz7 and Spz8 groups. The search recovered EST sequences from crustaceans only and the latter clustered with either group in the phylogenetic tree. Spz7 sequences cluster together at a node supported by an ML bootstrap value of 59% and a BI pp value of 0.99. Spz8 sequences also respectfully cluster together with an ML bootstrap value of 64% and a BI pp value of 0.92. An alignment of some of the sequences used for the phylogenetic tree is shown in Figure 3. Genbank numbers of crustacean ESTs other than *Daphnia*, falling into the Spz7 or Spz8 groups are indicated in the phylogenetic tree (Figure 1) and in the alignment (Figure 3) next to the species name. The large number of Spz7 and Spz8 (six and eight sequence representatives respectively in *Daphnia*, or more (since only genome predictions with supporting ESTs were considered)) suggests an expansion of these genes in the *Daphnia *genome and perhaps in crustaceans, as both sequence types were also found for example in *Artemia franciscana *(brine shrimp) and *Lepeophtheirus salmonis *(salmon louse). If the genome assembly is correct, some Spz7 genes, such as for example Dappu-Spz7D, are duplicated as identical copies either on different scaffolds or as a tandem on a unique scaffold (Table 1). Tandem gene duplications have been shown for genes under high selective pressure, such as for example, duplication sweeps in the *Culex pipiens *mosquito driven by insecticide treatment[31] or tandem duplications in fish antifreeze proteins as a way to adapt to cooling water temperatures[32]. Because Spz, the representative of the Spz family is involved in innate immunity, the expansion of Spz7 and/or 8 genes could be driven by an immune related ecological response. If this is the case, this could explain why many ESTs encoding such genes are derived from *Daphnia *individuals subjected to stress (Table 2). Spz7 and Spz8 representatives have a conserved Cys within the sequence stretch between Cys III and IV defining the Cys knot (Figure 3). This Cys is also found in Spz3, but not in other Spz paralogues. Spz7 representatives have an additional Cys between Cys IV and V of the Cys knot, and the latter additional Cys is unique to Spz7 proteins when compared to other Spz paralogues. Spz7s and Spz8s have some degree of similarity to Spz2 proteins, since using Spz2s as queries for tBLASTN searches enabled their finding. Some of the "non-*Daphnia*" ESTs encoding Spz7 and Spz8 proteins were described as Spz2-like in genbank. This is somewhat supported by phylogenetic trees obtained by BI. In BI trees, the Spz2 node and the Spz7/Spz8 node are related by a common node. The node is supported by 0.58 pp value in the tree with Spz6 (Figure 1) and by 0.76 pp value in the tree without Spz6s (not shown). Spz7 and Spz8 sequences however differ from Spz2 sequences not only by the presence of additional Cys, but also because they lack a particular C-terminal extension after the Cys knot which is unique to Spz2 proteins. Figure 4 shows an alignment of the Cys knot and C terminal sequence of Spz2 proteins to respective representatives of the Spz7 and Spz8 groups. The unique C-terminal extension of Spz2 proteins with a conserved "NY(D/N)YHPIIDFF" sequence motif (as shown in the alignment by Spz2 orthologues from *Aedes *and *Drosophila*) is present in Dappu-Spz2, but absent in Spz7 and Spz8 proteins.

**Table 2:** Origin of *Daphnia pulex *Spätzle ESTs.

Spz type	Standard conditions	Stress factor
**Dappu-Spz**	[GenBank:FE349417] [GenBank:FE355503]	

**Dappu-Spz3**	[GenBank:FE283425]	

**Dappu-Spz5**		[GenBank:FE389946] Stress: bacterial infection. [GenBank:FE334520] Stress: metal, high [c] (20 ug Cd/L; Stress: 200 ug Zn/L; 1348 ug As/L) [GenBank:FE380627] [GenBank:FE376962] Stress: salt (750 mg/L of sodium chloride).

**Dappu-Spz6**		[GenBank:FE399977] Stress: Metal exposure, low [c] (1 ug Cd/L; 50 ug Zn/L; 500 ug As/L). [GenBank:FE359418] Stress: nickle (200 ug/Lof Ni as nickle chloride). [GenBank:FE407285] Stress: monomethylarsenic III (100 ug/L of As as diiodo-MMAIII).

**Dappu-Spz2**		[GenBank:FE325461] Exposed to Methyl Farnesoate hormone [GenBank:FE361273] Stress: nickle (200 ug/L of Ni as nickle chloride). [GenBank:FE413050] Stress: nanoparticles (Titanium Dioxide).

**Dappu-Spz7A**		[GenBank:FE363720] Stress: copper (5 ug/L of Cu as copper sulphate). [GenBank:FE357035] Stress: nickle (200 ug/Lof Ni as nickle chloride).
**Dappu-Spz7B**		[GenBank:FE367435] Stress: copper (5 ug/Lof Cu as copper sulphate).
**Dappu-Spz7C**		[GenBank:FE380921] Stress: salt (750 mg/L of sodium chloride). [GenBank:FE309270] Stress: arsenic (1348 ug/L of As as sodium arsenite)
**Dappu-Spz7D**	[GenBank:FE355807]	[GenBank:FE319054] Stress: zinc (200 ug/Lof Zn as zinc chloride). [GenBank:FE385764] Stress: nanoparticles (Fullerene).
	[GenBank: FE352671]	[GenBank:FE299965] Stress: hypoxia.

**Dappu-Spz8A**		[GenBank:EG565383] *Daphnia *magna molting cDNA library
**Dappu-Spz8B**	[GenBank:FE353236]	[GenBank:FE328688] Exposed to Methyl Farnesoate hormone. [GenBank:FE322916] Stress: zinc (200 ug/Lof Zn as zinc chloride). [GenBank:FE416776] Stress: Microcystis. [GenBank:FE420380] Stress: calcium starvation (1 mg/Lof Ca).
**Dappu-Spz8C**		[GenBank:FE303670] Stress: hypoxia. [GenBank:FE394292] Stress: mixed metals, high [c] (20 ug Cd/L; 200 ug Zn/L; 1348 ug As/L). [GenBank:FE400682] Stress: mixed metals, low [c] (1 ug Cd/L; 50 ug Zn/L; 500 ug As/L).
**Dappu-Spz8D**		[GenBank:FE416560] Stress: Microcystis.
**Dappu-Spz8E**		[GenBank:FE373132] Stress: acid (pH 6.0).
**Dappu-Spz8F**		[GenBank:FE385611] [GenBank:FE386869] Stress: nanoparticles (Fullerene). [GenBank:FE300572] Stress: hypoxia
**Dappu-Spz8G**		[GenBank:FE383908] Stress: nanoparticles(Fullerene)
**Dappu-Spz8H**	[GenBank:FE347509]	

The first column lists the different Spätzle paralogues. When encoding ESTs were derived from animals kept in standard laboratory conditions, the GenBank EST accession numbers figure in the "Standard conditions" column. When encoding ESTs were derived from animals subjected to a stress, the GenBank EST accession numbers figure in the "Stress factor" column. The particular stress is defined further in the column next to the EST GenBank accession number.

#### Daphnia neurotrophin gene and homologues in the protostomes

##### Predicted Daphnia neurotrophin protein

**Figure 5:**
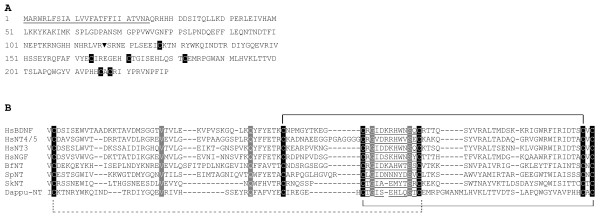
**The sequence of *Daphnia pulex *neurotrophin**. A. Predicted amino acid sequence of Dappu-NT, the *Daphnia pulex *neurotrophin. The signal peptide is underlined and the putative propeptide cleavage site is marked by an arrow head. Conserved Cysteines for the characteristic Cys knot are in white against a black background. B. Alignment of Dappu-NT C-terminus to other neurotrophin C-termini. Identical residues are in white over a grey background. The 5 Cys (white on black background) of the neurotrophin Cys-knot are conserved. Most residues from the vertebrate neurotrophin consensus motif " [GSRE]-C- [KRL]-G- [LIVT]- [DE]-x(3)- [YW]-x-S-x-C" are also conserved (underlined). Hs: *Homo sapiens*, Bf: *Branchiostoma floridae*, Sp: *Strongylocentrotus purpuratus*, Sk: *Saccoglossus kowalevskii*.

Searching *Daphnia pulex *ESTs in the NCBI database by tBLASTN with *Strongylocentrotus *neurotrophins as queries, yielded a partial *Daphnia *EST ([GenBank:FE359769.1], [GenBank:FE359768.1]) with high similarity to neurotrophins. The EST sequence was translated into a peptide sequence and used to tBLASTN search gene predictions on the *Daphnia *genome whereby enabling to recover the full sequence on scaffold 1 (Dappu-94262). The recovered sequence was distinct from all the Spz sequences described above (Figure 5). In vertebrates, neurotrophins are encoded as a precursor containing a signal sequence, a propeptide and the mature neurotrophin. Analysis of the *Daphnia pulex *neurotrophin candidate (Dappu-NT) with the signal sequence prediction software SignalP 3.0 predicts a most likely signal sequence cleavage site between position 25 and 26 (VNA|QR). Analysis of the sequence with the propeptide prediction tool ProP identifies a most probable propeptide cleavage site at position 116 (HNHRLVR|SR). An alignment of Dappu-NT with other deuterostome neurotrophins also shows that the six characteristically spaced Cys residues for disulfide bonding are present (Figure 3 and Figure 5). Dappu-NT does not have the C-terminal extension characterizing Spz2s (including *Drosophila *NT2) (Figure 4).

##### Genomic organization of the putative Daphnia neurotrophin

**Figure 6:**
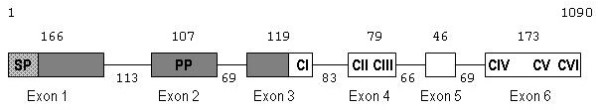
**Predicted Intron-Exon organization of the *Daphnia pulex *neurotrophin gene**. Exons are represented by boxes and the number of nucleotides included within each exon is indicated above the boxes. Introns figure as a line and their length in number of nucleotides is indicated below. The type of sequence encoded by each exon is shown by initials and colour codes. SP (Dotted fill): Signal Peptide; PP (Grey): ProPeptide; Cys knot (white): CI to CVI stand for the six cysteines of the Cysteine knot. CI to CVI figure on the respective exons encoding them.

In vertebrates, the overall genomic organization of neurotrophin genes is similar. Neurotrophin genes have one large major exon of approximately one kb encoding the entire neurotrophin precursor. The precursor major exon is preceded by one or more smaller upstream exons. The Dappu-NT gene is particular since predictions suggest that the precursor is encoded by 6 exons (Figure 6). The exons are not segmented according to the signal peptide, propeptide and mature protein. The signal sequence and part of the propeptide sequence are encoded by the first exon. The rest of the propeptide is also split into two additional exons and so is the sequence encoding the Cys knot which comprises four exons. Although vertebrate neurotrophins are encoded in a more compact genomic structure than Dappu-NT, it is noteworthy to point out that the genome of *Strongylocentrotus *(sea urchin), an invertebrate deuterostome, encodes two neurotrophin types. The first is the recently described Sp-NT (depicted as "sea urchin NT" in Figure 2 and Figure 3). Sp-NT shares the same intron-exon characteristics as vertebrate neurotrophins. The second *Strongylocentrotus *neurotrophin, which I call Sp-NT2 [GenBank: XM_001177318; GenBank XM_001198505] (depicted as "sea urchin NT2" in Figure 2 and Figure 3) has a precursor sequence encoded by several exons, like the *Daphnia *neurotrophin, but with a different intron-exon arrangement (not shown).

##### Other putative protostome neurotrophins

The discovery of a putative neurotrophin gene in *Daphnia pulex *along side Spz genes prompted to ask whether these genes could be found in other protostomes. Search of genbank sequence data with Dappu-NT as query, did not yield orthologous sequences in flies. ESTs of high similarity to the Dappu-NT were however identified in the ticks *Rhipicephalus microplus *and *Ixodes scapularis *(arachnid arthropods). A sequence fragment encoding only part of the Cys knot of the mature neurotrophin was found for *Rhipicephalus *[Genbank: FG579776.1] while longer and respectively overlapping ESTs were retrieved for *Ixodes*. Some of the *Ixodes *sequences were derived from the synganglia, a central nervous system structure [e.g. Genbank: EL516713.1] and the longest EST [Genbank: EL516713.1] encoded a putative signal peptide, prodomain as well as a neurotrophin Cys knot structure in the predicted mature protein. The *Ixodes *neurotrophin sequence was moreover found along side *Ixodes *ESTs encoding different Spz paralogues, such as a putative *Ixodes *Spz1 [Genbank: EW823171.1] and Spz3 [Genbank: EW797276.1]. This suggests that neurotrophins and Spzs are present within several classes of arthropods including crustaceans and arachnids. It is generally accepted that protostomes comprise two groups of animals, the ecdysozoa (comprising nematodes and arthropods such as *Daphnia, Ixodes and Rhipicephalus*) and the lophotrochozoa (comprising flatworms, annelids and mollusks). A number of genomes representing the Lophotrochozoa have recently been made available, such as those of the annelids *Capitella *sp. I, *Helobdella robusta *and the mollusk *Lottia gigantea*. tBLASTN searches on *Helobdella *yielded no Spz genes but a sequence encoding a neurotrophin-like Cys knot fragment on Scaffold 16. The same search method on the *Capitella *genome also resulted in a neurotrophin-like sequence, which was supported by an EST [GenBank: EY519311.1]. The *Capitella *neurotrophin-like sequence has a signal peptide a prodomain and a putative mature Cys knot. A sequence encoding a neurotrophin like Cys knot was also found on Scaffold 27 in *Lottia *but with no corresponding EST. No Spz genes were recovered from lophotrochozoans, but the limited amount of representatives with complete genetic data in this group makes it too early to conclude that Spzs are absent.

##### The Daphnia neurotrophin and its protostome homologues cluster with deuterostome neurotrophins in phylogenetic trees

To verify the relationship of the putative protostome neurotrophin sequences of *Daphnia, Ixodes, Rhipicephalus, Capitella, Helobdella *and *Lottia *to deuterostome neurotrophins, the protostome putative neurotrophin sequences were phylogenetically analyzed with both protostome Spzs (including *Daphnia *and *Ixodes *Spzs described previously) and deuterostome neurotrophins. In the phylogenetic trees obtained by ML and BI, the *Daphnia, Ixodes, Rhipicephalus, Capitella, Helobdella *and *Lottia *neurotrophins cluster with the deuterostome neurotrophins in a "neurotrophin group". Spz, Spz2 (and DNT1), Spz3, Spz4, Spz5 (and DNT2), Spz6, Spz7, and Spz8 paralogues moreover cluster in another large group which is distinct from that formed by the "neurotrophin group". The node at the base of the neurotrophin group is strongly supported both by a ML bootstrap value of 97% and a BI pp value of 1. This implies that neurotrophin and Spz families form paralogous families and in the same time suggests for the first time that protostomes have deuterostome neurotrophin orthologues (Figure 2).

#### Evolution of neurotrophin/Spz genes in the bilateria

##### Spz and neurotrophins form two closely related paralogous families in the protostomes

The phylogenetic analyses presented in this work, show that Spzs and neurotrophins form two paralogous families in the protostomes. Figure 3 shows an alignment of the Cys knot of the major Spz paralogues, to neurotrophins from protostome, invertebrate deuterostome, and vertebrate origin. Three sites, which are numbered in reference to the alignment in Figure 3, distinguish the neurotrophin and Spz families. The first is Pro13 (highlighted in bright red, Figure 3) which is conserved in all of the Spz peptide sequences, but not found in the neurotrophins. The second is an aromatic residue in Spz proteins at position 133 (Phe or Tyr (in dark red, Figure 3)), which is replaced by an Ile or Val in neurotrophins. Finally, Trp 130 (blue) is not found in any Spz proteins, yet it is conserved in all neurotrophins (except for the *Branchiostoma*, "amphioxus NT", that has a Tyr). Despite these three differences, Spzs and neurotrophins share the six Cys forming the Cys knot in neurotrophins as well as Gln44 whose side chain (in neurotrophins), contributes a crucial hydrogen bond to the tertiary structure. Three hydrophobic sites at position 26, 133 and 135 (indicated below the alignment by an H in Figure 3) which locate to the hydrophobic core of the neurotrophins are also common to Spzs and neurotrophins. These similarities and the recent results of Zhu et al. showing that Spzs and neurotrophins have similar functions, argues that the two families are closely related[19]. When the Spz and neurotrophin families diverged, and where they originate in the bilateria, is difficult to pinpoint. Spz genes have not been found in deuterostomes, so if they were already present at the protostome/deuterostome split, they were probably lost in the deuterostome lineage. Alternatively, Spz genes could have evolved from duplications of an ancestral neurotrophin-like gene somewhere in the lineage leading to the protostomes.

##### Evolution of neurotrophins in the bilateria

In the phylogenetic trees, protostome neurotrophins fall into several groups. Ecdysozoa arthropod neurotrophins cluster together (*Rhipicephalus, Ixodes *and *Daphnia*) while another group is formed by the Lophotrochozoa (*Lottia, Capitella *and *Helobdella*) neurotrophins. The invertebrate deuterostome neurotrophin sequences used for the tree include those of a Cephalochordate (*Branchiostoma *(amphioxus in Figure 2)), a Hemichordate (*Saccoglossus *(acorn worm, Figure 2)) and an Echinoderm (*Strongylocentrotus *(sea urchin, Figure 2)). Echinoderms and Hemichordates are closely related and form a clade called Ambulacraria. The two previously described *Strongylocentrotus *neurotrophin paralogues were included in the phylogenetic analysis. Only one neurotrophin has been described for *Saccoglossus *but search of the genbank EST database for the purpose of this study revealed two different ESTs that were arbitrarily called "acorn worm Nta" ([GenBank:FF527248.1] undescribed so far) and "acorn worm Ntb" ([GenBank:FF505731.1] & [GenBank:FF504966.1]) [8,10]. The genome of *Saccoglossus *is still unavailable, so the full repertoire of neurotrophins in this genome cannot yet be investigated. In the tree, ambulacraria neurotrophins form two groups, in accordance with the two *Strongylocentrotus *neurotrophins. The *Strongylocentrotus *neurotrophin Sp-NT2 ("sea urchin NT2" in Figure 2), which does not have the canonical intron-exon organization of vertebrate neurotrophins forms a group with the two *Saccoglossus *neurotrophin ESTs ("acorn worm NTa" and "acorn worm NTb"). The latter group is more closely related to the protostome neurotrophins, as it diverges between the two groups formed by Ecdysozoa and Lophotrochozoa neurotrophins in the tree (Figure 2). The canonical *Strongylocentrotus *neurotrophin Sp-NT ("sea urchin NT" in Figure 2), which shares the same intron-exon organization as vertebrates, clusters closer to *Branchiostoma *("amphioxus NT", Figure 2) and the vertebrates in the tree, consistent with what would be expected for an invertebrate deuterostome neurotrophin. The genome of *Saccoglossus *is not released, so it is not possible to know if this genome encodes an additional canonical neurotrophin type, which would in turn cluster with Sp-NT.

The presence of two *Strongylocentrotus *(Echinoderm) neurotrophins, one more protostome like (Sp-NT2 ("sea urchin NT2", Figure 2)), and the other more vertebrate like (Sp-NT ("sea urchin NT", Figure 2)), may indicate interesting evolutionary transitions occurring at the stem of the deuterostome lineage. The first transitions may be small changes in the protein tertiary and quaternary structure. Sp-NT2 ("sea urchin NT2" in Figure 3) has a Pro at position 136 in the alignment (Figure 3) which is not found in Sp-NT ("sea urchin NT"), yet conserved in some protostome neurotrophins and in all Spz proteins. Pro 136 is at the interface between associated Spz monomers when dimerization occurs and is probably conserved in Spzs for structural reasons. The replacement of Pro136 by other residues in canonical deuterostome neurotrophins may have changed the interface presented by each monomer for dimerization. Sp-NT2 ("sea urchin NT2") also has an additional Cys adjacent to the fifth Cys knot, as does the *Saccoglossus *"acorn worm NTb", which is reminiscent of some Spz paralogues, and which, together with Pro136 allows tight homodimmer formation by covalent disulfide bonding. Finally, Sp-NT2 ("sea urchin NT2") and "acorn worm NTa" and "b" have an Asn at position 18 in the alignment (Figure 3) which is common to many Spz proteins but strictly replaced with an Asp in all canonical neurotrophins (highlighted in blue, Figure 3). Remarkably, Asp18 (18 = alignment Figure 3 numbering, and equivalent to mammalian neurotrophin Asp30) has been shown to be important for tertiary structure stability, and in particular, stability of a beta-hairpin loop formed in neurotrophins by residues 18-22 (18-22 in the alignment, and equivalent to mammalian neurotrophin residues 30-34)[33].

The other transition that may have occurred at the stem of the deuterostome lineage is the occurrence of gene duplications and the adoption by one of the duplicates, of a more compact genomic structure. The precursor sequence of Sp-NT2 is split into several exons, as is the precursor of *Daphnia *neurotrophin and Spzs. The canonical neurotrophin paralogue Sp-NT in *Strongylocentrotus*, however, has a precursor encoded by one exon like vertebrate neurotrophins. The two *Strongylocentrotus *neurotrophins could have arisen from the duplication of an ancestral gene at the base of the deuterostome lineage whereby one or several duplicates retained a more ancestral character leading to Sp-NT2 ("sea urchin NT2") and *Saccoglosssus *"acorn worm NTa" and "b", while another type of duplicate adopted a more compact genomic organization. (The genetic organization of the *Saccoglossus *genes will be interesting to examine in this respect). The gene(s) with ancestral character could have been lost before the divergence of the Cephalochordates on the lineage leading to the vertebrates, leaving a canonical neurotrophin to duplicate via genome duplications along the lineage leading to the vertebrates. It is noteworthy in this regard that neither protostome neurotrophins nor deuterostome invertebrate neurotrophins share a closer relationship to any of the vertebrate neurotrophin paralogues (NGF, BDNF, NT3 and NT4/5). In the phylogenetic tree (Figure 2) the vertebrate genes form a tight cluster. In the alignment, all vertebrate neurotrophins share Thr116 (highlighted in green, Figure 3). Thr116 which is known to form hydrogen bonds across a beta hairpin is not found in any of the invertebrate homologues, thus defining a vertebrate neurotrophin-like characteristic.

### Trk and Trk-related families in the protostomes and evolution in bilateria

#### The Daphnia and protostome Trks

##### Daphnia Trk receptor predicted peptide structure

**Figure 7:**
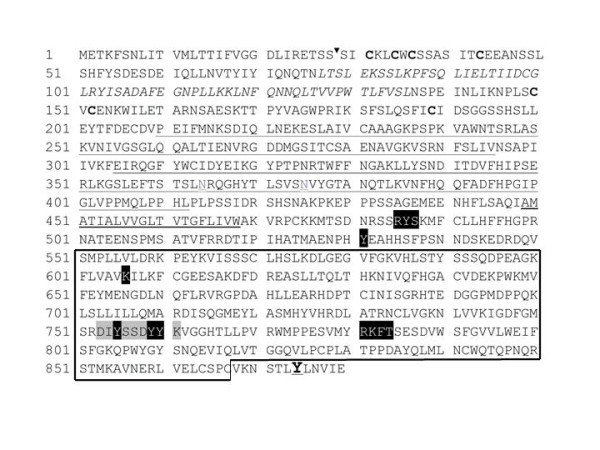
***Daphnia pulex *Trk amino acid sequence**. Amino acid positions are numbered on the left. The putative signal sequence cleavage site is indicated by an arrowhead. Leucine-rich motifs are in italics and flanking cysteine clusters in bold. Both Ig-like domains are underlined; conserved asparagines with structural roles for ligand binding are in grey and double underlined. The transmembrane region is underlined by a thick line. The first phosphorylation site by cAMP/cGMP-dependent kinase proteins, RYS is shown with a black background, as is the tyrosine responsible for Shc recruitment. Within the tyrosine kinase domain (boxed), the lysine responsible for ATP binding and the second phosphorylation site by cAMP/cGMP-dependent kinase proteins, RKFT, are shown by a black background. The autophosphorylation sequence (DIYSSDYYK) is highlighted in grey and the autophosphorylated tyrosines are shown on a black background. The tyrosine responsible for PLC gamma docking is underlined and in larger font.

Searching the *Daphnia *genome with deuterostome Trks and with the protostome *Lymnaea *Trk yielded a unanimous result. A putative Trk receptor for *Daphnia pulex *(Dappu-110423) was found on Scaffold 74 (Dappu V1.1 draft genome assembly/scaffold_74:471874-476291). The predicted *Daphnia *Trk, named Dappu-Trk, has a canonical vertebrate structure at every level (Figure 7). The extracellular region of Dappu-Trk shares a common architecture with the extracytoplasmic domain of vertebrate Trk receptors. It consists of two consecutive Leucine Rich Repeats (LRRs) (Figure 7, italics) flanked by cysteine-rich clusters (Figure 7, bold), followed by two immunoglobulin (Ig)-like domains (Figure 7, underlined). LRRs and cysteine-rich clusters have been reported to participate in ligand binding: TrkB splicing variants lacking LRR motifs are unable to bind any TrkB ligands[34]. The major interface for neurotrophin binding, however, is the second Ig-like domain, where specific residues come into direct contact with the ligand[35]. Both Ig-like domains are of the C2 type in vertebrates, *Branchiostoma *and *Daphnia*, and the most C-terminal pole, which belongs to the second Ig-like domain, contains asparagine residues with structural roles for ligand-receptor interactions (N364 and N375 in Dappu-Trk, Figure 7, grey underlined).

On the cytoplasmic side, Dappu-Trk includes a tyrosine kinase (TK) domain that has all the key residues necessary to carry out its function as a catalytic receptor. Like its vertebrate counterparts, it contains the signature pattern of class II tyrosine kinase receptors DIYSSDYYK (Figure 7, grey background). Within this short amino acid sequence, three tyrosine residues (Y755, Y759 and Y760; Figure 7, black background) constitute the putative auto-phosphorylation activation loop. A presumptive ATP-binding region is located at the N-terminal pole of the TK domain and contains a conserved lysine (K606, Figure 7, black background) responsible for binding ATP. In addition, two potential phosphorylation sites for cAMP/cGMP-dependent kinase proteins lie in positions comparable to those in vertebrates: RYS at the juxtamembrane intracytoplasmic part preceding the TK domain and RKFT following the activation loop within the TK domain (Figure 7, black background). In vertebrates, the former motif is a binding site for SNT, a protein involved in neuronal differentiation and neurite outgrowth pathways[36]. Also present in *Daphnia *Trk is the docking site for Shc, an adaptor protein which in vertebrates activates the Ras-Raf-Erk and PI3kinase-AKT signaling pathways involved in neuronal survival and differentiation events. It is identically placed, preceding the tyrosine kinase domain (Y531, Figure 7, black background). Remarkably, at the furthest C terminus of the protein, outside the TK domain, *Daphnia *Trk has a tyrosine (Y874), which in mammals serves as the docking site for PLC, whose transduction pathway leads to initiation and maintenance of long-term potentiation events[6]. Contrarily to vertebrates, this tyrosine is absent in invertebrate deuterostomes (*Branchiostoma floridae *and *Strongylocentrotus purpuratus*). In this respect, it is noteworthy that out of the two mollusk Trk-like proteins (LTrk and ApTrkl), which share with Trks some of the intracellular region comprising the tyrosine kinase domain, LTrk but not ApTrkl has the vertebrate equivalent tyrosine for PLC gamma activation. This suggests that the tyrosine could either be ancient and lost in specific lineages, or that it occurred independently in several lineages. Two questions are important in this respect, and these are whether LTrk or *Daphnia*Trk can in fact activate the PLC gamma pathway and if the tyrosine residue which has been shown to be absolutely necessary for PLC gamma activation can do this alone outside a consensus vertebrate sequence [P(VIS)YLD(IV)L(GE)] in this region of the protein.

##### Daphnia Trk receptor genomic structure

**Figure 8:**
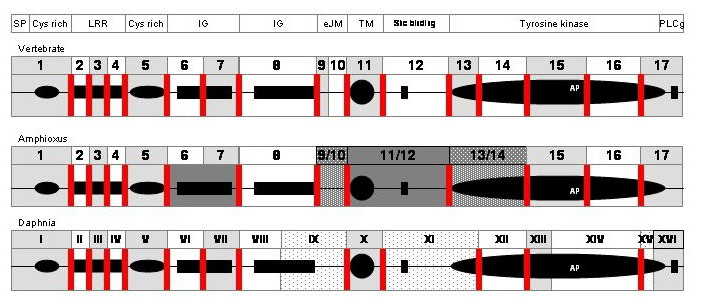
**Genomic organization of the *Daphnia *Trk relative to the vertebrate and amphioxus Trks**. The diagram shows the part of the modular Trk protein encoded by each exon. The upper part of the diagram shows the different domains of the protein while the three diagrams below show the exon organization relative to these domains in vertebrates, amphioxus (Invertebrate deuterostome) and *Daphnia *(Protostome). SP: Signal Peptide; LRR: Leucine rich repeats; Cys rich: Cysteine rich domain; IG: Immunoglobulin domain; eJM: External Juxta-Membrane domain; TM: Transmembrane domain; Shc binding: Shc binding site; Tyrosine kinase: Tyrosine kinase domain; PLCg: Phospholipase C-γ docking site. Intron exon junctions are represented by vertical lines across the rectangle that represents each protein-coding sequence. Conserved intron-exon junctions between any invertebrate species and vertebrates are in red.

The theoretically predicted *Daphnia *Trk (or Dappu-Trk) transcriptional unit consists of 16 exons which are depicted in Figure 8. For clarity, *Daphnia *exons are numbered in Roman characters, while vertebrate and *Branchiostoma *(amphioxus Trk in Figure 8) exons are numbered in Arabic numbers. The first five exons in *Daphnia *Trk exhibit the same domain arrangement as vertebrate and amphioxus Trk receptors. The signal peptide and the N-terminal cysteine-rich cluster are coded by the first exon, while the LRR are spread among exons II, III and IV, leaving the C-terminal cysteine-rich cluster in exon V. Like vertebrate Trk proteins, and in contrast to amphioxus Trk, the first Ig-like domain of *Daphnia *Trk is encoded by two exons (exons VI and VII).

The second IgC2 domain in *Daphnia *Trk is also encoded by 2 exons (*Daphnia *Exon VIII and IX) unlike vertebrate and amphioxus Trks. Exon IX also spans the extra-juxta-membrane sequence of the receptor and covers what is part of vertebrate exon 8, 9 and 10. The extracellular juxta-membrane region in amphioxus and vertebrate Trks is encoded by distinct exons to those bearing the second Ig. In vertebrates, exons 9 and 10 encode the extracellular juxta-membrane region, while in amphioxus Trk a single compact exon encompasses vertebrate exons 9 and 10. Interestingly, human Trk exon 9 encodes a short amino acid sequence alternatively spliced in several of the Trk isoforms described in vertebrate species. The presence or absence of this short amino acid sequence is tissue specific and affects ligand preference. Human and rat TrkAI isoforms, which lack six amino acids in this region (encoded by exon 9), are expressed in non-neuronal tissues and bind only NGF, while TrkA-II isoforms, which contain the six amino acid insertion, are localized in neuronal tissues and can interact with both NGF and NT3 [37]. The absence of this mini-exon in amphioxus and *Daphnia *Trks suggests that Trks may have acquired more ligand flexibility in the lineage leading to the vertebrates. The transmembrane region of the *Daphnia *Trk protein and the first phosphorylation site for cAMP/cGMP-dependent kinase proteins, RYS, are encoded by *Daphnia *exon X. *Daphnia *exon X aligns to vertebrate exon 11, with similar intron-exon boundaries. Vertebrate exons 12 and 13 align to *Daphnia *exon XI which is more compact and spans not only the tyrosine responsible for Shc recruitment Y533 (covered by exon 12 in vertebrates) but also part of the tyrosine kinase domain (encoded by vertebrate exon 13). The breakpoint between exon XI and XII in *Daphnia *corresponds to the breakpoint between human Trk exon 13 and 14 centrally located in the ATP binding residue K606. The tyrosine kinase domain and the intracytoplasmic C-terminus are distributed over five exons (*Daphnia *exons XII, XIII, XIV, XV and XVI). *Daphnia *exon XII corresponds to vertebrate exon 14, while *Daphnia *exon XIII does not span as much peptide sequence as vertebrate exon 15 and does not include the autophosphorylation sequence. This sequence is present instead in the next *Daphnia *exon XIV. The breakpoint between exon XIV and XV corresponds roughly to the breakpoint between vertebrate exon 16 and 17. In vertebrates, the C-terminal of the tyrosine kinase domain and the tyrosine responsible for PLC gamma docking are encoded by one exon (exon17). In *Daphnia*, these entities are encoded by two separate exons (exon XV and XVI). The intron-exon organization of *Daphnia *Trk, with more exons than amphioxus Trk, and several intron-exon boundaries that are conserved with vertebrate Trks, reopens the question on how the ancestral organization of the Trk gene looked like.

##### Daphnia Trk is a deuterostome Trk orthologue

**Figure 9:**
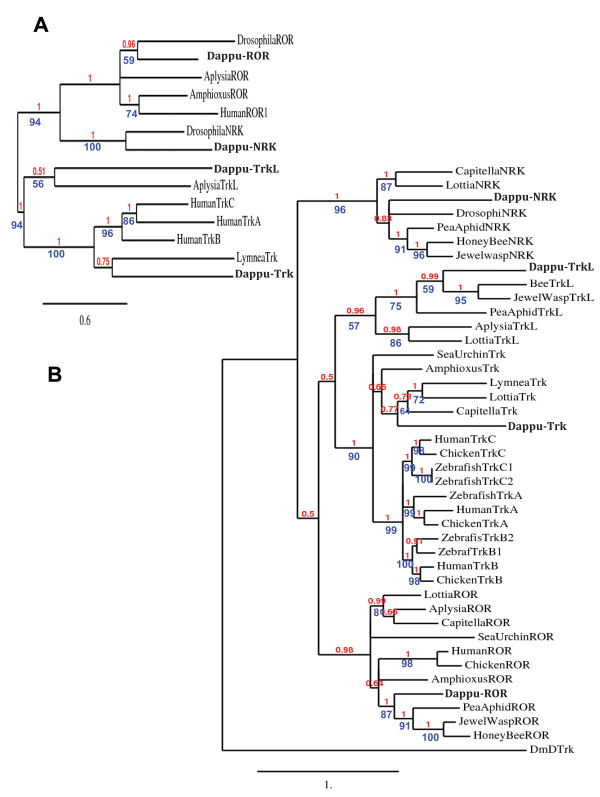
**Phylogenetic analysis of RTKs**. Trks, Trkls, NRKs and RORs are analyzed together with *Drosophila *DTrk (DmDTrk) as an outgroup. A: Phylogenetic tree based on full length sequences: A BI derived tree topology is shown as well as support values for the nodes computed by BI (pp: posterior probability directly above the branches) and ML (ML bootstrap values directly below the branches). B: Phylogenetic tree based on sequences encoding the tyrosine kinase domains only. The topology and support values are depicted in the same way as in A. Human (*Homo sapiens*), Chicken (*Gallus gallus*), Zebrafish (*Danio rerio*), Amphioxus (*Branchiostoma floridae*), Sea urchin (*Strongylocentrotus purpuratus*), *Lottia *(*Lottia gigantean*), *Aplysia *(*Aplysia californica*), *Dappu-*(*Daphnia pulex*), *Capitella *(*Capitella *Sp. I), Honey Bee (*Apis mellifera*), Pea Aphid (*Acyrthosiphon pisum*), Jewel wasp (*Nasonia vitripennis*), *Drosophila *(*Drosophila melanogaster*).

To establish the relationship of Dappu-Trk to deuterostome Trks, phylogenetic trees were generated with ML and BI. The trees were derived from full length sequences of Dappu-Trk, *Lymnaea *Trk and *Homo sapiens *Trks, as well as from full length sequences of closely related RTKs like RORs, NRKs and *Aplysia *Trkl [38] "see additional file 2". The phylogenetic analysis (Figure 9A) results in a Trk group including Dappu-Trk, *Lymnaea *Trk and the *Homo sapiens *Trks (human TrkA, B and C). The node for the Trk group is supported by high ML bootstrap (100%) and BI pp values (1) and distinguishes Trks (including Dappu-Trk) from other RTKs. It is noteworthy that the *Lymnaea *Trk clusters within the Trk group, despite differences in its extracellular part.

##### Search for other protostomian Trks

The presence of a putative Trk orthologue in *Daphnia *suggested that Trks could perhaps be found in other protostomes. tBLASTN of genbank, with the *Daphnia *sequence did not yield any Trks in flies, but a search on the *Capitella *and *Lottia *genomes gave promising results. The *Capitella *and *Lottia *genes retrieved from the respective genome sequences are incomplete and may have errors particularly in the first half of the sequences. This could be due to missing sequence data and/or assembly problems. For both *Capitella *and *Lottia*, the sequence encoding the signal peptide and the first Cys cluster expected for Trks is lacking. A rather complete LRR is present at the N terminal of the *Capitella *amino acid sequence, while the *Lottia *sequence begins at what could be the end of the LRR. Both *Lottia *and *Capitella *sequences present a Cys Cluster after the LRR followed by a sequence encoding an IgC2 domain, similar to deuterostomes and *Daphnia *in this respect, but different from *Lymnaea *Trk (which is a Mollusk representative like *Lottia*). Indeed, *Lymnaea *Trk does not have an immunoglobulin domain in this region. What could be remnants of a second IgC2 domain (or an incorrect IgC2 sequence due to errors) follows the first IgC2 in *Lottia *and *Capitella*. Deletions occur, however, relative to the *Daphnia *sequence in this region, particularly in *Capitella*. The deletions are most important in the region after the two Asn residues which are known to interact with neurotrophin ligands in vertebrates. *Lymnaea *has an immuglobulin of the C1 type in this equivalent region. Presently, it is difficult to judge whether the deletions are "true" in evolutionary terms, or if they represent sequence errors, because in *Capitella*, the transmembrane domain which should follow the second Ig is absent and there are deletions relative to *Daphnia *in what should be the extracellular and intracellular regions immediately surrounding the transmembrane domain. The *Lottia *Trk sequence has a recognizable transmembrane domain. Despite possible errors in the first halves of the *Lottia *and *Capitella *sequences, the sequences encoding the intracellular part of the receptor prior to and within the tyrosine kinase domains meet the expectation for Trk sequences. Prior to the tyrosine kinase domain, the the NPxY motif is present in both *Lottia *and *Capitella *sequences. Also, the tyrosine kinase domains of both *Lottia *and *Capitella *have the typical autophosphorylation sequence [D(V/I)(S/T)(S/T)DYYR]. At the furthest C-terminus of the proteins, outside the TK domain, *Lottia *and *Capitella *Trks have a tyrosine equivalent to the mammalian Trk PLC docking site.

##### Phylogenetic analysis of Trk receptors in the bilateria

The sequence of the mollusk *Lottia *Trk appeared to be more similar to *Daphnia *and deuterostome Trks in the encoded extracellular domain, than to *Lymnaea *Trk, another molluskan Trk. The *Lottia *genome was therefore searched with the *Lymnaea *Trk as query, to check for the presence or absence of a *Lymnaea *Trk type (with an IgC1 in the extracellular domain) in *Lottia*. The *Lymnaea *Trk type could not be found in *Lottia*. Because of some uncertainties remaining regarding the first respective halves (encoding N terminal) of the *Capitella *and *Lottia *Trk sequences, phylogenetic trees were derived from tyrosine kinase domains only (Figure 9B). The phylogenetic analysis also included closely related RTK tyrosine kinase sequences (RORs, NRKs and Trkl) as well as the tyrosine kinase domains of *Daphnia *and deuterostome (*Branchiostoma *(amphioxus in Figure 9B), *Strongylocentrotus *(sea urchin), *Homo *(human), *Gallus *(chicken) and *Danio *(zebrafish)) Trks "see additional file 3" (Figure 9B)[12,39,40]. The orthology of *Daphnia*, *Capitella*, *Lymnaea *and *Lottia *tyrosine kinase domain to deuterostome Trks is supported by BI and ML. The node supporting the Trk family within the RTKs has a ML bootstrap value of 90% and a BI pp value of 1. Within the Trk tree, vertebrate Trks cluster together. Invertebrate Trks do not bear any closer relationship to any vertebrate Trk A, B or C paralogue. Despite the fact that *Lottia *and *Lymnaea *have different extracellular domains, the molluskan Trk tyrosine kinases cluster together with high support and are closely related to the *Capitella *and *Daphnia *Trks (Figure 9B). The finding of Trks in some protostomes but not in *Drosophila *and *Caenorhabditis *suggests that Trks are ancient but have been lost in some lineages. The finding of two IgC2 domains on the extracellular side of *Daphnia *Trk (ecdyzosoan) and at least one IgC2 domain (and possibly traces of another) in *Capitella *and *Lottia *(lophotrochozoa) suggests that a Trk bearing all the characteristics of deuterostome Trks was probably present before the protostome/deuterostome split.

#### Daphnia Trkl receptor and a Trkl-related lineage in the protostomes

Before the discovery of the Dappu-Trk, the closest protostomian related Trks were the *Lymnaea *Trk (which now appears to be a Trk orthologue) and the *Aplysia *Trkl. The search of the *Daphnia *genome with vertebrate Trks and *Lymnaea *Trk yielded the previously described Dappu-Trk gene. Investigating the *Daphnia *genome for *Aplysia *Trkl sequences however, uncovered a Trkl homologue in *Daphnia *(Dappu-235827). When *Aplysia *Trkl was first reported, it was described as having the tyrosine kinase domain most similar to vertebrate Trks but less so than *Lymnaea *Trk. The precise phylogenetic relationship between *Lymnaea *Trk and *Aplysia *Trkl is so far not known, but the existence of a putative Trkl protein in *Daphnia *led to hypothesize that Trks and Trkls could form two paralogous families in the protostomes. Despite a strong similarity to Trks in the intracellular region, Trkl bears little similarity to Trks in the extracellular domain of the receptor. In *Aplysia *Trkl, the initiating Met is followed by a relatively small ectodomain including the fragment of an EGF domain. The ectodomain is followed by a single transmembrane region, a tyrosine kinase domain and a long C-terminal extension.

The *Aplysia *Trkl also differs from Trks in that it does not have a precursor sequence with a signal peptide.

**Figure 10:**
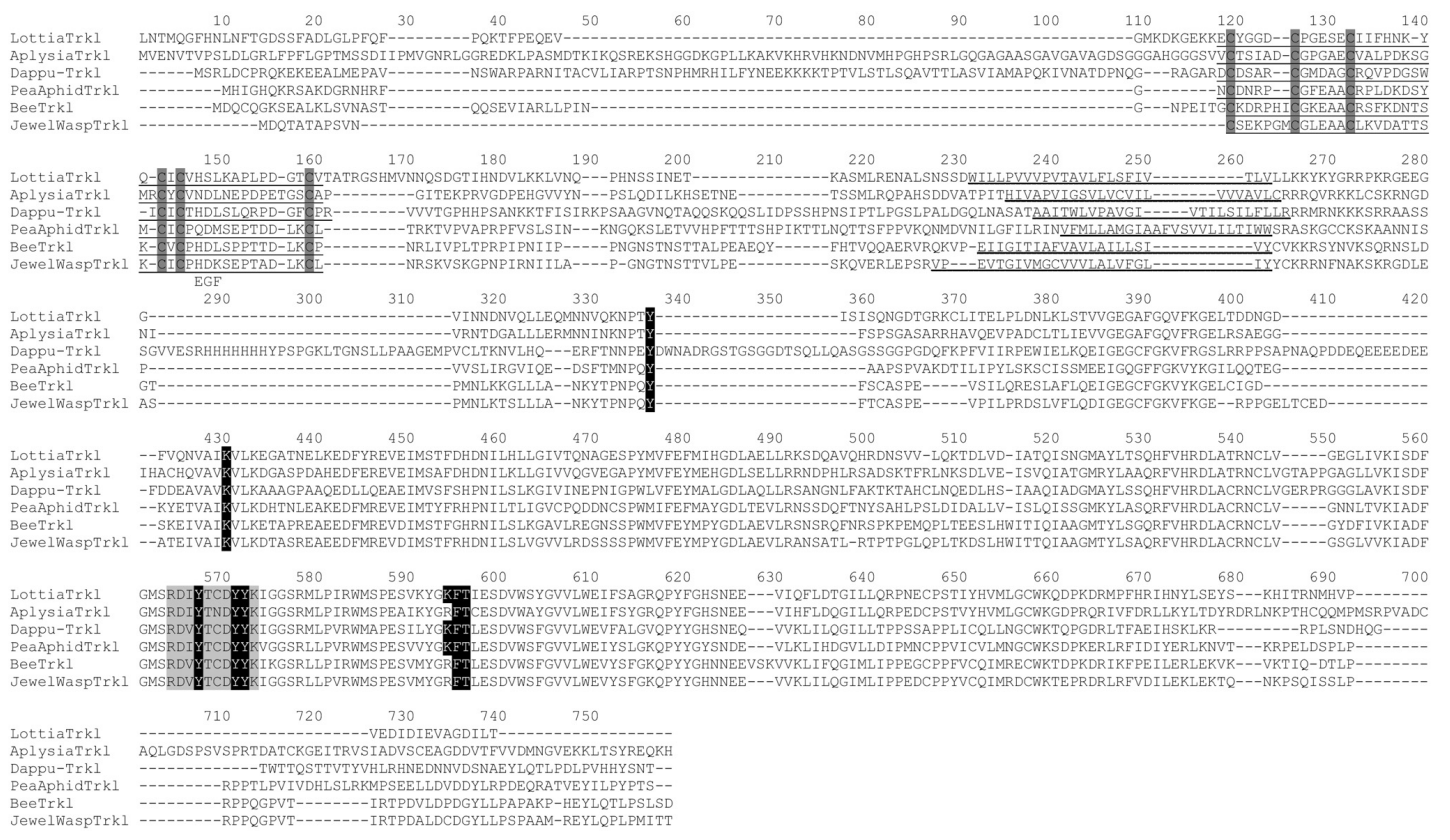
**Alignment of Trkls**. Dappu- (*Daphnia pulex*), Bee (*Apis mellifera*), Pea Aphid (*Acyrthosiphon pisum*), Jewel wasp (*Nasonia vitripennis*), *Lottia *(*Lottia gigantea*) and *Aplysia *(*Aplysia californica*) trkl sequences are aligned. The EGF domain is underlined and conserved Cys are highlighted. The transmembrane domain is underlined. The Tyr in the NPxY motif prior to the tyrosine kinase domain is highlighted black on white. Important functional residues within the tyrosine kinase domain such as the phosphorylation site YXXDYY are also highlighted.

Analysis of the putative *Daphnia *Trkl (Dappu-Trkl), revealed the absence of a signal peptide, as in *Aplysia *Trkl. The extracellular domain of Dappu-Trkl has an EGF domain which is followed by a transmembrane domain and a tyrosine kinase domain on the intracellular side (Figure 10). The Tyrosine kinase domain includes the NPxY signaling motif found in *Aplysia *Trkl and Trks. Like *Aplysia *Trkl, Dappu-Trkl has a C terminal extension after the tyrosine kinase domain, but this extension is shorter. Phylogenetic trees derived from BI and ML analysis of the full length sequences (Figure 9A) group *Daphnia *and *Aplysia *Trkl together. The *Daphnia*/*Aplysia *Trkl node is separate from a highly supported Trk node (ML bootstrap: 100% and BI pp: 1) regrouping *Homo *(human in Figure 9A), *Lymnaea *and *Daphnia *Trks. In the trees with the full length sequences, the Trkl and Trk family nodes are nonetheless grouped by a common node (ML bootstrap: 94% and BI pp: 1) suggesting that Trkls and Trks are more closely related to each other than to ROR and NRK RTKs. Search for Trkl RTKs was performed on the *Lottia *and *Capitella *genomes. tBLASTN with Trkls as queries, gave hits for a fragment encoding a *Capitella *tyrosine kinase domain with low e-value, but the sequence was too short to include the characteristic EGF domain so it was not further analyzed. A full sequence was found on *Lottia *Scaffold 225, and this sequence is partially supported by an EST [Genbank: FC759201.1]. tBLASTN searches against NCBI ESTs helped recover a number of Trkl sequences from insect arthropods (*Apis mellifera *(honey bee): [Genbank: XM_001121533.1], *Nasonia vitripennis *(jewel wasp): [Genbank: XM_001602328], *Acyrthosiphon pisum *(pea aphid): [Genbank: XM_001944304.1]), but not from flies. All the insect sequences encode a number of conserved Cys for an EGF domain on the extracellular side, a transmembrane domain and a tyrosine kinase domain on the intracellular side. The tyrosine kinase domains of Trkls, although very similar to Trks, have subtle differences, which separate them from Trks, RORs and NRKs in ML or BI trees derived from the tyrosine kinase domain sequences only (Figure 9B). The finding of a Trkl lineage helps to resolve the so far unclear relationship between *Lymnaea *Trk and *Aplysia *Trkl, whereby these two proteins represent two closely related families in the protostomes. It was surprising to find Trkl in insects because Trkls have not been found in flies. Because *Daphnia*, a crustacean arthropod, has both Trkl and Trk, Trks and Trkls are likely to have disappeared along some arthropod lineages.

#### The Daphnia ROR and NRK receptors and their protostome homologues

##### Daphnia and protostome RORs

**Figure 11:**
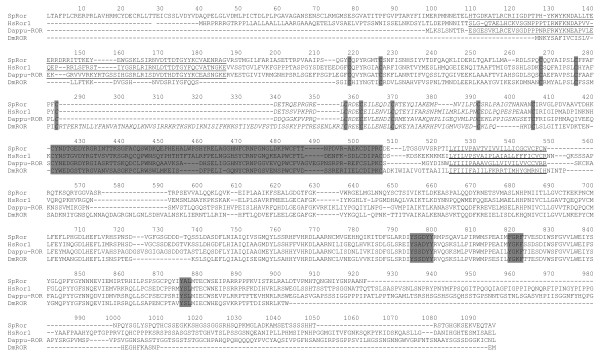
**Alignment of *Daphnia *ROR to other ROR sequences**. Alignment of *Daphnia pulex *ROR (Dappu-ROR) with human (*Homo sapiens *(Hs)) ROR1, fly (*Drosophila melanogaster *(Dm)) ROR and sea urchin (*Strongylocentrotus purpuratus *(Sp)) ROR. The SpROR N-terminal does not figure in the alignment for space purposes, it contains an N-terminal extension with an additional Immunoglobulin domain (not shown) that does not align to its counterparts. In the alignment, the immunoglobulin domain on the extracellular side is underlined, while the frizzled Cystein Rich Domain (CRD) is in italics with conserved cysteines highlighted in grey. In the extracytoplasmic part, the kringle domain is highlighted in grey. The transmembrane domain is underlined. In the cytoplasmic tyrosine kinase domain of the protein, the YXXDYY that corresponds to the site of phosphorylation within the TrkB activation domain is highlighted in grey. Potential SH2 binding sites are also highlighted.

**Figure 12:**
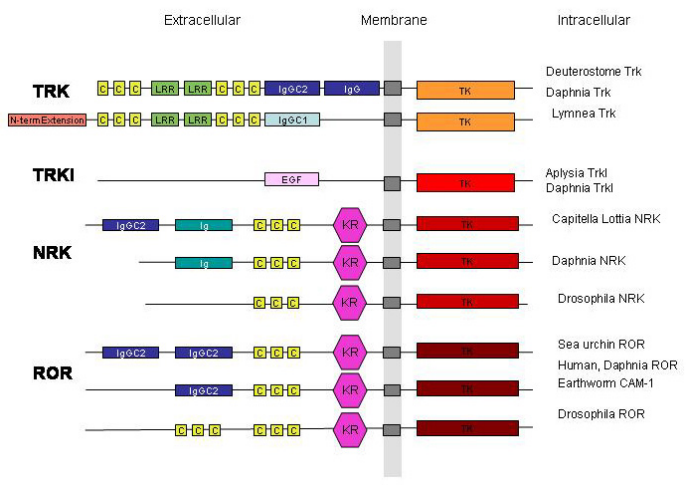
**Schematic representation of Trk-related RTKs**. Organization of Trk, Trkl, NRK and ROR protein domains in various protostome and deuterostome representatives: The intracellular tyrosine kinases (TK) are depicted in different shades of red depending on their affiliation to the Trk, Trkl, NRK or ROR families. The transmembrane domains are in grey. The immunoglobulin (Ig) domains are in different shades of blue depending on their type (Ig, IgC1 or IgGC2), the Kringle domains are in dark pink (KR), the EGF domain is in light pink, the Cys Rich Domains are in yellow (C), and leucine rich repeats are in green (LRR).

All previous analyses in this work were mostly aimed at showing that Trk orthologues are present in some protostome representatives. For this, RORs and NRKs were used as references in the phylogenetic analyses. Indeed, a part from Trkl, these RTKs have tyrosine kinase sequences closest related to Trks. In *Drosophila*, Trk and Trkl receptors have not been found, but ROR plays a neurotrophic role. Hanks classification of the membrane spanning protein tyrosine kinases regroups Trk and ROR receptors in a single class, since among all the different protein tyrosine kinases their respective tyrosine kinases are most similar to each other than to any other protein tyrosine kinase[22]. To define the relationship of *Daphnia *Trk and Trkls relative to ROR proteins, a putative ROR protein was sought in the *Daphnia *genome. One gene prediction on scaffold 128 (Dappu-203169) appeared as a good ROR candidate and was named Dappu-ROR (Figure 11). RORs are characterized by an N terminal Cys rich region followed by a kringle domain in their extracellular part. Aside from this, the extracellular part is quite variable depending on the species. For example, *Drosophila *ROR has an additional Cys rich region prior the conserved Cys rich region and kringle domain, while *Strongylocentrotus *and *Homo *RORs (depicted as sea urchin and human ROR in Figure 12) have one to two N terminal IgC2s prior to the conserved Cys rich region and Kringle domain (Figure 12). In the extracellular part of the *Daphnia *ROR, one N-terminal IgGC2 precedes the canonical Cys rich region and Kringle domain, similar to human ROR. The protein has a transmembrane domain followed on the C terminal side by a tyrosine kinase class II domain. The latter tyrosine kinase domain contains the characteristic YXXDYY phosphorylation site as well as potential SH2 binding sites (Figure 11). The full length Dappu-ROR was aligned to full length sequences of other RORs as well as Trks, Trkls and NRKs. Phylogenetic trees were derived from the alignment by BI and ML. In the trees with BI analysis, RORs form a distinct protein family (BI pp 1) (Figure 9A) but the unity of this family is not as well supported by ML, which nevertheless places individual ROR representatives outside the other families formed by Trk, Trkl, and NRK (not shown). RORs were also sought in protostomes that had been previously shown to have a Trk, or Trkl. For *Capitella *and *Lottia*, a genome search against gene models retrieved two predicted ROR gene candidates (*Lottia*: jgi|Lotgi1|128683|e_gw1.62.3.1; *Capitella*: jgi|Capca1|52072|gw1.14.82.1). tBLASTN searches on the NCBI database also uncovered putative RORs for arthropods: *Acyrthosiphon *(pea aphid) [Genbank: XM_001948569], *Apis *(honey bee) [GenBank: XM_397058] and *Nasonia *(jewel wasp) ROR [Genbank: XM_001601258]. The *Capitella*, *Lottia*, *Acyrthosiphon *and *Apis *ROR sequences encode the same extracellular arrangement as *Daphnia *and human RORs (ie One IgC2 followed by a Kringle domain). The *Nasonia *ROR has no Ig domains in the extracellular part, but just a Kringle domain as in *Drosophila *ROR. In trees compiled with the sequence portions encoding the tyrosine kinase domains only, BI strongly supports RORs forming a single family of proteins paralogous to Trks, Trkls and NRKs (Figure 9B).

##### Daphnia and protostome NRKs

NRKs are so far not very well described outside flies, and have not been reported in deuterostomes. *Drosophila *NRK (Dnrk) has an extracellular organization reminiscent of RORs, yet a tyrosine kinase domain more closely related to Trks than ROR[38]. Dnrk is expressed specifically in the developing nervous system during embryogenesis. *Daphnia *also has an NRK called YasNRK which is described elsewhere (Yasuhiro Shiga)[41]. Like RORs, NRKs were used as references, to investigate protostome Trks. Following the same approach as with RORs, NRKs were sought for the protostomes that were shown to have a Trk or Trkl protein sequence. NRKs have a cys rich region followed by a Kringle domain in their extracellular part. After the transmembrane domain, the intracellular tyrosine kinase domain of the *Drosophila *NRK has the particularity of having two tandemly repeated putative ATP-binding sites which are reminiscent of Dtrk, another *Drosophila *RTK. A number of putative NRK sequences were retrieved from the NCBI nucleotide database, including those of *Apis *(honey bee) [Genbank: XM_391863], *Nasonia *(jewel wasp) [Genbank: XM_001601666] and *Acyrthosiphon *(pea aphid) [Genbank: XM_001942924.1]. A putative *Lottia *NRK was found in the *Lottia *genome assembly (Scaffold 53), while a putative *Capitella *NRK (jgi|Capca1|161686|estExt_Genewise1.C_10213) was retrieved from searching gene predictions in the *Capitella *genome. Insect (*Apis*, *Acyrthosiphon*, *Nasonia*) sequences encoded an extracellular region with the same organization as the insect *Drosophila*. *Daphnia *has an immunoglobulin preceding the kringle domain on the N terminal. In *Lottia *and *Capitella*, the N terminal consists of an IgC2, followed by an Ig and the consensus Kringle domain (Figure 12). Instead of having two ATP consensus sites in the tyrosine kinase domain, all the newly found sequences (including *Daphnia*) had only the second ATP site equivalent, and this site had a very conserved sequence "GQGAFG". In phylogenetic trees (Figure 9) derived from either full length or tyrosine kinase sequences, NRKs form a family of proteins. The node supporting this family has an ML bootstrap value of 100% and a BI pp of 1 for full length sequence derived trees, and an ML bootstrap value of 96% and a BI pp of 1 for tyrosine kinase sequence derived trees. No NRKs could be found so far in deuterostomes.

#### Evolution of Trk and Trk-related RTKs in the bilateria

The finding of Trk orthologues in the protostomes suggests that a Trk protein was already present at the protostome/deuterostome split. The absence of a Trk in *Drosophila *has been attributed to a lesser need for plasticity of the insect nervous system[42]. This may not be the case for all arthropods as reflected by the *Daphnia *Trk. The additional finding of a Trkl family of protein in the protostomes, which is very closely related to Trks, suggest that protostomes have a complex array of Trk related proteins, including NRKs, which are not found in deuterostomes.

### P75NTR genes in the protostomes and evolution in the bilateria

#### p75NTR in Daphnia and other protostomes

**Figure 13:**
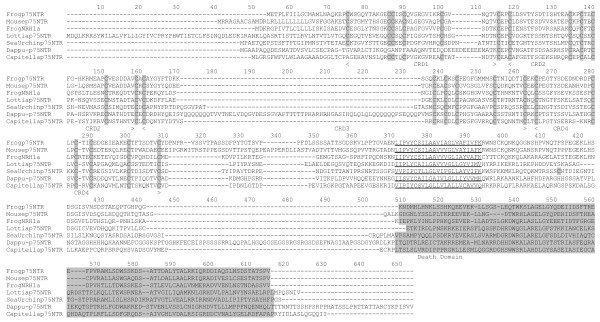
**Alignment of *Daphnia pulex *p75NTR to other p75NTRs and frog NRH1**. Dappu- (*Daphnia pulex*), Mouse (*Mus musculus*), Frog (*Xenopus leavis*), Sea Urchin (*Strongylocentrotus purpuratus*), *Lottia *(*Lottia gigantea*) and *Capitella *(*Capitella *Sp. I). Identical residues are marked by an asterisk. Conserved Cysteines in the "Cys Rich Domain" (CRD) that defines TNFRSF members are highlighted in grey. The transmembrane domain is underlined. The death domain is highlighted in grey.

The finding of a neurotrophin and Trk receptor in *Daphnia *prompted to ask whether a p75NTR could be present in this species. Despite being a neurotrophin receptor, p75NTR does not belong, as do Trk receptors, to the RTKs. Instead p75NTR is a member of the TNFRSF. TNFRSF members have one to four repeats of cysteine-rich domains (called CRD) in their extracellular domain. Except for the Cys residues, the CRDs show very low sequence conservation between the various TNFRSF. The cytoplasmic regions of the receptors show considerably more diversity in sequence and size than the extracellular regions. There are no common intracellular motifs found in all members of the TNFR superfamily except for some domains such as the TRAF2-binding domain, which is required for both NF-κB activation and JNK activation, or a domain of ~ 80 amino acids called the "death domain" for caspase activation. To date, no TNFR proteins (including p75NTR) have been found in protostomes, except for the *Drosophila *TNFR Wengen[28]. Wengen lacks a so called "death-domain" in its protein intracellular part, which is present in p75NTR orthologues and in less than one third of vertebrate TNFRs. Because the majority of vertebrate TNFRs do not have a death domain and because Wengen has so far been the only known protostome TNFSFR, it has been suggested that the ancestral configuration of TNFRS could have been "death-domain less"[27]. tBLASTN searches on the *Daphnia *genome sequence with *Strongylocentrotus *Sp-p75NTR as a query, resulted in two hits. The first was a *Daphnia *sequence on scaffold 9 (Dappu-98375) with a short part of the sequence encoding some of the CRDs but the rest of the sequence encoding no recognizable transmembrane or intracellular domain. The second hit however, was a *Daphnia *sequence with high similarity to p75NTRs. The p75NTR *Daphnia *gene (Dappu-313202), which was named Dappu-p75NTR is on Scaffold 8 (Dappu V1.1 draft genome assembly/scaffold_8:1094314-1104821), and has many of the features of p75NTR as shown in an alignment of *Daphnia*-p75NTR to p75NTR sequences from *Xenopus *(frog) and *Homo *(human), as well as frog NRH1, a vertebrate paralogue of p75NTR (Figure 13). The alignment reveals that Dappu-p75NTR has only three CRDs in its extracellular part, instead of four CRDs commonly found in deuterostomes. Moreover the three CRDs are theoretically encoded by a single exon, suggesting that if differential splicing of the gene should occur, it should not affect the number of CRDs. In this respect, Dappu-p75NTR differs from some vertebrate p75NTRs, such as *Gallus*, *Mus *and *Homo *(depicted respectively as chicken, mouse and human in Figure 13), where the extracellular domain of p75NTR consists of four CRDs that are not encoded by a single exon. In these vertebrates, the N terminal CRD is encoded by exon 2, while the three consecutive CRDs are encoded by exon 3. p75NTRs of *Gallus*, *Mus *and *Homo *can be differentially spliced and the full length p75NTR (FL-p75NTR) can be co-expressed with a shorter p75NTR (s-p75NTR) lacking exon 3, whereby missing three of the four CRDs[43]. The function of s-p75NTR is not well understood. Despite the difference in genomic organization and number of CRDs, *Daphnia *p75NTR has most of the characteristics of a vertebrate p75NTR, including a transmembrane domain with high similarity to the deuterostome p75NTRs. Moreover *Daphnia *p75NTR has a death domain in the intracellular part, which is absent in *Drosophila *Wengen [28]. The search for p75NTR was extended to other protostomes and a p75NTR was found in *Capitella *genome gene predictions (jgi|Capca1|227322|estExt_fgenesh1_pg.C_1670017). The genome sequence was supported by several ESTs ([Genbank: EY550456.1], [Genbank: EY616051.1], [Genbank: EY616050.1], [Genbank:EY550457.1]). Searching gene predictions in *Lottia *also uncovered a p75NTR (jgi|Lotgi1|172616|fgenesh2_pg.C_sca_137000030) and several ESTs also supported the genomic sequence ([GenBank:FC767772.1], [GenBank:FC644258.1], [GenBank:FC591712.1], [GenBank:FC577631.1], [GenBank:FC639457.1], [GenBank:FC573898.1]). The *Capitella *p75NTR is missing parts of the first N terminal CRD. The *Lottia *p75NTR has four CRDs and is very conserved relative to the vertebrates. The finding of p75NTR concomitantly with a Trk and a neurotrophin in representative species of the ecdysozoa and lophotrochozoa within the protostomes, as well as their existence in the deuterostomes suggests that all ligand receptor components for neurotrophin signaling were present before the protostomes and deuterostomes diverged.

#### Evolution of p75NTR in the bilateria

The finding in several protostome representatives of p75NTR, a TNFRSF member endowed with a death domain suggests that death domain TNFRSF could have appeared early in evolution.

While p75NTR was the first TNFRSF member to be discovered, and its sequence characterized the TNFRSF CRDs, the phylogenetic relationship of p75NTR to other TNFRSF is not well understood. p75NTR has several features that distinguish it from other members of the TNFRSF. While TNFRSF proteins bind to tumor necrosis factor superfamily (TNFSF) ligands, p75NTR binds to neurotrophins which are structurally unrelated to TNFSF proteins. In vertebrates, over 20 TNFRSF members have been identified, most of which function in the immune system. The multiplicity of TNFRSF representatives has been suggested to arise from multiple rounds of duplications in concert with the appearance of adaptive immunity in evolution[27]. Aside from functioning in the immune system, p75NTR has a role in ectodermal development and this function is shared by few TNFRSF members such as Troy, and the EDA receptors (EDAR and XEDAR). Sequence alignment indicates that Troy, EDAR and XEDAR fall within a subfamily of structurally similar TNFSF proteins. Although p75NTR does not have greater overall sequence similarity to Troy than to other TNFRSF proteins, p75NTR and Troy have been shown to share a very similar transmembrane domain. Compared to vertebrates, a small fraction of TNFRSF members were identified in invertebrate deuterostomes, such as *Strongylocentrotus *and *Saccoglossus*. Among these, p75NTR, p75NTR-like and Troy sequences were present, suggesting that the duplication separating the p75NTR gene lineage from the Troy/XEDAR/EDAR lineage had already taken place at the root of the deuterostomes[10]. Although the ligand for Troy is unknown, XEDAR and EDAR are known in vertebrates to bind to two different isoforms of EDA, a protein with typical features of the TNFSFs. This may suggest that within the TNFRSF, neurotrophin binding receptors (p75NTR) diverged from portential TNFSF binding receptors at least at the stem of the deuterostome lineage, if not earlier. Prior to the current finding of p75NTRs in protostomes, the only TNFR representative known in protostomes was *Drosophila *Wengen[28]. Wengen has some degree of similarity to p75NTR but also similarity to TNFR1 and XEDAR, which are receptors for TNFSF members. A ligand for Wengen, called Eiger has been reported, and Eiger is most similar to EDA, which is the XEDAR ligand. Eiger is clearly a TNFSF member and is not related to neurotrophins. tBLASTN searches against gene predictions on the *Daphnia *genome sequence with *Drosophila *Wengen as query, yields a hit with a relatively high e-value (0.044) to a gene prediction on scaffold 4 (Dappu-305473). The genomic sequence is supported by several ESTs ([Genbank: FE377316.1], [Genbank: FE348619.1]) and it encodes a TNFR domain protein which like Wengen lacks a death domain. In addition to this gene, partial ESTs in *Daphnia *present high similarity to the Eiger/EDA gene ([Genbank: FE337796.1], [Genbank: FE355401.1]) and encode a TNF domain. The ESTs are represented in the genomic sequence on scaffold 9. Although the evolution of the TNFRSF is beyond the scope of the present study, these preliminary results may suggest that Wengen and p75NTR are paralogues. Since Wengen binds to a TNFSF member, future research will be needed to investigate whether protostomian p75NTRs can indeed bind to protostome neurotrophins. If so, this may indicate that the prerequisites for separate evolution of TNFRSF binding to neurotrophins and TNFRSF binding to TNFSF members were already established before the protostome/deuterostome split.

## Conclusion

In this work, the genome sequence of *Daphnia pulex*[41] provides the first evidence for components of the neurotrophin/Trk/p75NTR signaling system in a protostome. Also, for the first time, *Daphnia *represents an organism where the genome encodes both Spz proteins and a neurotrophin, thus allowing a clarification of the relationship between neurotrophin and Spz proteins as paralogous families. Two new Spz protein families (Spz7 and Spz8) are moreover described, and representatives of these families are found in other crustaceans. Spz7 and Spz8 proteins are represented in *Daphnia *by 14 or more representatives, suggesting that the genes encoding these proteins could be under selective pressure. The *Daphnia *genome also reveals the most conserved Trk relative to the deuterostomes found so far in a protostome. The *Daphnia *Trk is found along side a *Daphnia *Trkl which is orthologous to *Aplysia *Trkl. The search of other genomes and ESTs derived from several protostome species reveals moreover that Trk and Trkl form closely related paralogous families in the protostomes within a "Trk/Trkl family" that is paralogous to NRKs. This highlights a previously unsuspected complexity in Trk and Trk related receptors in protostomes. For the first time, the *Daphnia *genome presents a protostome p75NTR receptor and the p75NTR is subsequently shown to exist in other protostomes. Thus the neurotrophin signaling ligand and receptors were probably already present before the protostome/deuterostome split. The finding in protostomes of p75NTR, which is a TNFRSF member endowed with a death domain, reopens the question as to whether the TNFRSF ancestral configuration was "death domain less" as previously suggested[27]. It will be interesting to see whether a protostomian p75NTR functions in the immune system, the nervous system or both. Moreover, because Wengen, the other known protostomian TNFRSF member binds to a TNFSF ligand, it will be interesting to find out if and how protostomian p75NTRs bind to neurotrophins. Also, in contrast to Trks which bind neurotrophins with high selectivity, p75NTR binds indiscriminately to neurotrophins, so future work may reveal whether p75NTR binds to Spzs. The presence of the neurotrophin/Trk/p75NTR signaling system in *Daphnia *highlights the importance of *Daphnia *for phylogenetic studies, but also as a medical model, since neurotrophins, Trk receptors and p75NTR are involved in the nervous system, the immune system, and in various diseases[44] ranging from Alzheimers[45] to cancer[46,47], including parasite infection[48].

## Methods

### Search for new genes

tBLASTN searches[49] were done against NCBI database ESTs http://www.ncbi.nlm.nih.gov/ and also against the Daphnia pulex, Lottia gigantea, Helobdella robusta and Capitella Sp. I genome data made available from US Department of Energy Joint Genome Institute http://www.jgi.doe.gov/Daphnia[50]. Information available on wFleaBase http://wFleaBase.org was also used. Searches on the Joint Genome Institute (JGI) website were done against all gene prediction models, and gene predictions from different software were aligned to each other as well as to EST sequences when available, to select the best prediction. When the models appeared incorrect based on knowledge of related sequences from the same gene family or superfamily, tBLASTN searches were done directly on the scaffold sequences. The genome browser from the JGI website was used to view the position of identical sequence duplicates on a given scaffold or different scaffolds. Additional analyses of the genes predicted, such as the presence of signature sequences in the translated polypeptide, were sought with prosite http://www.expasy.org/tools/scanprosite/. Domain architecture comparisons were generated by SMART http://smart.embl-heidelberg.de/. Potential signal peptidase cleavage sites on translated gene sequences were mapped with SignalP 3.0 http://www.cbs.dtu.dk/services/SignalP/[51] and prediction of arginine and lysine propeptide cleavage sites was performed using ProP 1.0 http://www.cbs.dtu.dk/services/ProP/[52].

#### Alignment and Phylogenetic analysis

Nucleotide sequences to be used in phylogenetic analysis were translated to amino acid sequences and the amino acid sequences were aligned with MUSCLE[53]. The nucleotide sequences were subsequently aligned by RevTrans[54], using the MUSCLE amino acid alignment as a template. Phylogenetic analyses were performed with two different methods, maximum likelihood (ML) and Bayesian inference (BI). ML tree estimation was done using RaxML-VI v.7.0.0[55,56]. BI analyses were performed using MrBayes 3.1.2 [57,58]. Gaps and N's were treated as missing data.

The ML analysis consisted of 100 independent runs on the original alignment using RAxML under the GTRMIX substitution model (re-estimated all free model parameters) with estimated rearrangement settings, a rate category value of 25 and randomized MP (maximum parsimony) starting trees. This model implies an initial tree inference under the GTRCAT model and thereafter evaluates the final tree topology under the GTRGAMMA model until the likelihood values are stable. Branch support was assessed with 1000 non-parametric bootstrap replicates under the GTRMIX model (random number seed = 180874) and plotted on the ML tree with the best likelihood value. For the BI analyses, MrModeltest v.2 [59] was used for search of best fit models for each codon position; In the RTK tree based on full length sequences (Figure 9A) positions 1 and 2 followed the (GTR+I+G) while position 3 followed the (HKY+I+G) of the Akaike information criterion (AIC). In the RTK tree based on sequences encoding the tyrosine kinase domains only (Figure 9B), positions 1 to 3 followed the (GTR+I+G) model. In the Neurotrophin/Spz tree (with Spz6 included), position 1 and 2 followed the (GTR+I+G) model while position 3 followed (GTR+G) (Figure 1 and Figure 2). The BI analyses consisted of four million generations in two parallel chains executed in three separate runs. The seed number was 180874. The first 2500 trees were discarded as "burn in".

## Authors' contributions

KHSW found the gene sequences, analyzed them and wrote the manuscript.

## Supplementary Material

Additional file 1 **Nucleotide alignment supporting the Spz/neurotrophin tree**. The data represents an alignment of nucleotide sequences encoding the Cys knot of neurotrophins and the C-106 of Spz proteins. This alignment was used to compute the phylogenetic tree presented in Figures 1 and 2. Click here for file

Additional file 2 **Nucleotide alignment supporting the RTK tree with full length sequences**. The data represents an alignment of nucleotide sequences encoding full RTK proteins. This alignment was used to compute the phylogenetic tree presented in Figure 9A. Click here for file

Additional file 3 **Nucleotide alignment supporting the RTK tree with sequence portions encoding the tyrosine kinase domain only**. The data represents an alignment of nucleotide sequences encoding the tyrosine kinase domain of RTK proteins. This alignment was used to compute the phylogenetic tree presented in Figure 9B. Click here for file
