# Supplementary material for: The genome sequence of the protostome Daphnia pulex encodes respective orthologues of a neurotrophin, a Trk and a p75NTR: Evolution of neurotrophin signaling components and related proteins in the bilateria
Source: BMC Evol Biol. 2009 Oct 6;9:243. doi: 10.1186/1471-2148-9-243 (PMC2772990; doi:10.1186/1471-2148-9-243)
Supplement: Additional file 1 — Nucleotide alignment supporting the Spz/neurotrophin tree. The data represents an alignment of nucleotide sequences encoding the Cys knot of neurotrophins and the C-106 of Spz proteins. This alignment was used to compute the phylogenetic tree presented in Figures 1 and 2. [file 1471-2148-9-243-S1.DOC]

CLUSTAL X (1.64b) multiple sequence alignment - created by revtrans

TickRmNT ---------------CAGTGGGAGCAG---------CTGAATCGCACGCAAGACTAC---

TickSpz1 TGCCCCACCAAACGCAAGACCATCTAC---------CCGAAGAGCGCGCAGAACGCG---

TickSpz3 TGTGAGTCCGCCGTGGAGATCGTGACG---------CCGTACTGGGCGTCCAACAGC---

FruitflySpz2 TGCCAGAGCGTGGTACGTTATGCCCGA---------CCCCAGAAGGCCAAGTCAGCT---

DaphniapulexSpz2 TGCCCGTCCGAAGTCAAATACGGTCGA---------CCGCAATTGGCGCAAACCAGC---

FruitflySpz6 TGTCCCGCCAAGGTAGAGTACGCTACT---------CCCGTCTTCGCTAAGAACTAC---

SeaLouseFK884292 TGCCCCTCAGAAATCGCCTACGCCAAA---------ATCCACCGGGCTGTGAACGTT---

FruitflySpz4 TGTCCCGTGAAGGATGAAGTGGTGGCT---------CCCTTTTGGGCAAACAATACC---

FruitflySpz5 TGCCAGACGACATCGCAGTTCATCACC---------CCGCAGGCGGCACTGAATAGC---

FlPrawnBM302838 TGCCCATCTGAGACTGCTTACGTGAGG---------CCCCTTCGTGCCCAGAACACC---

SalmonLouseFK927239 TGTCCCTCTGAGGTCAGTTATTCCAAG---------ATCCATCGTGCTGTCAATGTA---

BrineShrimpES493653 TGCCCTTCTTCTACCAATTATGTTCAA---------CCCCTTCGAGCGGTCAATTCA---

ChickenNT3 TGTGACAGTGAAAGCTTATGGGTCACG------GACAAATCATCCGCTATCGACATT---

DaphniapulexSpz1 TGCGAGTCAATGACGGAATTCAAGTTC---------CCGAAATCGGCCAAGAATAAT---

ChickenBDNF TGTGACAGCACAAGCGAGTGGGTAACAGCAGCGGAGAAAAAGACTGCAGTGGACATG---

FrogBDNF TGTGACAGTATTAGTGAATGGGTTACAGCAGCAAACAAGAAAACTGCAGTGGACATG---

AcornWormNTa TGCCAAGAGATCGGTGATTATGAGTTA---------GTAACCAACGCGATGAACCAA---

SalmonLouseEX480740 TGTCCCTCTGATGCCAAGTATGCTATG---------CCCAGAAGAGCTCGTAATGTC---

FrogNT3 TGTGACAGTGAAAGTTTGTGGGTTACA------GACAAAATGAATGCAATTGACATT---

SalmonLouseFK924306 TGCCCCTCTGATGTCAAGTATGCTATG---------CCCAGAAGAGCTCGTAATGTC---

DaphniapulexSpz7E TGCCCCAGTGATGTTCTTTACGCTCGC---------CCTGTCCGCGCAATCAATGCC---

DaphniamagnBJ928379 TGCCCCAGCGAAACTTCTTACGTCCAG---------CCGCTACGTGCAGTCAACACC---

DaphniapulexSpz6 TGCCCTTCAGCAGCAGAGTACGTCACC---------CCTGTGTACGCCAAGAACTAT---

SeaUrchinNT2 TGCGAGGAAATAAGCCGATGGGAAAAA---------CTCAGCCAGGGCATCAACTTC---

TickIsNT TGCCGGACCAGCACGGCCTGGGAGCGG---------ATGAACAAGACGGTCGACTCG---

DaphniapulexSpz5 TGCCCAACAACATCGTCGTTTATTACA---------CCACGAACTGCTGTCAACACT---

LouseCB886952 TGTCAATCTGAATTTTCTCTCATTGAA---------CCTGGATATGCAAAAGAAGTCACA

DaphniapulexSpz3 TGCGAGTCGACGGTCGAAGTGGTGACT---------CCTTATTGGGCGGCCAACAGC---

AmLobsterEV7819399 TGCCCCTCTGACGTCGCCTACGTCATG---------GCCAAACGTGCCCAGAATGTA---

BrineShrimpES495908 TGTCCATCCAACGTCCAATACGCACGC---------CCTCGTCGTGCCCAGAACACC---

DaphniapulexSpz8C TGCCCGAGTTCTACCGCTTACGTTCAA---------CCTATGCGAGCTGTCAATGTC---

SalmonLouseFK930191 TGTCCCTCTGAGGTCAGTTATTCCAAG---------ATCCATCGTGCTGTCAATGTA---

CapitellaNT TGTCCATCTCGGACTGAGTGGCGTTTG---------ATCCACAGCGCAAGAGACATC---

HumanNGF TGTGACAGTGTCAGCGTGTGGGTTGGG------GATAAGACCACCGCCACAGACATC---

FruitflySpz3 TGCGAGTCGAAGATTGAAATTGTCACA---------CCGTACTGGGCATCGAACTCC---

DaphniapulexSpz7B TGCCCTAGTGACGTTATTTACGCTCGT---------CCTGCTCGCGCAATGAATGTC---

MosquitoeAeSpz5 TGCCAGACGCGTGAGATGTACGTTACG---------CCGCAGGCAGCTTTGAACACC---

MosquitoeAeSpz6 TGTCCAGCCAAGGTTGAGTATGCTACA---------CCGGTGTTCGCTAAGAACTAC---

DaphniapulexSpz7A TGCCCCAGTGACGTCATTTACGCTCGC---------CCTAAACGCGCTTTGAATGTC---

DaphniapulexSpz7F TGCCCCAGCGAGGTGGCCTACTCGCGT---------CCCCTGCGCGCCAAGAACACG---

ZebrafishBDNF TGTGACAGTATTAGCCAGTGGGTGACAGCTGTGGACAAAAAGACGGCAATAGACATG---

DaphniaNT TGCAAAACAAACAGATACTGGAAGCAA---------ATCAACGACACACGAGATATT---

MosquitoeAeSpz3 TGTGAATCCAAGATCGAGATCGTCACA---------CCGTACTGGGCATCCAACTCT---

MosquitoeAeSpz1B TGCCCCAGCGTGGAGAAGTTGGTGCAT---------CCGCAGAGTGGTTATACGGTC---

MosquitoeAeSpz1C TGTGAAAGCGAGCAACTCTTGATCCAC---------CCAAAAGAGGAGCTATCCCGG---

DaphniapulexSpz8F TGCCCGAGTTCCACCGATTACGTCCGT---------CCTTTCCGGGCCATCAACGTC---

SalmonLouseFK929240 TGCCCCTCTGATGTCAAGTATGCTATG---------CCCAGAAGAGCTCGTAATGTC---

DaphniapulexSpz7D TGCCCTAGCGACGTCGTTTATGCTCGC---------CCACTCCGGGCAGTGAATGTC---

SalmonLouseFK924824 TGCCCCTCTGATGTCAAGTATGCTATG---------CCCAGAAGAGCTCGTAATGTC---

FrogNT4/5 TGTGACAGTGTCAACGTCTGGGTTACC------GATAAACGTACAGCCGTGGATGAT---

SalmonLouseFK907691 TGTCCCTCTAGCACAGACTATGTTATG---------CCTCTTCGAGCCATCAACTCT---

DaphniapulexSpz8B TGCCCGAGTTCTACCGCTTACGTTCAA---------CCCATGCGAGCTGTCAATGTC---

ZebrafishNT4/5 TGTGAGGCCGAAAGTGGGTGGGTGACT------AAGAAGAAAACCGCAGTGGACCAT---

DaphniamagnBJ928666 TGCCCATCTGAGACTGCTTACGTGAGG---------CCCCTTCGTGCCCAGAACACC---

DaphniamagnaEG565383 TGCCCCAGCGAAACTTCTTACGTCCAG---------CCGCTACGTGCAGTCAACACC---

ZebrafishNT6/7 TGTGACAGCGAAGAGCACTGGGTTGGC------AACCTGACCCATGCCACAGACTTA---

MosquitoeAgSpz6 TGCCCGGCCAAGGTAGAGTACGCGACG---------CCCGTGTTCGCCAAGAACTAC---

HumanNT3 TGTGACAGTGAGAGTCTGTGGGTGACC------GACAAGTCATCGGCCATCGACATT---

MosquitoeAgSpz4 TGCCCCGTGAAGGAGGAGGTAGTCGCA---------CCGTTCTGGGCCAACAATACC---

ChickenNGF TGTGACAGTGTCAGCATGTGGGTCGGG------GACAAAACCACCGCCACCGACATC---

ZebrafishNT3 TGCGACAGCGAGAGCAATTGGGTTACA------AACAGGACCTCAGCGGTGGATTCT---

FruitflySpzl TGCAGGAGCATCAGGAAGCTGGTGTAC---------CCAAAAAAGGGCTTGAGGGCG---

DaphniapulexSpz8E TGTCCGAGCTCCACCGAATACGTCCGT---------CCTCTACGGGCCATTAACGTC---

AcornwormNTb TGCAGAAGCTCTAATGAGTGGATTCAG---------CTTACACACGGGAGCAACGAA---

HumanNT4/5 TGCGATGCAGTCAGTGGCTGGGTGACA------GACCGCCGGACCGCTGTGGACTTG---

ZebrafishNGF TGCGAGAGCATCAGCCATTGGGAGGGA------AATAAAACCAAAGCCACAGACATC---

LottiaNT TGTCCATCTTCGACCAATTGGAGACTA---------CTGAGATATGCACGTGATATG---

SeaUrchinNT TGTGAGAGTACCAGTGGGTGGATAGTC---------AAAAAATGGGGTACAGACATG---

FrogNGF TGTGACAGCGTCAGTATGTGGGTTGGG------GAAAAGACTACAGCCACCGACATC---

SalmonLouseFK915258 TGCCCCTCTGAAACCAAGTACATTCAA---------CCTCTTAGAGCTGTTAATGCC---

DaphniapulexSpz7C TGCCCTAGCGACGTCGTTTATGCTCGC---------CCACTCCGCGCAGTGAATGTC---

DaphniapulexSpz8A TGCCCCAGCGCCACCTCCTACGTCCAG---------CCGCTGCGTGCCGTCAACACC---

DaphniapulexSpz8G TGCCCGAGTTCCACCGATTACGTCCGT---------CCTTTCCGGGCCATCAACGTC---

MosquitoeAeSpz2 TGCCCGAGCATCATACGCTATGCGCGA---------CCACAGAAGGCCCGTTCCGCT---

HelobdellaNT TGTCCGGTAAAAACTGAATGGTTGAAG---------ATTAAAAGTGCATTCGATATG---

DaphniapulexSpz8D TGCCCCAGTACAACCGGTTATGTCCAA---------CCTCTTCGGGCCATCAACGTC---

HumanBDNF TGTGACAGTATTAGTGAGTGGGTAACGGCAGCAGACAAAAAGACTGCAGTGGACATG---

DaphniapulexSpz8H TGCCCGAGTTCCACCGATTACGTCCGT---------CCTTTCCGGGCCATCAACGTC---

MosquitoeAeSpz1A TGCGAAAGCCGAGAAAGGCTGATACAT---------CCGAGGAGCGGGTTCAACACA---

AmphioxusNT TGCGACGAAAAACAGGAATATAAACAC------ATCAGCGAACCCTTGAACGACTAT---

TickRmNT GACGGCAACGAGGTAGAAATCGTGCAGGAC------------------------------

TickSpz1 GAGAAGGAGTGGCTGTACGTCGTCAACGACGTC---------------------------

TickSpz3 GCTGGCAAGATCAGAGCCATCGTCAACACGCAG---------------------------

FruitflySpz2 TCAGGAGAATGGAAGTACATAGTGAACACTGGT---------------------------

DaphniapulexSpz2 CGTGGCGTCTGGAAATACATCATCAACATGCCC---------------------------

FruitflySpz6 CAAGGCGCCTGGCGTTATGTGGTCCAGATTCCC------------------TATGAGGGC

SeaLouseFK884292 GAAGGCTACTGGAGAATCATTTTGCAACACATGCCCAAGGACTACGCA---TATGGACAG

FruitflySpz4 CGCGGCGAGGTCTTAGCCCTCCTCAATCTCTAT---------------------------

FruitflySpz5 CGCGGAAACTGGATGTTTGTGGTCAACGAGCAGAAC------------------------

FlPrawnBM302838 GAGGGTAAATGGCGTGTGATTGTAAACAATATCGATGCC------------CATTATCAG

SalmonLouseFK927239 GAAGGCTACTGGAGAGTTATTTTACAACATATGCCTACAGACTATGCC---TATGGACAG

BrineShrimpES493653 GAAGGCAAATGGAAAATCATCGTCAACAAAGTCGAAGTC------------AGATACTAC

ChickenNT3 AGAGGACACCAGGTAACTGTGCTGGGAGAAATAAAA---------------ACAGGCAAC

DaphniapulexSpz1 CAAAACGAAATGCTGACCATCGTCAACCAGGAC---------------------------

ChickenBDNF TCTGGTGCAACTGTCACAGTCCTTGAAAAAGTCCCAGTA------------CCTAAAGGC

FrogBDNF TCGGGGCAGACAGTTACTGTCCTAGAAAAAGTCCCAGTA------------TCCAAAGGC

AcornWormNTa TACATGGCGGTAGTCGCTGTC---------------------------------------

SalmonLouseEX480740 AACGGAGAATGGAGAGTCATTGTCAACCACGTTCAC------------------------

FrogNT3 CGAGGACACCAAGTAACTGTGTTGGGGGAAATTAAA---------------ACAGGAAAT

SalmonLouseFK924306 AACGGAGAATGGAGAGTCATTGTCAACCACGTTCAC------------------------

DaphniapulexSpz7E GAAGGTGAGTGGCGCGTCATCGTCCAAGAAATG------------------GCCTGGCCC

DaphniamagnBJ928379 AAAGGCAAATGGCGTGTCATCATCAACAAAGTCGAATCT------------TATGGCATC

DaphniapulexSpz6 CAAGGCGTTTGGCGGTACGTGGTTCAGATCCCC------------------TACGAAGGC

SeaUrchinNT2 CATTCAGCAGAAGTAGACGTCTACGAAGGA------------------------------

TickIsNT TTCGGCAACGCCGTCGAAGTGGTACAGGACGAG---------------------------

DaphniapulexSpz5 CGTGGAAACTGGATGTACGTTGTAAATTTAGAT------------------GGAGAAGAT

LouseCB886952 ACAGGAAAATGGTTTGTCATTGTTCAACATTCAGAA------------------------

DaphniapulexSpz3 TCCGGTAAAATCCGGGCCATTGTTAATACGCAA---------------------------

AmLobsterEV7819399 GAAGGCAAGTGGCGTGTTATTGTCAATGACGTTCAC------------------------

BrineShrimpES495908 AAAGGAGAATGGAGAGTCATCGTTCAAGATGTAGCT------------------------

DaphniapulexSpz8C GACGGAAAGTGGCGCGTTATTGTCAACAAAGTCGAGTCT------------TACAATTAC

SalmonLouseFK930191 GAAGGCTACTGGAGAGTTATTTTACAACATATGCCTACAGACTATGCC---TATGGACAG

CapitellaNT AACGACACAGAAGTTGCGGTGTTCCAACCAGAG------------------TTTGGGGCA

HumanNGF AAGGGCAAGGAGGTGATGGTGTTGGGAGAGGTGAACATT------------AACAACAGT

FruitflySpz3 GCGGGTAAGATCAGAGCCATCGTAAATACTCAG---------------------------

DaphniapulexSpz7B GATGGAGAATGGCGGGTCATCGTCCAGGACATC------------------GCCTGGCCT

MosquitoeAeSpz5 AAGGGCAACTGGATGTACATCGTAAATCACGAG---------------------------

MosquitoeAeSpz6 CAGGGCTCGTGGAGATACGTCGTCCAAATTCCA------------------TACGAGGGA

DaphniapulexSpz7A GATGGTGAGTGGCGCGTGATTGTTCAAGACGTCGCC------------------------

DaphniapulexSpz7F GCCGGTGAGTGGCGCGTCATCGTCCAGGACATC------------------GCCTGGCCG

ZebrafishBDNF TCGGGCCAGACGGTCACCGTTCTGGAGAAGGTCCCCGTG------------ACTAATGGT

DaphniaNT TACGGACAAGAAGTGCGCATCGTTCACAGCTCA---------------------------

MosquitoeAeSpz3 GCGGGAAAAGTACGTGCCATTGTAAATACGCAG---------------------------

MosquitoeAeSpz1B AACGACAAGCTGGTGATGATAGTCAATACACCA---------------------------

MosquitoeAeSpz1C AACAATTCAATGGTGTGGATTGTTAACACGAAA---------------------------

DaphniapulexSpz8F GACGGCAAGTGGCGGACCATCGTCAACGGCGTCGAGTCT------------TACGGCATC

SalmonLouseFK929240 AACGGAGAATGGAGAGTCATTGTCAACCACGTTCAC------------------------

DaphniapulexSpz7D GATGGAGAATGGCGGGTCATCGTCCAAGAAAAC------------------GCCTGGCCC

SalmonLouseFK924824 AACGGAGAATGGAGAGTCATTGTCAACCACGCTCAC------------------------

FrogNT4/5 CGGGGTAAAATAGTGACTGTCATGTCTGAGATTCAGACT------------CTAACAGGA

SalmonLouseFK907691 CAAGGGAAGTGGAGAATCGTTGTGAATAATGTCAAGGCT------------CACTATGAG

DaphniapulexSpz8B GACGGAAAGTGGCGCGTTATTGTCAACAAAGTCGAGTCT------------TACAACTAC

ZebrafishNT4/5 CGTGGAAATAATGTCACTATTATGGATTACATCCCG---------------ACCCAGGCT

DaphniamagnBJ928666 GAGGGTAAATGGCGTGTGATTGTAAACAATATCGATGCC------------CATTATCAG

DaphniamagnaEG565383 AAAGGCAAATGGCGTGTCATCATCAACAAAGTCGAATCT------------TATGGCATC

ZebrafishNT6/7 GGGGGCAATGAAGTCATGGTGCTACCACATTTTCGCATC------------AACAACGTT

MosquitoeAgSpz6 CAAGGCTCGTGGCGCTACGTGGTGCAGATCCCG------------------TACGAGGGC

HumanNT3 CGGGGACACCAGGTCACGGTGCTGGGGGAGATCAAA---------------ACGGGCAAC

MosquitoeAgSpz4 CGGGGGGAGGTGCTCGCACTGCTGAACCTGTAT---------------------------

ChickenNGF AAAGGCAAAGAGGTGACCGTGCTGGGAGAGGTCAACATT------------AACAACAAC

ZebrafishNT3 CGTGGAAATCATGTCACCGTTTTGAATTCCTTC------------------ACTGTAAAA

FruitflySpzl GACGACACCTGGCAGTTAATTGTCAATAACGAT---------------------------

DaphniapulexSpz8E GAAGGCAAGTGGCGGACCATCGTCAATGGAGTAGAGTCC------------TACGGCATC

AcornwormNTb GAGTCGGACTTAGTTGAAGTATACCAA---------------------------------

HumanNT4/5 CGTGGGCGCGAGGTGGAGGTGTTGGGCGAGGTGCCTGCA------------GCTGGCGGC

ZebrafishNGF ACAGGCAACGAGGTCACCGTTTTACCTGACGTAATCATC------------AACAACTCC

LottiaNT AATGATACAGAGGTCGAAGTGTTTCAACCTACA------------------GAAGGCAAT

SeaUrchinNT TATGGACAAAACGTGACCATCCTTTCCGAGATAATGACC------------GCTGGCAAC

FrogNGF AAGGGCAAGGAGGTGACTGTGTTGGGAGAAGTAAAT---------------ATAAACAAT

SalmonLouseFK915258 AACGGTAAATGGAGAATCATTGTCAACAATGTCAAGGCT------------CACTACGAA

DaphniapulexSpz7C GATGGAGAATGGCGGGTCATCGTCCAGGACATC------------------GCCTGGCCC

DaphniapulexSpz8A AAAGGCAAGTGGCGTGTCATCATCAACAAAGTCGAGTCT------------TACGGCATC

DaphniapulexSpz8G GACGGCAAGTGGCGCACCATCGTCAACGGCGTCGAGTCT------------TACGGCATC

MosquitoeAeSpz2 ACCGGCGAGTGGAAGTACATCGTTAACACCGGA---------------------------

HelobdellaNT AACTTCACAAGAGTCGAGATCTACCAGCCCGAA---------------------------

DaphniapulexSpz8D AATGGCAAGTGGCGGACGATCGTCAATCGAGTCGAATCT------------TATGGCATC

HumanBDNF TCGGGCGGGACGGTCACAGTCCTTGAAAAGGTCCCTGTA------------TCAAAAGGC

DaphniapulexSpz8H GACGGCAAGTGGCGGACCATCGTCAACGGCGTCGAATCT------------TACGGCATC

MosquitoeAeSpz1A GATAACAGAACAATCATGATTATTAACACCAAG---------------------------

AmphioxusNT AAAAATCGCGAAGTCGAGGTACTGCAATCTTTTATCACACCTGACTTGAACAATGGGGAA

TickRmNT ---GAGATACAGCAGTACGTGTTCTCGTACCGCTGTGCC---------------------

TickSpz1 ---GAGTACGCTCAGGCTGTCACCACTGAAGTCTGCGGG---------------------

TickSpz3 ---CATCTACAACAGGCCATCCAGCAGGAAGTCTGCCAGTCC------------------

FruitflySpz2 ---CAGCACACGCAAACCTTAAGATTGGAAAAATGCAGT---------------------

DaphniapulexSpz2 ---GAGCACACGCAAACCATACGGATGGAACGATGCCTG---------------------

FruitflySpz6 ---TACTTCACCCAGACGGTGGAGGTGACGCGTTGCATT---------------------

SeaLouseFK884292 TACAACTACACTCAAACCACACGGGTCGAGACCTGCCTC---------------------

FruitflySpz4 ---CCCTTCGAGCAGTACGTCCACTGGGAGAAGTGCACCCACGAG---------------

FruitflySpz5 ---ACCGCTCGCCAAATGGTCAAGGCGGAGCTTTGCGCC---------------------

FlPrawnBM302838 ---ACTCTTACTCAGACTACACGCATCGAAGAGTGCCTG---------------------

SalmonLouseFK927239 TACAATTACACTCAAACAACAAGGGTTGAGACGTGTCTT---------------------

BrineShrimpES493653 ---AAATTCGACCAGAACACACAAGTTGAAGAGTGTGAG---------------------

ChickenNT3 TCTCCAGTTAAGCAATATTTTTATGAAACAAGGTGTAAAGAAGCCAAA------------

DaphniapulexSpz1 ---AACATCTCGCAGCCCATCACCGTGGAAACTTGCCGG---------------------

ChickenBDNF ---CAACTGAAGCAATACTTCTATGAGACCAAATGCAAC---------------------

FrogBDNF ---CAACTGAAGCAATATTTCTACGAGACCAAATGCAAC---------------------

AcornWormNTa ---CATCCAAATACATGGGTTTATACCACTAGATGTTCT---------------------

SalmonLouseEX480740 ---TACTACACTCAAACTACCAGATTCGAAACTTGCTTG---------------------

FrogNT3 TCTCCTGTGAAACAATACTTTTATGAAACAAGGTGTAAAGAGGCAAGA------------

SalmonLouseFK924306 ---TACTACACTCAAACTACCAGATTCGAAACTTGCTTG---------------------

DaphniapulexSpz7E ---GGTTACACCCAGACCCAGCGCACCGAGACGTGCTTG---------------------

DaphniamagnBJ928379 ---CAATACGATCAACATGCTCGCATTGAAGAATGTGAAGATGAT---------------

DaphniapulexSpz6 ---TATTTCACTCAGACAGTTGAAGTAACGAAATGCTTG---------------------

SeaUrchinNT2 ------------CAGTGGTTTTGGGTCACGCGTTGCGTCAGGCAGGTCGATCCCACACAA

TickIsNT ---GCCTTCCCCCAGTGGGTCTTCGCCTACCGTTGCGCC---------------------

DaphniapulexSpz5 CAGCAAAACACACAGCTTGTACGAACTGAGAGATGCGCA---------------------

LouseCB886952 ---ATGTCACAACAAAGAATTACAGCAGACACTTGTAAA---------------------

DaphniapulexSpz3 ---CACTTTGAGCAGGCCGTCCACCAAGAAGTTTGCTCGAAA------------------

AmLobsterEV7819399 ---TACTACACCCAGACCGCCCGCCTGGAGACTTGTCTC---------------------

BrineShrimpES495908 ---TACTACACCCAAACTCAGCGCATGGAAACTTGTCTT---------------------

DaphniapulexSpz8C ---CAGTTCACCCAGACGACCCGCATCGAAGAATGTGATATC------------------

SalmonLouseFK930191 TACAATTACACTCAAACAACAAGGGTTGAGACGTGTCTT---------------------

CapitellaNT ACCCAAGCTCACCAATGGTTCTACACTGTGACTTGCATGAATGATCACCCG---------

HumanNGF ---GTATTCAAACAGTACTTTTTTGAGACCAAGTGCCGGGACCCAAAT------------

FruitflySpz3 ---CATTTCGAGCAGGCGATTCACCAGGAGGTTTGCAGCAAT------------------

DaphniapulexSpz7B ---GGATACACCCAGACTCAACGCTTCGAAACTTGTTTG---------------------

MosquitoeAeSpz5 ---GAATCGCGGCAGTTGGTGAAAGCGGAGATATGCACG---------------------

MosquitoeAeSpz6 ---TACTTTACTCAAACCGTTGAAGTGACTCGTTGTCTC---------------------

DaphniapulexSpz7A ---TACTACACCCAAACCCAGCGCGTCGAGACTTGCTTG---------------------

DaphniapulexSpz7F ---ACTTACACCCAGACCCAGCGGACCGAGACTTGCTTG---------------------

ZebrafishBDNF ---CAGCTGAAGCAATACTTTTACGAGACCAAATGCAAC---------------------

DaphniaNT ---GAATATCGACAGTTTGCCTTCGTTTACGAGTGCATC---------------------

MosquitoeAeSpz3 ---CATTTTGAGCAAGCAATTCACCAGGAGGTGTGTTCAAAA------------------

MosquitoeAeSpz1B ---AATTACATGCAAGGAGTTAGGATAGAAACATGCAGT---------------------

MosquitoeAeSpz1C ---GATTACAAACAAGGCGTTCGAATCGAAAAGTGTCTGAAAAGA---------------

DaphniapulexSpz8F ---AAGTACACGCAAACTGCTCGAATTGAAGAATGCGACGTG------------------

SalmonLouseFK929240 ---TACTACACTCAAACCACCAGATTCGAAACTTGCTTG---------------------

DaphniapulexSpz7D ---GGATACACCCAGACTCAACGCGTCGAAACTTGTTTG---------------------

SalmonLouseFK924824 ---TACTACACTCAAACCACCAGATTCGAAACTTGCTTG---------------------

FrogNT4/5 ---CCACTGAAGCAATACTTCTTTGAGACCAAGTGCAAT---------------------

SalmonLouseFK907691 ---ACTCTAAGTCAAACCGTTCGAGTTGAGCAATGTAGC---------------------

DaphniapulexSpz8B ---CTGTTCACCCAGACGGCCCGCATCGAGGAATGTGACATC------------------

ZebrafishNT4/5 ACGCCACTGAAGCAGTACTTCTATGAAACAAGGTGCCGCGTGTCA---------------

DaphniamagnBJ928666 ---ACTCTTACTCAGACTACACGCATCGAAGAGTGCCTG---------------------

DaphniamagnaEG565383 ---AAATACGATCAACATGCCCGCATTGAAGAATGTGAAGATGAT---------------

ZebrafishNT6/7 ---GTAAAGAAGCAGCTCTTCTACGAGACCACATGTCGTGTGAAGAAACCTATAGGGGCC

MosquitoeAgSpz6 ---TACTTCACGCAGACGGTCGAAGTGACGCGCTGTCTG---------------------

HumanNT3 TCTCCCGTCAAACAATATTTTTATGAAACGCGATGTAAGGAAGCCAGG------------

MosquitoeAgSpz4 ---CCCTTCGAGCAGTACGTGCACTGGGAGAAGTGCACGCACGAG---------------

ChickenNGF ---GTTTTTAAGCAGTACTTTTTCGAGACCAAGTGCAGGGACCCTAGG------------

ZebrafishNT3 CAGAAATACACGCAGTACTTTTATGAGACCAAGTGTAAA---------------------

FruitflySpzl ---GAGTACAAACAGGCCATCCAGATCGAAGAGTGCGAA---------------------

DaphniapulexSpz8E ---AAATACACCCAGACGGCTCGAGTTGAGGAATGTGACGTG------------------

AcornwormNTb ---------AATCAATGGTTCCATGTTACACGTTGTCGT---------------------

HumanNT4/5 AGTCCCCTCCGCCAGTACTTCTTTGAAACCCGCTGCAAGGCTGATAACGCTGAGGAAGGT

ZebrafishNGF ---AAGAAAAAGCAATACTTTTTTGAGACCACCTGCAGCAGCGGGCGA------------

LottiaNT ---GAAGGCTTTCAATGGTTTTACACCGTATCCTGTAATGAACAACATAAAGTAATGACA

SeaUrchinNT ATTCAGGTAACGCAGTGGTTTTACGAGACGGCGTGCGCGCGT------------------

FrogNGF AGTGTTTTCAAACAGTACTTTTTTGAGACCAAATGCAGGGACCCAAAG------------

SalmonLouseFK915258 ---ACCCTTACCCAAACCACCCGTGTTGAACACTGCTCC---------------------

DaphniapulexSpz7C ---GGATACACCCAAACCCATCGCATCGAAAAATGTTTG---------------------

DaphniapulexSpz8A ---AAATACGATCAACACGCTCGCATCGAAGAATGTGAGGCCGAC---------------

DaphniapulexSpz8G ---AAGTACACGCAAACTGCTCGAATTGAAGAATGCGACGTG------------------

MosquitoeAeSpz2 ---GAGCACACGCAAACGCTTCGACTGGAAAAGTGCACG---------------------

HelobdellaNT ---GACGGTTCGCAATGGTATGAGAATGTTTGTTGTACAACTCAACATAAATCA------

DaphniapulexSpz8D ---AAATACACCCAAACGGCTCGAGTTGAAGAATGCAATGTG------------------

HumanBDNF ---CAACTGAAGCAATACTTCTACGAGACCAAGTGCAAT---------------------

DaphniapulexSpz8H ---AAGTACACGCAAACTGCTCGAATCGAAGAATGCGACGTA------------------

MosquitoeAeSpz1A ---GAATATATGCAAGGCGTTCGAATAGAAACATGCAGC---------------------

AmphioxusNT ---GTGATTAATCAGTTCTTCGCGGTGACGACATGCAACGAC------------------

TickRmNT ACTGCACAG---------------------AACCCC------------TGTACAGCCATC

TickSpz1 CAAGAAGAC---------------------ACTCCG------------TGCGAGTTTCTG

TickSpz3 GTGCAGACC---------------------AAAAAG------------TGCAACAATGAC

FruitflySpz2 AATCCCGTC---------------------GAGAGT------------TGTTCCTACTTG

DaphniapulexSpz2 AAACCGGCC---------------------AGCGGT------------TGCAGTTATGTT

FruitflySpz6 CAGGCACGC---------------------------------------TGTCACTACCTG

SeaLouseFK884292 ACTCCAGAC---------------------TCCGCC------------TGCCGACTTCTT

FruitflySpz4 TTCAAGCAA---------------------ATGTTC------------TGCCGCGATGGA

FruitflySpz5 TCGAACACC---------------------------------------TGCTCGAACCTG

FlPrawnBM302838 ACCTCTGGC---------------------GACGCA------------TGCCCTCTGGTG

SalmonLouseFK927239 ACCCCTGAG---------------------TCTGCC------------TGTCGTCTCCTT

BrineShrimpES493653 GCCCCTGAA---------------------CATCCT------------TGTCCACTTGTC

ChickenNT3 CCTGTTAAA---------------------AATGGC------------TGCCGAGGCATT

DaphniapulexSpz1 AATGAAAAC---------------------CAACCG------------TGCAAGTACGCC

ChickenBDNF CCCAAGGGGTACACAAAG------------GAAGGC------------TGCAGGGGCATA

FrogBDNF CCTATGGGTTACATGAAA------------GAAGGC------------TGCAGAGGCATA

AcornWormNTa ACAGTCAAT---------------------ACACCG------------TGCAGAGGATTA

SalmonLouseEX480740 CACGCTGAC---------------------TCTGCC------------TGCAGATTGTTG

FrogNT3 CCTGTCAAA---------------------AATGGC------------TGTCGTGGCATA

SalmonLouseFK924306 CACGCTGAC---------------------TCTGCC------------TGCGGATTGTTG

DaphniapulexSpz7E TTCCCCGGC---------------------TCATCT------------TGCCGCACGTTG

DaphniamagnBJ928379 GCTGCTGGA---------------------AAGGCC------------TGCCCATTGGTG

DaphniapulexSpz6 AAAAAGAAA---------------------------------------TGCCATTTCATG

SeaUrchinNT2 AACGGGTAC---------------------ACGGAA------------TGCTATGGGGTG

TickIsNT ACTCAGGGA---------------------ACGACC------------TGCGTGGGCATC

DaphniapulexSpz5 ACGACAGAA---------------------------------------TGTTCGGGACTC

LouseCB886952 AACCCTGGA---------------------AAACCC------------TGCAGCGGTTTA

DaphniapulexSpz3 CCTCGGACG---------------------GGCCGT------------TGCAATGGCGAC

AmLobsterEV7819399 TTCCCTGAA---------------------GCTGCT------------TGCCGCGCCCTT

BrineShrimpES495908 TTTGCTGGT---------------------GCTGCT------------TGCCGTACATTG

DaphniapulexSpz8C GAAGTCGGT---------------------TCTTCT------------TGTCCATTGGTT

SalmonLouseFK930191 ACCCCTGAG---------------------TCTGCC------------TGTCGTCTTCTT

CapitellaNT GCCATCGAC---------------------CGAGAG------------TGCCCTGGATGC

HumanNGF CCCGTTGAC---------------------AGCGGG------------TGCCGGGGCATT

FruitflySpz3 ACCCAAACT---------------------CCAAGA------------TGTGAGGGCGAG

DaphniapulexSpz7B TTCCCCGGT---------------------GCAGCT------------TGCCGCACCCTG

MosquitoeAeSpz5 TCAAGCGAG---------------------------------------TGCTCCAATCTG

MosquitoeAeSpz6 CAGGCTCGT---------------------------------------TGTCATTATCTA

DaphniapulexSpz7A TTCGCGGGT---------------------GCATCT------------TGCCGTACTTTG

DaphniapulexSpz7F TTCCCCGAA---------------------GCCGCT------------TGCCGCACGTTG

ZebrafishBDNF CCCTTGGGGTACACAAAG------------GAGGGC------------TGCCGAGGAATA

DaphniaNT AGAGAGGGG---------------------GAGCAT------------TGTACAGGCATA

MosquitoeAeSpz3 ACACAAACA---------------------TCTCGC------------TGCTCGGGAGAT

MosquitoeAeSpz1B AGCCCTGGG---------------------AATGCT------------TGCCATAAACTA

MosquitoeAeSpz1C CAATTAGGC---------------------AAGCCG------------TGTAATTTCTGT

DaphniapulexSpz8F GTTGTGGGA---------------------ACCACC------------TGCCCCTTGGTC

SalmonLouseFK929240 CACGCTGAC---------------------TCTGCC------------TGCAGATTGTTG

DaphniapulexSpz7D TTCCCCGGT---------------------GCATCT------------TGTCGCACCTTG

SalmonLouseFK924824 CACGCTGAC---------------------TCTGCC------------TGCAGATTGTTG

FrogNT4/5 CCATCAGGCAGCACCACT------------AGAGGA------------TGCCGAGGTGTA

SalmonLouseFK907691 AAGCAGGGA---------------------ACTCCC------------TGTCCCCTTGTT

DaphniapulexSpz8B GCCATCGGT---------------------TCTTCG------------TGTCCATTGGTC

ZebrafishNT4/5 GGAAACGGCAGTGGGCAAGGGGTGGAGGGGGCAGGC------------TGCTTGGGAGTG

DaphniamagnBJ928666 ACCTCTGGC---------------------GACGCA------------TGCCCTCTGGTG

DaphniamagnaEG565383 GCTGCTGGA---------------------AAGGCC------------TGCCCATTGGTG

ZebrafishNT6/7 CCTAAGCCAGGTCAAGGAGCCAGTGGCGTTAAAGCGGGAACCTCTAGCTGTCGTGGCATT

MosquitoeAgSpz6 CAGGCACGC---------------------------------------TGCCACTATCTG

HumanNT3 CCGGTCAAA---------------------AACGGT------------TGCAGGGGTATT

MosquitoeAgSpz4 CTGAAGCAG---------------------ATGTAC------------TGCCGCGAGGGC

ChickenNGF CCGGTGTCC---------------------AGCGGG------------TGCCGAGGGATC

ZebrafishNT3 ACTCCTCAC---------------------TCCCGCCGCAAGAGCCGATGTTTAGGTATT

FruitflySpzl GGAGCGGAT---------------------CAACCC------------TGTGACTTTGCC

DaphniapulexSpz8E GTTATTGGC---------------------GCTGTC------------TGCCCACTGGTC

AcornwormNTb AACCAAAGC---------------------TCCCCA------------TGCACGGGTATT

HumanNT4/5 GGCCCGGGGGCAGGTGGA------------GGGGGC------------TGCCGGGGAGTG

ZebrafishNGF ACCGGAGGC---------------------TCTGGG------------TGTTTAGGGATC

LottiaNT AACCCTGTA---------------------TGTCCTGGATGT------TGTAGAGGTATC

SeaUrchinNT CCACAAGGC---------------------CTGCACGGCGTGCAAAGGTGCTTAGGGATC

FrogNGF CCAGTTTCA---------------------AGCGGA------------TGCCGTGGGATT

SalmonLouseFK915258 ACCCCTGGA---------------------TCCCAC------------TGTCCTCTTGTA

DaphniapulexSpz7C TTCCCCGGC---------------------GCAGCT------------TGTCGTACCCTG

DaphniapulexSpz8A GCGGCCGGA---------------------AAGGCC------------TGCCCCTTGGTC

DaphniapulexSpz8G GTCGTGGGC---------------------ACCACC------------TGCCCCTTGGTC

MosquitoeAeSpz2 ACACCCCAA---------------------GACAGC------------TGCACTTACCTG

HelobdellaNT CATAACGGG---------------------CGTGTA------------TGTGCT------

DaphniapulexSpz8D GTCGTCGGT---------------------ACAACA------------TGTTCACTGGTC

HumanBDNF CCCATGGGTTACACAAAA------------GAAGGC------------TGCAGGGGCATA

DaphniapulexSpz8H GTCGTGGGC---------------------ACCACC------------TGCCCCTTGGTC

MosquitoeAeSpz1A TCTCAAGGA---------------------CAGCCA------------TGCGTAAAACTG

AmphioxusNT AGTCGTGGATCGGAG---------------GGAGGG------------TGCAGAGGTATC

TickRmNT ---------------TCTGTGCTGTACGAC------------------------AGCGAA

TickSpz1 ---TCGAGCAGCCTGCCCCCGGGCTACACA------------------------TCGGCG

TickSpz3 ------------------------------------------------------TGCAGC

FruitflySpz2 ---------------GCGCAGACTTACCGT------------------------TCCCAC

DaphniapulexSpz2 ---------------TCGCCTCATTACAAA------------------------TCGCAT

FruitflySpz6 ---------------------------GAC------------------------GGCGGT

SeaLouseFK884292 ---------------GCCCCTTGCTACAAA------------------------TCTAAA

FruitflySpz4 ------------------------------------------------------TGCAGA

FruitflySpz5 ------TGCGAATTGCCCAACGGCTACAAC------------------------TCGAGA

FlPrawnBM302838 ---------------CCTGATTGCTACGAG------------------------TCCAAG

SalmonLouseFK927239 ---------------GCACCTTGTTATAAG------------------------TCAAAA

BrineShrimpES493653 ---------------CCAGAATGCTACGAG------------------------TCAAAG

ChickenNT3 ------------GACGACAAGCACTGGAAC------------------------TCCCAG

DaphniapulexSpz1 ------GACAACTTCCCGGCTGGATACAAA------------------------TCATTT

ChickenBDNF ------------GACAAGAGGCACTGGAAC------------------------TCACAG

FrogBDNF ------------GACAAAAGGTACTGGAAC------------------------TCTCAG

AcornWormNTa ------------ACAAGATTTGGTATTAAC------------------------TCAGCG

SalmonLouseEX480740 ---------------GCTCCTTGCTACAAG------------------------TCCAAA

FrogNT3 ------------GACGACAAACACTGGAAT------------------------TCCCAA

SalmonLouseFK924306 ---------------GCTCCTTGCTACAAG------------------------TCCAAA

DaphniapulexSpz7E ---------------GCACCTTGCTATCAG------------------------AGCAAA

DaphniamagnBJ928379 ---------------CCCACCTGTTACGAG------------------------TCGAAA

DaphniapulexSpz6 ---------------------------GAT------------------------GGATCG

SeaUrchinNT2 ------GCTCGGAGTAATGAAATCGCCGAC------------------------GGATCT

TickIsNT ---------------GACGCCCTGTATGAC------------------------AGTGAG

DaphniapulexSpz5 ------TGTCAAGTACCGGCTGGATTTTCT------------------------GCCACC

LouseCB886952 ---------AACGACATGAAATGCATAAAAATGGGTAATTTAACAAACAAAACAACAAAA

DaphniapulexSpz3 ------------------------------------------------------TGCAGT

AmLobsterEV7819399 ---------------GCTCCCTGCTACAAG------------------------AGTCAC

BrineShrimpES495908 ---------------GCCCCATGCTACAAA------------------------AGCCAA

DaphniapulexSpz8C ---------------CCTACTTGCTACGAC------------------------TCCAAA

SalmonLouseFK930191 ---------------GCGCCTTGTTATAAG------------------------TCAAAA

CapitellaNT TGTATTGGCATCAACCACAATAGCTATAGG------------------------TCGCAA

HumanNGF ------------GACTCAAAGCACTGGAAC------------------------TCATAT

FruitflySpz3 ------------------------------------------------------TGTGGA

DaphniapulexSpz7B ---------------GCCCCTTGTTTCGGA------------------------AGCAAA

MosquitoeAeSpz5 ------TGCAGCCTCCCCAATGGGTACAAT------------------------TCCAGG

MosquitoeAeSpz6 ---------------------------GAT------------------------GGTGGC

DaphniapulexSpz7A ---------------GCTCCTTGCCATCGC------------------------AGCTCC

DaphniapulexSpz7F ---------------GCCCCTTGCCACTTG------------------------AGCAAG

ZebrafishBDNF ------------GACAAGCGGCACTATAAC------------------------TCGCAA

DaphniaNT ---------------TCGGAGCACCTACAA------------------------TCGACC

MosquitoeAeSpz3 ------------------------------------------------------TGCGGT

MosquitoeAeSpz1B ------CAGCACCTGATATCATTGTACACC------------------------ACAGAA

MosquitoeAeSpz1C ---------------------GATGCCGAT------------------------ACTGAG

DaphniapulexSpz8F ---------------CCGTCCTGCTACGAG------------------------TCCAAA

SalmonLouseFK929240 ---------------GCTCCTTGCTACAAG------------------------TCCAAA

DaphniapulexSpz7D ---------------GCCCCTTGTTATGGC------------------------AGCAAA

SalmonLouseFK924824 ---------------GCTCCTTGCTACAAG------------------------TCCAAA

FrogNT4/5 ------------GACAAAAAGCAATGGATA------------------------TCTGAG

SalmonLouseFK907691 ---------------CCTGAATGCTACGAA------------------------ACGAAA

DaphniapulexSpz8B ---------------CCTACTTGCTACGAC------------------------TCCAAA

ZebrafishNT4/5 ------------GATAAAAAACACTGGATG------------------------AGCAGG

DaphniamagnBJ928666 ---------------CCTGATTGCTACGAG------------------------TCCAAG

DaphniamagnaEG565383 ---------------CCCACCTGTTACGAG------------------------TCGAAA

ZebrafishNT6/7 ------------GACAGCAAGCACTGGAAC------------------------TCTTAC

MosquitoeAgSpz6 ---------------------------GAC------------------------GCACCG

HumanNT3 ------------GATGATAAACACTGGAAC------------------------TCTCAG

MosquitoeAgSpz4 ------------------------------------------------------TGTCGC

ChickenNGF ------------GATGCGAAGCATTGGAAC------------------------TCTTAC

ZebrafishNT3 ------------GATGAGAAGCACTGGAAT------------------------TCGCAG

FruitflySpzl ------GCCAACTTTCCGCAGAGCTATAAT------------------------CCAATC

DaphniapulexSpz8E ---------------CCGTCCTGCTACGAT------------------------TCCAAA

AcornwormNTb ---------------GCTGAAATGTATACC------------------------TCTAGG

HumanNT4/5 ------------GACAGGAGGCACTGGGTA------------------------TCTGAG

ZebrafishNGF ------------GACGCCCGCCATTGGAAC------------------------TCATAT

LottiaNT ------------AACCATTTCAGATACGCG------------------------TCACAA

SeaUrchinNT ------------GACAACAACAACTACGAC------------------------TCGGTG

FrogNGF ------------GATGCAAAGCATTGGAAC------------------------TCTTAT

SalmonLouseFK915258 ---------------CCCGAATGCTACGAA------------------------ACCAAA

DaphniapulexSpz7C ---------------GCCCCTTGTTTTGGC------------------------AGCAAA

DaphniapulexSpz8A ---------------CCCACCTGCTACGAG------------------------TCCAAG

DaphniapulexSpz8G ---------------CCGTCCTGCTACGAG------------------------TCCAAA

MosquitoeAeSpz2 ---------------ACAGACAATTTCCGC------------------------TCCCGG

HelobdellaNT ---------------------AGATATTAT------------------------TCAAGC

DaphniapulexSpz8D ---------------CCGTCTTGTTACCAG------------------------TCCAAA

HumanBDNF ------------GACAAAAGGCATTGGAAC------------------------TCCCAG

DaphniapulexSpz8H ---------------CCGTCCTGCTACGAG------------------------TCCAAA

MosquitoeAeSpz1A ---------------AATCCCCTGTTCGGAAAA---------------------ACGGAA

AmphioxusNT ------------GACAAAGCACACTGGACG------------------------TCATCG

TickRmNT TGCACAGAACGCAAGGGCTGGATGTACCTCTACTACCGACCACTAGAGGGCGAA------

TickSpz1 TGCCGACAGAAGTTCGCGTATCGGAAACTGCTGGCCCTGCATCCGACAGACAAG------

TickSpz3 TGCGAACAGAAGTACAAGTGGCACCGGTTGCTAGCATACGACCCAGACGACGAC------

FruitflySpz2 TGCTCGCAGGTGTACAACTATCATCGTCTATTAAGTTGGGACAAA---------------

DaphniapulexSpz2 TGCATCCAAGTCTACAATTACCACCGGCTCTTATCGTTCGAAGAACCCAAAGGG------

FruitflySpz6 TGCTTGGCCCACTGCGATGGACACGATGAACTGGGCTGCTTCCAGGTGCGTCTC------

SeaLouseFK884292 TGCACTCAAAAATACGTCTACTACCGCATGGTCTCCCTTGACCCCTGTGATCCC------

FruitflySpz4 TGTGAACAGCAGTACCGCCTCCACAGATTGTTGGCCTACGATCCGCATAACGAG------

FruitflySpz5 TGCGAGCAGAAGTTTGTGCAAAAACGCTTAATTGCGCTCCAGGGCAACGGGCAG------

FlPrawnBM302838 TGCCTGCAGAAGTCCATCTACCACCGCTTCCTCGTCTACGACCCCTATGATCAG------

SalmonLouseFK927239 TGTACTCAAAAGTATGTTTATCATCGCATGGTGTCCCTAGATCCTTGTGATCCT------

BrineShrimpES493653 TGTGTCCAGAAGAATATTTATCATAGATTTCTAGTTTATGACCCATATGACTAT------

ChickenNT3 TGCAAGACATCCCAAACTTACGTTAGAGCATTGACTTCAGAAAACAATAAACTT------

DaphniapulexSpz1 TGCATGCAGAAATACGTCACACATCATTTGGCGGCCCTGAAAAATGGCAAAATT------

ChickenBDNF TGCCGAACTACCCAGTCTTACGTGAGAGCTCTCACCATGGATAACAAAAAGAGA------

FrogBDNF TGCCGAACTACTCAGTCTTACGTGCGGGCTTTCACCATGGATAGCAAAAAAAAA------

AcornWormNTa TGCAAACAAAAGAAGGGATGGGCCCTTGCGTATGTGAAGTCGGTTGAAGACGATCAACCC

SalmonLouseEX480740 TGTACCCAAAAATACGTTTACCAACGCATGGTCTCCTACGATCCCTGTGATCCC------

FrogNT3 TGTAAAACCTCACAAACTTACGTGAGAGCATTGACTTCAGAAAATAACAAAATG------

SalmonLouseFK924306 TGTACCCAAAAATACGTTTACCAACGCATGGTCTCCTACGATCCCTGTGATCCC------

DaphniapulexSpz7E TGCCTGCAAAAATATGTCTACCAGCGAATGCTTTCCTTTGATCCATGCGATCCC------

DaphniamagnBJ928379 TGCCTGCAAAAGAGCATTTACCACCGTTTCCTCGTCTACGATCCCACCGATTAT------

DaphniapulexSpz6 TGTCTAACCTATTGCGACGGTGTCGACGAAATTGGGTGCTATCAGGTTCGTTTG------

SeaUrchinNT2 TGCGTGGAGACAATTGGCTGGATTCTCGCTTATTCAAGATTAAAATCGGATCCC------

TickIsNT TGCACGGAACGACCCGGTTTCATGATTCTGTACCATCGGAAGATCGGCACCGAC------

DaphniapulexSpz5 TGTTCGCAGCAATTTGTACAGAAGCGACTAGTGGCTCTGAATGGAAATGGAGAG------

LouseCB886952 TGTGTTCAAAGATTTTCATATCATCGTCTTATAAGTTGGGATCCGGATGCTCCG------

DaphniapulexSpz3 TGCGAGCAGAAATACAAGTGGCATCGACTCTTGGCTTACGATCCCGACAACGAT------

AmLobsterEV7819399 TGCACCCAGAAGTACGTCTACCACCGCCTCCTGTCCTTCGACCCCTGTGATCCC------

BrineShrimpES495908 TGTCTGCAGAAATATGTTTACCACCGTATGTTGTCTTTCGACCCATGTGACCCA------

DaphniapulexSpz8C TGCGTCCAGAAGAATGTTTTCCATCGTTTCTTGGTCTTTGATCCTTACGATTTT------

SalmonLouseFK930191 TGTACTCAAAAGTATGTTTATCATCGCATGGTGTCCCTAGATCCTTGTGATCCT------

CapitellaNT TGTCGGCAGACGTTTTCACTTGTTTACGCTTTGATCACCAGGAACCGTGGTCAC------

HumanNGF TGTACCACGACTCACACCTTTGTCAAGGCGCTGACCATGGATGGCAAGCAGGCT------

FruitflySpz3 TGCGAGCAGAAATACAAATGGCATAGATTACTCGCCTACGATCCTGATAACGAT------

DaphniapulexSpz7B TGTCTGCAAAAATACGTTTACCAGCGGATGCTTTCCTTCGATCCCTGCGATCCC------

MosquitoeAeSpz5 TGCGAGCAAAAGTTCTCTCAGAAGCGACTTTTAACGCTGGAGGCCGATGGGCAG------

MosquitoeAeSpz6 TGTTTGCAACATTGCGACGGACACGATGAAATTGGGTGCTTCCAAGTTCGCTTG------

DaphniapulexSpz7A TGTGTCCAGAAATACGTCTACCATCGTATGCTTTCCTTCGATCCTTGCAATACC------

DaphniapulexSpz7F TGCCTCCAGAAGCACACCGTCCACCGCATGCTCTCCTTCGACCCGTGCGATGCT------

ZebrafishBDNF TGCCGGACAACCCAGTCTTACGTGCGAGCCCTTACCATGGATAGCAAAAGGAAG------

DaphniaNT TGTGAAATGCGGCCCGGTTGGGCCAACATGTTACACGTCAAATTAACGACAGTCGACACC

MosquitoeAeSpz3 TGCGAGCAAAAGTACAAATGGCATCGGTTACTGGCCTACGATCCGGACAACGAT------

MosquitoeAeSpz1B TGCAAGCAGCTATATCATTATCGAACATTATTAGCATTTGACACGAAAACTAAG------

MosquitoeAeSpz1C TGTAAGCAATTATTCCACTATAGAACACTGGTAGCAGTGGACAAAGTAACGAAA------

DaphniapulexSpz8F TGCGTCCAGAAAAATATTTTCCACCGCTTTTTGGTCTACAACCCACTTGACTAC------

SalmonLouseFK929240 TGTACCCAAAAATACGTTTACCAACGCATGGTCTCCTACGATCCCTGTGATCCC------

DaphniapulexSpz7D TGTCTGCAAAAATATGTTTACCAGCGGATGCTTTCCTTCGATCCTTGCGATCCC------

SalmonLouseFK924824 TGTACCCAAAAATACGTTTACCAACGCATGGTCTCCTACGATCCCTGTGATCCC------

FrogNT4/5 TGCAAAGCAAAACAGTCTTATGTGAGGGCTCTGACCATAGATGCCAACAAGCTT------

SalmonLouseFK907691 TGTGTTCAAAAATCACTCTATCATCGATTCCTTACCTACGATCCCTTTGATTAC------

DaphniapulexSpz8B TGCGTCCAGAAGAACATTTTCCACCGATTCTTGGTCTTCGATCCCTACGATTAT------

ZebrafishNT4/5 TGCGAAACCAAACAGTCATATGTACGTGCCCTCACCTCGAATGTCAACATGAAG------

DaphniamagnBJ928666 TGCCTGCAGAAGTCCATCTACCACCGCTTCCTCGTCTACGACCCCTATGATCAG------

DaphniamagnaEG565383 TGCCTGCAAAAGAGCATTTACCACCGTTTCCTCGTCTACGATCCCACCGATTAT------

ZebrafishNT6/7 TGCACCAACACACACACCTATGTGCGGGCGCTAACGTCCTACAAAAACCAGATC------

MosquitoeAgSpz6 ACAGCGCAGCACTGCGATGGGCACGATGAGATCGGCTGCTTCCAGGTGCGACTG------

HumanNT3 TGCAAAACATCCCAAACCTACGTCCGAGCACTGACTTCAGAGAACAATAAACTC------

MosquitoeAgSpz4 TGCGAGCAGCAGTACCGATTGCACCGGCTGCTCGCCTACGATCCGCACAACGAG------

ChickenNGF TGCACCACGACACACACCTTCGTCAAAGCACTGACCATGGAGGGCAAGCAAGCA------

ZebrafishNT3 TGCAAAACCACGCAGACTTACGTACGAGCGCTTACTCAGCACAAATCGCTGGTT------

FruitflySpzl TGCAAGCAGCACTACACACAGCAGACCCTGGCCAGCATCAAGAGTGATGGCGAA------

DaphniapulexSpz8E TGCATCCAGAAGAATATCTTCCACCGTTTTTTGGTCTTCAATCCCAGCGATTAC------

AcornwormNTb TGCAAAGAAAAACAGTCTTGGACAAACGCATATGTGAAGACCCTCGATTCAGAT------

HumanNT4/5 TGCAAGGCCAAGCAGTCCTATGTGCGGGCATTGACCGCTGATGCCCAGGGCCGT------

ZebrafishNGF TGCACCAATTCGCATACATTTGTACGTGCGCTGACTTCATTCAAGAACCTGGTG------

LottiaNT TGTATGACGAAGAAGGCTTTTGTAATGGCATTAGTTCGGCCGATGAATCAGCAC------

SeaUrchinNT TGCCTGACAAAGTCGGCATGGGTCTACGCCATGATTCGAACAGCGAGAGGAGAG------

FrogNGF TGTACCACCACGCACACCTTTGTCAAAGCATTAACAATGGAAGGGAAGCAAGCA------

SalmonLouseFK915258 TGCGTTCAAAAGTCCATCTACCACCGCTTTTTGACCTGCGACCCTTATGATCAA------

DaphniapulexSpz7C TGTCTGCAAAAATATGTTTACCAGCGGATGCTTTCCTTCGATCCTTGCGATCCC------

DaphniapulexSpz8A TGCCTCCAGAAGAGCATCTACCACCGTTTCCTCGTCTTCGATCCCACCGATTAC------

DaphniapulexSpz8G TGCATCCAGAAGAATATTTTCCATCGCTTCTTGGTCTACAACCCAAAAGACTAC------

MosquitoeAeSpz2 TGCGTACAGATATACAACTATCATCGTTTGCTCAGTTGGGACACG---------------

HelobdellaNT TGCGTTCAAACTCAGTCGTTTGTGAGAGCCCTGATAAAGAGACCGGAAGAGGAG------

DaphniapulexSpz8D TGCGTCCAGAAGACCGTCTTCCATCGATTTTTGGTCTACGATGCCAATGATTAC------

HumanBDNF TGCCGAACTACCCAGTCGTACGTGCGGGCCCTTACCATGGATAGCAAAAAGAGA------

DaphniapulexSpz8H TGCGTCCAGAAGAACATTTTCCATCGCTTCTTGGTCTACAACCCACAAGACTAC------

MosquitoeAeSpz1A TGTCGGCAACTTTACCACTACCGAACGCTACTAGCGATCGATCCCCAAACCAAC------

AmphioxusNT TGTGTGACCAAGAAATCCAATGTTCCTGCAATTGTTAGAATTGGTGGGAAGCTC------

TickRmNT GAGCGAGTAGCAAAGTGGGGCTACGTTTCTGTGAACCACTACTGCGTCTGC

TickSpz1 ------AAGGCCTACGCCGATAACTTCCCGTTCCCGTCGTGCTGCGTGTGT

TickSpz3 TGCAAGGGAATCTTCATGGACTGGTTCCTGTTCCCGTCATGCTGCGTGTGC

FruitflySpz2 GTGCGTGGTCTCCATGTGGATATTTTTAAGGTGCCCACCTGTTGCTCCTGC

DaphniapulexSpz2 ---------ATGCACGTCGATATTTATAAGATTCCCGTTGGATGCAGCTGC

FruitflySpz6 ------------TACTACGACTGGTTCCTCATCCCCGGCTCTTGCAAGTGC

SeaLouseFK884292 TACAGAGGATTCTTCATTGATACCTACAAACTTCCCTCTGCCTGCTCATGC

FruitflySpz4 TGCCGCGGGATATTCTCCGACTGGTTCCGATTCCCGTCCAGCTGCATATGC

FruitflySpz5 ------AATCTGTACACGGACACGTTCTGGTTTCCCAGTTGCTGTGTCTGC

FlPrawnBM302838 TACTTCCCCTTCGCCATCGAGACATTCAAGCTTCCCGCCAGCTGCGCTTGT

SalmonLouseFK927239 TATAGAGGATTCTTCATTGATACATACAAGCTCCCCTCTGCCTGTTCATGT

BrineShrimpES493653 GACTATCCTTTCTCTATCGAGTCTTTCAAACTTCCGTCTGCCTGTTCATGC

ChickenNT3 ---------GTAGGCTGGAGATGGATAAGAATAGACACCTCCTGCGTGTGT

DaphniapulexSpz1 ------------GTGAGAGAAGCCTTCACCTTCCCGTCTTGCTGCGTCTGC

ChickenBDNF ---------GTTGGCTGGCGGTTTATAAGAATAGACACTTCCTGTGTATGT

FrogBDNF ---------GTTGGTTGGCGCTTTATAAGAATAGACACTTCTTGTGTATGT

AcornWormNTa GTGGGAGAATATGACTGGCAATGGATTGCTATTGNACGAGCGTGTAGCTGT

SalmonLouseEX480740 TACAAGGGTCTCTTCATTGACATCTACAAGTTCCCTTCTGCCTGCTCTTGT

FrogNT3 ---------GTGGGTTGGCGGTGGATAAGAATAGACACATCCTGCGTTTGT

SalmonLouseFK924306 TACAAGGGTCTCTTCATTGACATCTACAAGTTCCCTTCTGCTTGCTCTTGT

DaphniapulexSpz7E CAAAAGGGAATCTTCATTGACATCTACAAGTTGCCATCGGCCTGTTCGTGT

DaphniamagnBJ928379 TATTTCCCATTCGCCATTGAAACTTTCAAATTGCCCGCATCTTGCGCCTGT

DaphniapulexSpz6 ------------TACTATGACTGGCTTCTCGTTCCTGGAAGTTGCAAATGC

SeaUrchinNT2 TTTGGAGAGTACGAGTGGGACTACATAGCGGTTCCGAATTGCTGTTCGTGC

TickIsNT GACAACACGCCATCCTGGGGAGCGGTCGAAGTGCCGCATCACTGCACCTGC

DaphniapulexSpz5 ------AATCTTTACACCGACACTTTTTGGTTTCCTCATTGTTGTATCTGC

LouseCB886952 GAACTTTGTCCAAGAATGAGAATTTTTAAATTTCCATCTGCATGTGTTTGT

DaphniapulexSpz3 TGTAAAGGCATTTTCATGGACTGGTTCCTCTTTCCATCCTGTTGCGTCTGC

AmLobsterEV7819399 TACAAGGGTCTCTTCATCGACATCTACAAGATGCCATCTGCCTGCTCTTGC

BrineShrimpES495908 TACAAAGGTCTCTTTATTGACATCTACAAGCTTCCATCTGCTTGCTCTTGT

DaphniapulexSpz8C TACTTTCCATTCGCCATTGAAAATTTCCAACTACCTGCTTCTTGCGGTTGT

SalmonLouseFK930191 TATAGAGGATTTTTCATTGATACATACAAGCTCCCCTTTGCCTGTTCATGT

CapitellaNT ------TCCTTCCTCTGGGCCCCGGTTCAAATCCCGTCGAGCTGTAACTGC

HumanNGF ------------GCCTGGCGGTTTATCCGGATAGATACGGCCTGTGTGTGT

FruitflySpz3 TGCAAGGGCATTTTTATGGATTGGTTCCTGTTTCCTTCGTGCTGTGTCTGT

DaphniapulexSpz7B CAGAAGGGAATCTTCATCGACATCTACAAGTTGCCATCGGCCTGCTCTTGC

MosquitoeAeSpz5 ------AGTTTGTACGTGGACACCTATTGGTTCCCGAGTTGCTGCGTGTGC

MosquitoeAeSpz6 ------------TACTACGATTGGTTCCTGATACCCGGCTCTTGTAAATGC

DaphniapulexSpz7A TACAGAGGCCTGTTCATTGACATTTTCAAATTGCCATCCGCTTGCTCCTGC

DaphniapulexSpz7F TACAAGGGCATCTTCATCGACACCTACAAGTTGCCATCGGCTTGCTCCTGC

ZebrafishBDNF ---------ATCGGCTGGCGGTTTATACGGATAGACACTTCGTGTGTATGC

DaphniaNT AGTTTGGCACCTCAGTGGGGTTACGTCGCTGTACCTCACCACTGCGCTTGC

MosquitoeAeSpz3 TGCAAAGGTATCTTCATGGATTGGTTCCTGTTTCCTTCGTGCTGCGTCTGT

MosquitoeAeSpz1B ------CAACCTTATAAAGAATCATTTCGATTACCATCATGCTGTAAGTGT

MosquitoeAeSpz1C ------AAACCATACAAGGAACAAGTTTTGTTGCCAAGCTGTTGTAAATGT

DaphniapulexSpz8F GCTTTCCCTTTCGCCATTGAAAAATTCAAGTTGCCTGGATCGTGCGCTTGC

SalmonLouseFK929240 TACAAGGGTCTCTTCATTGACATCTACAAGTTCCCTTCTGCTTGCTCTTGT

DaphniapulexSpz7D CAGAAGGGAATCTTCGTCGACATCTACAAGTTGCCATCGGCCTGCTCTTGC

SalmonLouseFK924824 TACAAGGGTCTCTTCATTGACATCTACAAGTTCCCTTCTGCTTGCTCTTGT

FrogNT4/5 ---------GTGGGTTGGCGTTGGATCCGTATTGACACAGCGTGTGTCTGT

SalmonLouseFK907691 TATTTCCCGTTCGCCATTGAGAGTTTCAAACTTCCAGCTTCATGTGCCTGT

DaphniapulexSpz8B TACTTTCCCTTCGCCATTGAAAATTTCCAACTACCTGCTTCTTGCGGTTGT

ZebrafishNT4/5 ---------GTAGGCTGGAGGTGGATCCGTATCAACTCCTCCTGCGTTTGT

DaphniamagnBJ928666 TACTTCCCCTTCGCCATCGAGACATTCAAGCTTCCCGCCAGCTGCGCTTGT

DaphniamagnaEG565383 TATTTCCCATTCGCCATTGAAACTTTCAAATTGCCCGCATCCTGCGCTTGT

ZebrafishNT6/7 ------------GCCTGGAGGTTCATCCGAATCAACGCCGCATGTGTCTGT

MosquitoeAgSpz6 ------------TACTACGACTGGTTCCTCATTCCCGGCTCCTGCAAATGC

HumanNT3 ---------GTGGGCTGGCGGTGGATACGGATAGACACGTCCTGTGTGTGT

MosquitoeAgSpz4 TGCCGTGGCATCTTTTCCGACTGGTTCCGGTTTCCGTCCTGCTGCATCTGC

ChickenNGF ------------GCCTGGAGATTTATCCGGATCGACACAGCCTGTGTGTGT

ZebrafishNT3 ------------TCTTGGAATTGGATACGGATAGACACGGCATGCGTCTGC

FruitflySpzl ---CTGGACGTGGTGCAGAATTCCTTCAAGATCCCCTCCTGCTGCAAGTGC

DaphniapulexSpz8E GCATTTCCTTTCACCATCGAAAAATTCAAGTTGCCTGGCTCGTGCGGATGT

AcornwormNTb ------GCCTATTCGTGGCAGTGGATATCAATCACCACGTGCTGCACCTGT

HumanNT4/5 ---------GTGGGCTGGCGATGGATTCGAATTGACACTGCCTGCGTCTGC

ZebrafishNGF ------------GCGTGGAGACTCATAAGGATCAATGTAGCTTGCGTGTGT

LottiaNT ------TCGTACGACTGGAATTGGATACAAATTGATTCCAGCTGCAATTGC

SeaUrchinNT ---------GAGGGGTGGACGTGGATTGCAATTAGCTCGTCATGCAATTGT

FrogNGF ------------GCATGGAGATTCATACGGATTGATACAGCATGTGTCTGT

SalmonLouseFK915258 TACCTTCCCTTCGCCATCGAAAGCTTCAAGCTCCCAGCCTCTTGTGCTTGT

DaphniapulexSpz7C CAGAAGGGAATCTTCATCGATATCTACAAGTTGCCATCGGCCTGCTCTTGC

DaphniapulexSpz8A TATTTCCCATTCGCCATCGAGACCTTCAAGTTGCCGGCCTCATGCGCCTGC

DaphniapulexSpz8G ACCTTCCCTTTCGCCATTGAAAAATTCAAGTTGCCCGGATCATGTGGATGT

MosquitoeAeSpz2 GCCCGAGGTCTTCACGTGGACATTTTCAAGGTGCCGACATGCTGTTCTTGT

HelobdellaNT ------AGGTACGTCTGGTCCTTCGTCAGAATCAACACCGGCTGTTCATGT

DaphniapulexSpz8D ACTTTCCCCTTTTCCTTGGAGAAATTTAGATTGCCTGGATCTTGCGGATGC

HumanBDNF ---------ATTGGCTGGCGATTCATAAGGATAGACACTTCTTGTGTATGT

DaphniapulexSpz8H ACCTTCCCTTTCGCCATTGAAAAATTCAAATTGCCTGGATCGTGCGGATGC

MosquitoeAeSpz1A ------CAACCTTACAAGGAAAAATTCAAGCTACCTTCGTGTTGCAAATGT

AmphioxusNT ------------GAGTACACATTTATTGCCATTAACACTTCATGCAACTGC
