# Supplementary material for: The genome sequence of the protostome Daphnia pulex encodes respective orthologues of a neurotrophin, a Trk and a p75NTR: Evolution of neurotrophin signaling components and related proteins in the bilateria
Source: BMC Evol Biol. 2009 Oct 6;9:243. doi: 10.1186/1471-2148-9-243 (PMC2772990; doi:10.1186/1471-2148-9-243)
Supplement: Additional file 2 — Nucleotide alignment supporting the RTK tree with full length sequences. The data represents an alignment of nucleotide sequences encoding full RTK proteins. This alignment was used to compute the phylogenetic tree presented in Figure 9A. [file 1471-2148-9-243-S2.DOC]

Query result CLUSTAL X (1.64b) multiple sequence alignment - created by revtrans

AmphioxusTrk ------------------------------------------------------------

DaphniaROR ------------------------------------------------------------

DpulexTrk ------------------------------------------------------------

HumanTrkB ------------------------------------------------------------

DrosophilaROR ------------------------------------------------------------

HumanROR1 ------------------------------------------------------------

HumanTrkA ------------------------------------------------------------

HumanTrkC ------------------------------------------------------------

AplysiaTrkL ------------------------------------------------------------

DaphniaNRK ATGCGTTCGTCGTTGGTGGCATTGTGCTTATTGCTCCTCGTCGTGGATTTCAGCTCGACC

LymneaTrk ------------------------------------------------------------

AmphioxusROR ------------------------------------------------------------

DrosophilaNRK ------------------------------------------------------------

AplysiaROR ------------------------------------------------------------

DaphniaTrkL ------------------------------------------------------------

AmphioxusTrk ------------------------------------------------------------

DaphniaROR ------------------------------------------------------------

DpulexTrk ------------------------------------------------------------

HumanTrkB ------------------------------------------------------------

DrosophilaROR ------------------------------------------------------------

HumanROR1 ------------------------------------------------------------

HumanTrkA ------------------------------------------------------------

HumanTrkC ------------------------------------------------------------

AplysiaTrkL ------------------------------------------------------------

DaphniaNRK ACAGTAGCGGCAACAGCAGAAGTGCAAGATGGTCCAGTTGAGACGGTGGAAGCTGCCGTC

LymneaTrk ------------------------------------------------------------

AmphioxusROR ------------------------------------------------------------

DrosophilaNRK ------------------------------------------------------------

AplysiaROR ------------------------------------------------------------

DaphniaTrkL ------------------------------------------------------------

AmphioxusTrk ------------------------------------------------------------

DaphniaROR ------------------------------------------------------------

DpulexTrk ------------------------------------------------------------

HumanTrkB ------------------------------------------------------------

DrosophilaROR ------------------------------------------------------------

HumanROR1 ---------------------------------------------------------ATG

HumanTrkA ------------------------------------ATGCTGCGAGGCGGACGGCGCGGG

HumanTrkC ------------------------------------------------------------

AplysiaTrkL ------------------------------------------------------------

DaphniaNRK GGCCATGGATTGACGCTGCAGTGTCACCTGCCCGACGTGGCGAATGTGTCCAGTGCTGTG

LymneaTrk ---------------------------------------------------ATGCGAGGT

AmphioxusROR ------------------------------------------------------------

DrosophilaNRK ------------------------------------------------------------

AplysiaROR ---------------------------------------------------ATGTCGTTT

DaphniaTrkL ------------------------------------------------------------

AmphioxusTrk ------------------------------------------------------------

DaphniaROR ------------------------------------------------------------

DpulexTrk ------------------------------------------------------------

HumanTrkB ---------ATGTCGTCCTGGATAAGGTGGCATGGA------------------------

DrosophilaROR ------------------------------------------------------------

HumanROR1 CACCGGCCGCGCCGCCGCGGGACGCGCCCGCCGCTCCTGGCGCTGCTGGCCGCGCTGCTG

HumanTrkA CAGCTTGGCTGGCACAGCTGGGCTGCGGGGCCGGGC------------------------

HumanTrkC ------------------------------------------------------------

AplysiaTrkL ------------------------------------------------------------

DaphniaNRK ACAGTGCGCTGGGAGAGATGGGCAACGTACCATGGCGCCGGTCCCGACGCCAATGAGTCG

LymneaTrk CCTCGCCGGTTCCGGCTGTGGACTCGGGCCAACGTTCTGACTGTCATCAGCATACTCACC

AmphioxusROR ------------------------------------------------------------

DrosophilaNRK ------------------------------------------------------------

AplysiaROR TCTGGACGGATTTTCACTTGGATTTTATTTACCTTCTTGACGGCAGGCGGCTCAATGGTA

DaphniaTrkL ------------------------------------------------------------

AmphioxusTrk ------------------------------------------------------------

DaphniaROR ------------------------------------------------------------

DpulexTrk ------------------------------------------------------------

HumanTrkB ------------------------------------------------------------

DrosophilaROR ------------------------------------------------------------

HumanROR1 CTGGCCGCACGCGGG---------------------------------------------

HumanTrkA ------------------------------------------------------------

HumanTrkC ------------------------------------------------------------

AplysiaTrkL ------------------------------------------------------------

DaphniaNRK AAGCCCCGGACTGGCGAGAGCCTCGTCGTAGCACGGCGTCGTCGCCGCCGCCGCCACCAC

LymneaTrk TCCATCTTGAGTGGCGCCGGCTGCTCGCCGCTCTCACAGCTCCCGTCCGACAACCCAGCA

AmphioxusROR ------------------------------------------------------------

DrosophilaNRK ------------------------------------------------------------

AplysiaROR CGGGGGACCAGTATGACGTCACGCATGTATGCT---------------------------

DaphniaTrkL ------------------------------------------------------------

AmphioxusTrk ------------------------------------------------------------

DaphniaROR ------------------------------------------------------------

DpulexTrk ------------------------------------------------------------

HumanTrkB ------------------------------------------------------------

DrosophilaROR ------------------------------------------------------------

HumanROR1 ------------------------------------------------------------

HumanTrkA ------------------------------------------------------------

HumanTrkC ------------------------------------------------------------

AplysiaTrkL ------------------------------------------------------------

DaphniaNRK CACCACCACCACCGCCGCCGCTCCCGCCGCCTGATCCATCAGGGCTCCGCATTGCGCTTC

LymneaTrk CATGTGGGTGTTCAGGACGGCGTAACGACCGAGCGTGTTGATCGTTCAAAAAATCACCGG

AmphioxusROR ------------------------------------------------------------

DrosophilaNRK ------------------------------------------------------------

AplysiaROR ------------------------------------------------------------

DaphniaTrkL ------------------------------------------------------------

AmphioxusTrk ------------------------------------------------------------

DaphniaROR ------------------------------------------------------------

DpulexTrk ------------------------------------------------------------

HumanTrkB ------------------------------------------------------------

DrosophilaROR ------------------------------------------------------------

HumanROR1 ------------------------------------------------------------

HumanTrkA ------------------------------------------------------------

HumanTrkC ------------------------------------------------------------

AplysiaTrkL ------------------------------------------------------------

DaphniaNRK GCATCCATTTCAGAAGCAGACGAGGGACTGTACAGGTGTGTCGGATCCAGCAGCAGCAGC

LymneaTrk AACACGACCGCGTCGTCGGGGGCG------------------------------------

AmphioxusROR ------------------------------------------------------------

DrosophilaNRK ------------------------------------------------------------

AplysiaROR ------------------------------------------------------------

DaphniaTrkL ------------------------------------------------------------

AmphioxusTrk ------------------------------------------------------------

DaphniaROR ------------------------------------------------------------

DpulexTrk ------------------------------------------------------------

HumanTrkB ------------------------------------------------------------

DrosophilaROR ------------------------------------------------------------

HumanROR1 ------------------------------------------------------------

HumanTrkA ------------------------------------------------------------

HumanTrkC ------------------------------------------------------------

AplysiaTrkL ------------------------------------------------------------

DaphniaNRK AGCAGCTTCATCGATAGCACGTCTGTTGACGAAGGTGGAAGAGGAGAGTTGGACGACCCT

LymneaTrk ------------------------------------------------------------

AmphioxusROR ------------------------------------------------------------

DrosophilaNRK ------------------------------------------------------------

AplysiaROR ------------------------------------------------------------

DaphniaTrkL ------------------------------------------------------------

AmphioxusTrk ------------------------------------------------------------

DaphniaROR ------------------------------------------------------------

DpulexTrk ------------------------------------------------------------

HumanTrkB ------------------------------------------------------------

DrosophilaROR ------------------------------------------------------------

HumanROR1 ------------------------------------------------------------

HumanTrkA ------------------------------------------------------------

HumanTrkC ------------------------------------------------------------

AplysiaTrkL ------------------------------------------------------------

DaphniaNRK CGATCCTTGATACTCGATCATCCCGACGCTGATAACGTTACCATCGTTTCAGCCATTAAC

LymneaTrk ------------------------------------------------------------

AmphioxusROR ------------------------------------------------------------

DrosophilaNRK ------------------------------------------------------------

AplysiaROR ------------------------------------------------------------

DaphniaTrkL ------------------------------------------------------------

AmphioxusTrk ------------------------------------------------------------

DaphniaROR ------------------------------------------------------------

DpulexTrk ------------------------------------------------------------

HumanTrkB ------------------------------------------------------------

DrosophilaROR ------------------------------------------------------------

HumanROR1 ------------------------------------------------------------

HumanTrkA ------------------------------------------------------------

HumanTrkC ------------------------------------------------------------

AplysiaTrkL ------------------------------------------------------------

DaphniaNRK GACACCGAGCAACAAAGCGGAGTAGTAACGAGTCCATCAACTGATGATGGTGGTGATGGT

LymneaTrk ---------------CATCGTGTCACATCAGGTGAACCTCTCGGCGATAGAGTCACCACA

AmphioxusROR ------------------------------------------------------------

DrosophilaNRK ------------------------------------------------------------

AplysiaROR ---------------GTCGTGTCTGTMAGCACTCTGATAGTKTTTGGTCAAGTGAGCTCA

DaphniaTrkL ------------------------------------------------------------

AmphioxusTrk ------------------------ATGGCGAGCCGCAGCGGAAGCTTCCACCTGCTGGTC

DaphniaROR ------------------------------------------------------------

DpulexTrk ---------------------------------ATGGAAACGAAATTTTCAAACCTGATT

HumanTrkB ------------------------------------CCCGCCATGGCGCGGCTCTGGGGC

DrosophilaROR ------------------------------------------------------------

HumanROR1 ---------GCTGCTGCCCAAGAAACAGAGCTGTCAGTCAGTGCTGAATTAGTGCCTACC

HumanTrkA ---------------------------------------------------------AGC

HumanTrkC ---------ATGGATGTCTCTCTTTGCCCAGCCAAGTGTAGTTTCTGGCGGATTTTCTTG

AplysiaTrkL ------------------------------------------------------------

DaphniaNRK GGTGATGATCCCCGTGTTGTAACAACTTCCACCCCCTCGTCCACGCCCGTCTACGGATCT

LymneaTrk CGGTCAACCACTGCACCGGATCAGGTTCCGGGGGACGCGTCACGGAATACGACCATGGCA

AmphioxusROR ------------------------------------------------------------

DrosophilaNRK ------------------------------------------------------------

AplysiaROR CAGGATACCCGTGACAACTCACCAGAGGAGCCGGGAGATATGCTGGACCCATTGATGACC

DaphniaTrkL ------------------------------------------------------------

AmphioxusTrk CTGGCCTGCTGCTGCTGGCTGACTGCCGGAGGAGCGCTGCGGTGCCCCGATGAC------

DaphniaROR ---------------------ATGTTGAAATCATTGAGCAACACGACTCGCGAG------

DpulexTrk ACAGTGATGTTGACGACGATTTTCGTCGGCGGCGATTTGATTAGGGAAACGTCTAGTTCG

HumanTrkB TTCTGCTGGCTGGTTGTGGGCTTCTGGAGGGCCGCTTTCGCCTGTCCCACGTCC------

DrosophilaROR ------------------------------------------------------------

HumanROR1 TCATCATGGAACATCTCAAGTGAACTCAACAAAGATTCTTACCTGACCCTTGATGAACCA

HumanTrkA CTGCTGGCTTGGCTGATACTGGCATCTGCGGGCGCCGCACCCTGCCCCGATGCC------

HumanTrkC CTGGGAAGCGTCTGGCTGGACTATGTGGGCTCCGTGCTGGCTTGCCCTGCAAAT------

AplysiaTrkL ------------------------------------------------------------

DaphniaNRK TTCATCTACTTGCGCGTTCAAGTTGCCGCCGTCCTGACTGACTACCCGGAATCGCCCGTT

LymneaTrk GGGACGAAATGCTCTCTCCAGGTGGATTTGTCCACTTTCGCGTGTCCAGCCGAT------

AmphioxusROR ------------ATGCACAACGTGACGATCGGAATGGGGGATCGCGCCGTGCTG------

DrosophilaNRK ---------ATGGCTGCCGGGCAATGGGTGGGGGTGGTTGAGCGGGTGCTCCGG------

AplysiaROR TTTCTCAAGGAAAGCAGCGGCACTTTCGTTGGCAACGGCTACATCCGGCTGGACTATGTG

DaphniaTrkL ---------------------------ATGTCTCGTCTTGACTGTCCAAGGCAA------

AmphioxusTrk ------------TGCACCTGCCGGGAAGCTAAGGGCGAG------GTGGAGTGTGAGAGA

DaphniaROR ---------------------AGTGGTGAAAGTGTCAAACTGCGGTGCGAGGTGTCGGGC

DpulexTrk ATTTGCAAACTGTGTTGGTGCAGTAGCGCGTCG------------ATAACTTGCGAAGAA

HumanTrkB ------------TGCAAATGCAGTGCCTCTCGG------------ATCTGGTGCAGCGAC

DrosophilaROR ------------------------------------------------------------

HumanROR1 ATGAATAACATCACCACGTCTCTGGGCCAGACAGCAGAACTGCACTGCAAAGTCTCTGGG

HumanTrkA ------------TGCTGCCCCCACGGCTCCTCGGGACTG---CGATGCACCCGGGATGGG

HumanTrkC ------------TGTGTCTGCAGCAAGACTGAGATCAAT------------TGCCGGCGG

AplysiaTrkL ------------------------------------------------------------

DaphniaNRK TTGATCCACCCGCTGAGGCAACAGCGACGCAAGCAGAGACTCAAGTGTTTGGCGTTCGGC

LymneaTrk ------------TGCCAGTGCAACGCAACGAGTGAGGGTATGGTTGTGTCGTGCGTGACC

AmphioxusROR ------------------------------------------CGCTGTAAAGTCGAGGGG

DrosophilaNRK ------------------------------------------------------------

AplysiaROR ATGAAGAACGTGACGAAGTACCGCGGCCAGGCAGTTCGCATCCGCTGCGAGATCACGGGC

DaphniaTrkL ------------------------------------------------------------

AmphioxusTrk CCGAACACCCTG---CGAGCCATCCCCGGTCCTGACGACCTCAACGACATCGACAAA---

DaphniaROR GACCCGCCACCAAACCGCTTTCGCTGGTACAAAAACGAAGCGCCCGTCCTGGAGGAGAAA

DpulexTrk GCCAATAGTTCC---TTGTCACATTTTTACAGCGATGAATCGGATGAGATACAACTGTTA

HumanTrkB CCTTCTCCTGGC---ATCGTGGCATTTCCGAGATTGGAGCCTAACAGTGTAGATCCTGAG

DrosophilaROR ------------------------------------------------------------

HumanROR1 AATCCACCTCCC---ACCATCCGCTGGTTCAAAAATGATGCTCCTGTGGTCCAGGAGCCC

HumanTrkA GCCCTGGATAGC---CTCCACCACCTGCCCGGCGCAGAGAAC------------------

HumanTrkC CCGGACGATGGG---AACCTCTTCCCCCTCCTGGAAGGGCAGGATTCAGGGAACAGCAAT

AplysiaTrkL ------------------------------------------------------------

DaphniaNRK CATCCGACGCCC---GTCATCCATTGGCGAATCAACAACAACGAGAGTCTCCACCAGCAG

LymneaTrk CCTGACACGCTA---CGGGAATTTCCAGTTATCGCGAGAGAAGTTGCCAGAGCT------

AmphioxusROR GTTCCGGCGCCG---AACTTCCGCTGGTACAAAAACGACGCGCCGCTGACCTCGGAGCGG

DrosophilaNRK ------------------------------------------------------------

AplysiaROR AACCCCATCCCC---AACTACAGCTGGTACAAGGATGACGTCATCATCAACAATGACCCC

DaphniaTrkL ------------------------------------------------------------

AmphioxusTrk ------------------------------------------------------------

DaphniaROR GGCCGGGTGGTCGTCCGAAAGTACCGC---------------------------------

DpulexTrk AAT---------------------------------------------------------

HumanTrkB AAC---------------------------------------------------------

DrosophilaROR ------------------------------------------------------------

HumanROR1 CGGAGGCTCTCC------------------------------------------------

HumanTrkA ------------------------------------------------------------

HumanTrkC GGGAACGCCAATATCAACATCACGGACATCTCAAGGAAT---------------------

AplysiaTrkL ------------------------------------------------------------

DaphniaNRK GTGCGCTTGGTCGAGGAGGCGGACGACGAGCCAATAAGACGGCCTCAACGAGGTTACATC

LymneaTrk ------------------------------------------------------------

AmphioxusROR CGGAGGATACAG------------------------------------------------

DrosophilaNRK ------------------------------------------------------------

AplysiaROR AGCGACCGCAGGATGGGA------------------------------------------

DaphniaTrkL ------------------------------------------------------------

AmphioxusTrk ------GTCAAAGAGCTGTACATCAGCGGCCAACAGGGTCTGACCAGACTAACAAGAGAA

DaphniaROR ------ACCGGATCGTCCATCCACGGCAGCAGACTGCGCATCTCTGACGTCGACACCCAC

DpulexTrk ------GTCACTTACATATACATTCAAAATCAGACAAATCTGACGTCTCTCGAGAAATCG

HumanTrkB ------ATCACCGAAATTTTCATCGCAAACCAGAAAAGGTTAGAAATCATCAACGAAGAT

DrosophilaROR ---------------------------------------------------ATGAACAAA

HumanROR1 ------TTTCGGTCCACCATCTATGGCTCTCGGCTGCGGATTAGAAACCTCGACACCACA

HumanTrkA ------CTGACTGAGCTCTACATCGAGAACCAGCAGCATCTGCAGCATCTGGAGCTCCGT

HumanTrkC ------ATCACTTCCATACACATAGAGAACTGGCGCAGTCTTCACACGCTCAACGCCGTG

AplysiaTrkL ---------------------ATGGTGGAGAACGTCACGGTGCCTTCCTTGGATCTTGGC

DaphniaNRK CGCTCCGTAGTTGCAATTGACTGGGAGAGCAGTCGACAGGGCGACCAAGAAAACGACCAG

LymneaTrk ------GTCATCAAACTAGAGCTACGAGGACAATCCAAGCTGACGTCTCTTAAAACT---

AmphioxusROR ------ATCCGGAACTACAGCTGGGGGTCAAGGTTACGGATTAAGAAGGTCGACACGCAC

DrosophilaNRK ------GGAATGGTGCTGAAATGGGGGGCCAATTTGGCTGTCCTGGGGCTGTGCGTG---

AplysiaROR ------CACAAACCCACGGCCTGGGGTTCTAGACTGAAGATCAACGATGTACGACCTTCC

DaphniaTrkL ------------------------------------------------------AAAGAA

AmphioxusTrk CAGCTGGGGCACTACAAAGGT---------CTGATCAAACTTGTTATCAAGAATAGCAAC

DaphniaROR GACACTGGCTACTACAAATGCGAAGCCTCCAACGGCAAAGAACGCGTCGAGTCCACCGGC

DpulexTrk TCTTTGAAACCCTTCAGTCAA---------CTCATAGAATTAACGATCATCGATTGCGGT

HumanTrkB GATGTTGAAGCTTATGTGGGA---------CTGAGAAATCTGACAATTGTGGATTCTGGA

DrosophilaROR TACTCGGCATTTATAGTCTGCATTTCGCTCGTGCTTTTATTTACAAAAAAGGATGTGGGG

HumanROR1 GACACAGGCTACTTCCAGTGCGTGGCAACAAACGGCAAGGAGGTGGTTTCTTCCACTGGA

HumanTrkA GATCTGAGGGGCCTGGGGGAG---------CTGAGAAACCTCACCATCGTGAAGAGTGGT

HumanTrkC GACATGGAGCTCTACACCGGA---------CTTCAAAAGCTGACCATCAAGAACTCAGGA

AplysiaTrkL AGGCTATTTCCCTTCCTGGGACCAACCATGTCCTCTGATATAATTCCTATGGTTGGGAAC

DaphniaNRK GACCAGGACCAGGACGACGACGACGACGACAACAACTGGCAACAGACGGTGAACGTGACT

LymneaTrk GAACTGAAATTCTTCACTTGT---------CTCAAGCATTTAACTATTGAGAACTGTGGA

AmphioxusROR GACACGGGATACTACCGCTGTGTCGCCACCAACGGGCAGGACAGGGTCTCCACACAGGCC

DrosophilaNRK ------------------------------TTTCTCTTTGCCAGCGCCACGCACGCGAAC

AplysiaROR GACTCTGCAGTGTACACTTGCAAGGCTGAGAACGACTTTGGCAACGAGGAGACAAGTGGC

DaphniaTrkL AAAGAAGAAGCGCTGATGGAGCCAGCTGTCAACAGCTGGGCCCGTCCCGCCCGAAATATA

AmphioxusTrk CTGTCTACGATT------------------------------------------------

DaphniaROR ATCGTCATCGTC------------------------------------------------

DpulexTrk CTTCGTTACATA------------------------------------------------

HumanTrkB TTAAAATTTGTG------------------------------------------------

DrosophilaROR AGCCATAATGTG------------------------------------------------

HumanROR1 GTCTTGTTTGTC------------------------------------------------

HumanTrkA CTCCGTTTCGTG------------------------------------------------

HumanTrkC CTTCGGAGCATT------------------------------------------------

AplysiaTrkL AGGCTTGGTGGA------------------------------------------------

DaphniaNRK TGCTCGGCTAGC------------------------------------------------

LymneaTrk CTGAATAACATA------------------------------------------------

AmphioxusROR ATCCTCTACGTC------------------------------------------------

DrosophilaNRK TCCCTGAACGCC------------------------------------------------

AplysiaROR TCTCTCACTGTCCTCAACGAAAATCCTCCGCCCTCCAAGTCTCAAGGTGGCAGCAACAAC

DaphniaTrkL ACAGCGTGTGTT------------------------------------------------

AmphioxusTrk GAAGATGGCGCCTTCAAGAACAACAGTAACCTTACAATGATTGAT---CTACGAAATAAC

DaphniaROR ------------CGCATGGGTAACCATCCTTCCCGTCTTTCGGGGGAGTTCCCGTCAGGT

DpulexTrk AGTGCAGACGCTTTTGAAGGCAACCCATTACTGAAGAAATTAAAT---TTTCAAAACAAT

HumanTrkB GCTCATAAAGCATTTCTGAAAAACAGCAACCTGCAGCACATCAAT---TTTACCCGAAAC

DrosophilaROR ---------------------------GACTCAAGAATATATGGT---TTCCAGCAATCA

HumanROR1 AAGTTTGGCCCCCCTCCCACTGCAAGTCCAGGA---------------TACTCAGATGAG

HumanTrkA GCGCCAGATGCCTTCCATTTCACTCCTCGGCTCAGTCGCCTGAAT---CTCTCCTTCAAC

HumanTrkC CAGCCCAGAGCCTTTGCCAAGAACCCCCATTTGCGTTATATAAAC---CTGTCAAGTAAC

AplysiaTrkL AGAGAGGACAAACTCCCCGCTTCAATGGACACCAAGATCAAACAG------AGCCGAGAA

DaphniaNRK AACACCATCGCCCGTCAAACGCACACCGACACGCGAATGTTCAAC---TTGGAGCGAGTC

LymneaTrk CAAGGCATCGCATTCAAAACTCTCACCAGTCTGGAAACTATAAAT---TTACGTCATAAC

AmphioxusROR AGATACATGGCAGTCCATAGCAACTACCGGTATTCTCTATGTAAAGTACATGGACGGGGT

DrosophilaNRK ATCGAGGAGCCCGTCACCCGGCGACACCACCAGCGGCAT---------CACGAGCGCGAG

AplysiaROR AACGATGACGACTACCCCACTGACACTGACGTGGTGGAAGGTGGAGAGTTCAAACGAAAG

DaphniaTrkL TTGATAGCCCGACCAACCAGCAATCCTCACATGAGACATATT------------------

AmphioxusTrk AAACTGTCAGTG------------------------------------------------

DaphniaROR CCGCTAGAA---------------------------------------------------

DpulexTrk CAGCTCACCGTG------------------------------------------------

HumanTrkB AAACTGACGAGT------------------------------------------------

DrosophilaROR TCA---------------------------------------------------------

HumanROR1 TATGAAGAAGAT------------------------------------------------

HumanTrkA GCTCTGGAGTCT------------------------------------------------

HumanTrkC CGGCTCACCACA------------------------------------------------

AplysiaTrkL AAGAGCCACGGA------------------------------------------------

DaphniaNRK CGTCCAGGCGACGTCAGAGGTGAACAGGAGGATGAAGCCAACGACAATCAGGCCGGCGCC

LymneaTrk CATTTAACAGAA------------------------------------------------

AmphioxusROR ------------------------------------------------------------

DrosophilaNRK CGGGAGGAGAAC------------------------------------------------

AplysiaROR TACGACACAAGTGTGTGGCCAGACAAAGACGATGTGGAAGACGATGACAAGCCAAGAGAC

DaphniaTrkL ------------------------------------------------------------

AmphioxusTrk CTGCAGTGGACATTGTTCTCCGACCTGAGGCTTACAGAACTGTTGCTAAGC---GACAAC

DaphniaROR GGATTCTGTCAAGTGTACCGCGGAGCAACGTGTTCCAAGTTTCTAGCCAAT---CGCACC

DpulexTrk GTACCGTGGACATTGTTCGTTTCCTTGAACAGCCCTGAAATAAATTTGATC---AAAAAC

HumanTrkB TTGTCTAGGAAACATTTCCGTCACCTTGACTTGTCTGAACTGATCCTGGTG---GGCAAT

DrosophilaROR GGTATTTGCCATATTTACAATGGCACCATTTGTCGCGATGTCTTGAGCAAT---GCCCAT

HumanROR1 GGATTCTGTCAGCCATACAGAGGGATTGCATGTGCAAGATTTATTGGCAAC---CGCACC

HumanTrkA CTCTCCTGGAAAACTGTGCAGGGCCTCTCCTTACAGGAACTGGTCCTGTCG---GGGAAC

HumanTrkC CTCTCGTGGCAGCTCTTCCAGACGCTGAGTCTTCGGGAATTGCAGTTGGAG---CAGAAC

AplysiaTrkL GGAGATAAAGGCCCTTTATTGAAGGCCAAGGTCAAGCATAGAGTTCACAAAAATGACAAT

DaphniaNRK GGCTATTGCGGACGCTACACGGGCAACATTTGCCGGGGACAGCTGAGCAATCCGGCCTCC

LymneaTrk TTTCCCCAAGAGCTGTTAAGGACCCTTAATCTCCGAGAATTGTGGCTTGAG---GGAAAC

AmphioxusROR GGTTACTGCCAGCAGTACCGCGGAGCTGCCTGTCGCAAGTTCATCGGGAAC---GCAACG

DrosophilaNRK GGCTACTGCGCCCCGTACAGCGGCAAGGTGTGCAAGGAATACCTCACC------GGCCAG

AplysiaROR GGATTCTGCCAGATCTACAGAGGATCCACCTGTGCCAAGTTCGTTGGCAAC---ATGAGC

DaphniaTrkL TTGTTTTACAACGAAGAGAAGAAGAAGAAGACCCCGACCGTGTTGTCGACTCTCTCACAA

AmphioxusTrk CCGCTGGCCTGCAACTGCAGCTCCAAGTGGATACAGCTCTGGCAAAGCCAGTCGAGAGCC

DaphniaROR ATCTTCGTCCAGTCGTCTCTGACG---------CAAGGCATCGTCGAGGAAAAATTAGCG

DpulexTrk CCATTGAGTTGCGTCTGCGAAAATAAGTGGATCTTGGAAACCGCCCGGAATTCCGCCGAA

HumanTrkB CCATTTACATGCTCCTGTGAC------------ATTATGTGGATCAAGACTCTCCAAGAG

DrosophilaROR GTTTTCGTATCCCCCAATCTCACC---------ATGAACGATTTGGAGGAGCGATTAAAG

HumanROR1 GTCTATATGGAGTCTTTGCACATG---------CAAGGGGAAATAGAAAATCAGATCACA

HumanTrkA CCTCTGCACTGTTCTTGTGCCCTGCGCTGGCTACAGCGCTGGGAGGAGGAGGGACTGGGC

HumanTrkC TTTTTCAACTGCAGCTGTGACATCCGCTGGATGCAGCTCTGGCAGGAGCAGGGGGAGGCC

AplysiaTrkL GTGATGCACCCTGGGCAT---------------CCAAGCAGACTAGGCCAGGGTGCTGGT

DaphniaNRK GTGTGGTACAACATCAGCAGCAACGACCAGACGGGCGGCTGGCTCAACGAGCAGCTCGTC

LymneaTrk GCATTGACTTGCAGCTGCACT------------AACTTGTGGCTTAGAAGCGTAGACGTA

AmphioxusROR ATTTACATGGACACTCTCCGGGCG---------CAGGGCATCATCGAGAACCAGCTGACG

DrosophilaNRK GTGTGGTACAGTCTGGAGGATCCCACT------GGCGGGTGGAAGAACGAGCAGGTGACC

AplysiaROR ATCTACGTCACCTCCAAGCTCACT---------CAGAGTCGCGCCGAGGAGAAATATATG

DaphniaTrkL GCTGTAACAACCTTGGCGAGTGTGATCGCCATG---------------------------

AmphioxusTrk AGT------------------------TTTCATACCGAGAAGATCTCCTGCATAACA---

DaphniaROR GCCGCTTTC---------------ACCGTTATCGCACATTCTGGGGATCTGTCGCAAAGC

DpulexTrk TCTAAAACAACCCCGTACGTGGCGGGATGGCCCCGAATCAAATCATTTTCTCTTCAAAGT

HumanTrkB GCTAAATCC---------------AGTCCAGACACTCAGGATTTGTACTGCCTGAATGAA

DrosophilaROR GCAGCTTAT---------------GGAGTAATCAAGGAATCCAAGGATATGAACGCAAAT

HumanROR1 GCTGCCTTC---------------ACTATGATTGGCACTTCCAGTCACTTATCTGATAAG

HumanTrkA GGA------------------------GTGCCTGAACAGAAGCTGCAGTGTCATGGGCAA

HumanTrkC AAG------------------------CTCAACAGCCAGAACCTCTACTGCATCAAT---

AplysiaTrkL GCTGCTTCT---------------GGTGCTGTTGGTGCTGTTGCTGGTGATAGCGGAGGC

DaphniaNRK CACGGACTC------------------TGGGACGAAGTTGTCAATACTCTGCGGGAGCCG

LymneaTrk GCT------------------------GCAGATCGTTCAGAAATGACCTGTTCAACACGG

AmphioxusROR GCAGCGTTC---------------ACGGTGATCGGCACATCGAGCGACCTGACGAAGCGG

DrosophilaNRK ACGGCGCTC------------------TGGGACGAGCTTATCTCCGATCTTACGGGTCTG

AplysiaROR GCTGCCTTC---------------GCGGTGATCCAGGCGTCTAGCCACATGAGCCAGCGT

DaphniaTrkL ---------------------------GCGCCACAGAAGATCGTCAACGCAACAGATCCG

AmphioxusTrk ------ACAACAGACGAAACCGTAGACATCAGGGACTTA------CAGCTGGATTCTAGC

DaphniaROR ------TGTGCTGAATTCGCCGTGCCATCGCTCTGCTTC------GCCGCCTTTCCACTC

DpulexTrk TTTATTTGTATCGATTCCGGGGGTAGCAGTCATTCGCTACTAGAATACACTTTCGATGAA

HumanTrkB ------AGCAGC---AAGAATATTCCCCTGGCAAACCTG------CAGATA---CCCAAT

DrosophilaROR ------TGCCGCATGTACGCTTTGCCCAGCTTGTGTTTC------AGTTCAATGCCAATT

HumanROR1 ------TGTTCTCAGTTCGCCATTCCTTCCCTGTGCCAC------TATGCCTTCCCGTAC

HumanTrkA ------GGGCCCCTGGCCCACATGCCCAAT------------------------GCCAGC

HumanTrkC ------GCTGATGGCTCCCAGCTTCCTCTCTTCCGCATG------AACATC---AGTCAG

AplysiaTrkL ------GGAGCGCACGGAGGTGGTTCAGTGGTCTGTACG------TCCATC---GCCGAC

DaphniaNRK ------TGTAAATCAGCGGCCAAGAAATTGCTGTGCTTG------TACGCTTTCCCCGAG

LymneaTrk ------GACGGTGTCAGTAAGATGAAGATGACTCAGTTC------AAGTGCGAACCA---

AmphioxusROR ------TGCGCGGACTACGCCATCCCCTCGCTGTGCCAC------TACGCCTTCAAGTAC

DrosophilaNRK ------TGTCGCGAAGCAGCCGAGAAAATGCTCTGCGCC------TATGCGTTTCCCAAC

AplysiaROR ------TGCCAGCAGTATGGGATCCAGTCCCTGTGCTAC------CATGCGTTCCCTCTG

DaphniaTrkL ------AACCAGGGACGGGCAGGTGCTCGGGATTGCGAT------TCGGCT------CGT

AmphioxusTrk TGTCGTCTACCAACG---------------------------------------------

DaphniaROR TGCGACGACCAGGGG---------------------------------------------

DpulexTrk TGCGATGTACCGGAA---------------------------------------------

HumanTrkB TGTGGTTTGCCATCT---------------------------------------------

DrosophilaROR TGCCGGACTCCAGAGCGCACGAATCTCTTGTACTTCGCCAACGTGGCCACAAATGCCAAG

HumanROR1 TGCGATGAAACTTCA---------------------------------------------

HumanTrkA TGTGGTGTGCCCACG---------------------------------------------

HumanTrkC TGTGACCTTCCTGAG---------------------------------------------

AplysiaTrkL TGCGGGCCG---------------------------------------------------

DaphniaNRK TGTCACCTGGACGAG---------------------------------------------

LymneaTrk TGTGGAATTCCAGAT---------------------------------------------

AmphioxusROR TGCGACGCCCACTTC---------------------------------------------

DrosophilaNRK TGCCACATGGAGGGC---------------------------------------------

AplysiaROR TGCGACAAGACAGCG---------------------------------------------

DaphniaTrkL TGCGGGATGGACGCC---------------------------------------------

AmphioxusTrk ------------------------------------------------------------

DaphniaROR ------------------------------------------------------------

DpulexTrk ------------------------------------------------------------

HumanTrkB ------------------------------------------------------------

DrosophilaROR CAACTGAAGAACGTCAGCATTCGACGGAAGAGAACCAAGTCCAAGGACATTAAGAACATA

HumanROR1 ------------------------------------------------------------

HumanTrkA ------------------------------------------------------------

HumanTrkC ------------------------------------------------------------

AplysiaTrkL ------------------------------------------------------------

DaphniaNRK ------------------------------------------------------------

LymneaTrk ------------------------------------------------------------

AmphioxusROR ------------------------------------------------------------

DrosophilaNRK ------------------------------------------------------------

AplysiaROR ------------------------------------------------------------

DaphniaTrkL ------------------------------------------------------------

AmphioxusTrk ---------------------------------------------------------GCT

DaphniaROR ------------------------------------------------------------

DpulexTrk ---------------------------------------------------------ATC

HumanTrkB ---------------------------------------------------------GCA

DrosophilaROR AGCATATTCAAGAAGAAGTCCACCATCTACGAGGATGTGTTCAGCACAGACATATCGAGT

HumanROR1 ------------------------------------------------------------

HumanTrkA ---------------------------------------------------------CTG

HumanTrkC ---------------------------------------------------------ATC

AplysiaTrkL ------------------------------------------------------------

DaphniaNRK ---------------------------------------------------------GAT

LymneaTrk ------------------------------------------------------------

AmphioxusROR ------------------------------------------------------------

DrosophilaNRK ------------------------------------------------------------

AplysiaROR ------------------------------------------------------------

DaphniaTrkL ------------------------------------------------------------

AmphioxusTrk AGAGTGCTGACCGGGGAAGTGACCATTAACAAGACACAAGACGTAGTGGTGAGCTGTGAA

DaphniaROR GGTAAACCCGTTCCT------------------CGTCAGCTCTGCCGCGATGAGTGCGAG

DpulexTrk TTTATGAACAAAAGCGATATTCAACTCAACGAAAAAGAATCACTCGCCATCGTATGTGCA

HumanTrkB AATCTGGCCGCACCTAACCTCACTGTGGAGGAAGGAAAGTCTATCACATTATCCTGTAGT

DrosophilaROR AAATACCCACCAACCAGAGAGTCTGAGAACCTAAAACGCATTTGCCGCGAAGAGTGCGAA

HumanROR1 TCCGTCCCAAAGCCC------------------CGTGACTTGTGTCGCGATGAATGTGAA

HumanTrkA AAGGTCCAGGTGCCCAATGCCTCGGTGGATGTGGGGGACGACGTGCTGCTGCGGTGCCAG

HumanTrkC AGCGTGAGCCACGTCAACCTGACCGTACGAGAGGGTGACAATGCTGTTATCACTTGCAAT

AplysiaTrkL ---------------------------------GGTGCGGAGTGTGTTGCCCTGCCAGAC

DaphniaNRK GGATTCGCCAGGAAG------------------TTGCCGCTCTGCTACGAGGATTGTATG

LymneaTrk ------------------------------------------------------------

AmphioxusROR CCGTACCCGCAGCCG------------------CGCCAGCTGTGCCGCGACGAGTGCGAG

DrosophilaNRK GGTCGAGCGGTGAAG------------------GCTCCTCTCTGCTTCGAGGATTGCCAG

AplysiaROR GACAGGCCGACCCCG------------------AGGAAGATCTGTAGGGATGAGTGTCTG

DaphniaTrkL ---------------------------------------GGCTGTCGTCAGGTGCCCGAC

AmphioxusTrk GCCTCGGGCAGCCCCGCGCCGACTGCCGAGTGGAAC---------ACAGCAGAGCTTCGC

DaphniaROR GTATTGGAAAACGACATCTGCCGGATGGAATATGCC---------GTCGCCAAGCAACAT

DpulexTrk GCTGCAGGGAAACCTTCTCCCAAGGTTGCATGGAACACTAGCCGACTCGCTTCCAAGGTT

HumanTrkB GTGGCAGGTGATCCGGTTCCTAATATGTATTGGGAT---------GTTGGTAACCTGGTT

DrosophilaROR CTTCTGGAGAACGAGCTGTGCCAGAAGGAATATGCC---------ATTGCCAAGCGACAT

HumanROR1 ATCCTGGAGAATGTCCTGTGTCAAACAGAGTACATT---------TTTGCAAGATCAAAT

HumanTrkA GTGGAGGGGCGGGGCCTGGAGCAGGCCGGCTGGATC---------CTCACAGAGCTGGAG

HumanTrkC GGCTCTGGATCACCCCTTCCTGATGTGGACTGGATAGTCACTGGGCTGCAGTCCATCAAC

AplysiaTrkL AAATCCGGCATGCGA---TGTTATTGTGTCAACGAC---------CTCAATGAACCCGAC

DaphniaNRK GCGACGAGGCAGCTCTTTTGCGTCGACGACTGGGCC---------CAGCTGGAAAGCAAC

LymneaTrk ---------------ATTCGAAACATGACTTTGGTC---------TTTGAGCCGAAGAAT

AmphioxusROR ATCCTGGAGAACGACATCTGCAGGACGGAGTACATC---------CTGGCCAAGACGCAC

DrosophilaNRK GCCACGCATCTCCAGTTCTGCTACAACGACTGGGTG---------CTCATCGAGGAGAAG

AplysiaROR GCCCTGGAGAATGACATCTGCAGGACGGAGTACTTG---------ATGGCCAAACGACAT

DaphniaTrkL GGCTCATGGATTTGCATCTGCACTCACGACTTGTCA---------CTTCAAAGGCCCGAC

AmphioxusTrk TCCAAGTTCAGCCTTGTGATAGAGGAGCTGGGCAGACGGCAGGTCCTCACCATACACAAC

DaphniaROR CCG------------------------------------------CTTATCGGAGAACAG

DpulexTrk AAT---------------ATCGTCGGCTCTGGATTGCAACAGGCGTTGACTATCGAAAAC

HumanTrkB TCC------AAACATATGAATGAAACAAGCCACACACAGGGCTCCTTAAGGATAACTAAC

DrosophilaROR CCC------------------------------------------GTCATCGGGATGGTG

HumanROR1 CCC------------------------------------------ATGATTCTGATGAGG

HumanTrkA CAGTCAGCCACGGTGATGAAATCTGGGGGTCTGCCATCCCTGGGGCTGACCCTGGCCAAT

HumanTrkC ACTCACCAGACCAATCTGAACTGGACCAATGTTCATGCCATCAACTTGACGCTGGTGAAT

AplysiaTrkL CCC------------------------------------------GAAACCGGATCATGT

DaphniaNRK AAA------------------------------------------CAGCGCGGCTTCTAC

LymneaTrk GGT------------------------------------------ATGTTCCTTTTGCGT

AmphioxusROR CAC------------------------------------------CTCATCGGCGAGTGG

DrosophilaNRK AAG------------------------------------------GAGCGAAATATGTTC

AplysiaROR AAC------------------------------------------TTGATAGGCGACAAC

DaphniaTrkL GGA---------------------------------------------------------

AmphioxusTrk GCCAGTGAGGACGACAACGGC---AACTGGACCTGCATTGCCAAGAACATGGTTGGAATA

DaphniaROR GTCATG------------------CTTCCTGATTGTGAAAAGCTGCCGCCAATAGGATCC

DpulexTrk GTACGAGGTGACGATATGGGT---AGTATTACCTGCTCGGCGGAAAATGCAGTCGGAAAA

HumanTrkB ATTTCATCCGATGACAGTGGGAAGCAGATCTCTTGTGTGGCGGAAAATCTTGTAGGAGAA

DrosophilaROR GGT---------------------GTGGAGGATTGCCAAAAGTTGCCG------------

HumanROR1 CTGAAA------------------CTGCCAAACTGTGAAGATCTCCCCCAGCCAGAGAGC

HumanTrkA GTCACCAGTGACCTCAACAGGAAGAACGTGACGTGCTGGGCAGAGAACGATGTGGGCCGG

HumanTrkC GTGACGAGTGAGGACAATGGCTTCACCCTGACGTGCATTGCAGAGAACGTGGTGGGCATG

AplysiaTrkL ------------------------GCTCCAGGCATCACTGAAAAGCCGCGGGTGGGTGAC

DaphniaNRK TTGGGATCGCGTGGGCATTTCCGGTTGCCCAAATGCGAGAAGCTGCCCAGGATCGGCTCA

LymneaTrk TTCGTC------------------ATCTCTGGCTGTCCAAAA---CCTAAGATAGACTTA

AmphioxusROR ATC---------------------CTTCCCGACTGCTCCGAGCTGCCGCTCATCGGGACT

DrosophilaNRK ATCAAGAGCCGCGGCCACTTCCGGCTACCCAACTGCTCCTCCTTGCCGCACTACAACGCT

AplysiaROR CTA---------------------TTGCCGAAGTGCAGCCAACTTCAAGGGCCGGGAACT

DaphniaTrkL ------------------------------TTTTGCCCTCGCGTTGTCGTTACTGGACCT

AmphioxusTrk ---GCC------------------------------------------CGGGCAAACTTG

DaphniaROR ---AAG------------------------------------------GGCAGTGAGACG

DpulexTrk GTATCG------------------------------------------CGTAACTTTTCT

HumanTrkB ---GAT------------------------------------------CAAGATTCTGTC

DrosophilaROR ------------------------------------------------CAGCACAAGGAC

HumanROR1 ---CCA------------------------------------------GAAGCTGCGAAC

HumanTrkA ---GCA------------------------------------------GAGGTCTCTGTT

HumanTrkC ---AGC------------------------------------------AATGCCAGTGTT

AplysiaTrkL ---CCT------------------------------------------GAGCACGGAGTG

DaphniaNRK AGGAGCTTTTTCATGTCCAGCAGCCACAGCGGCAGCAAGTACGGCCAGGATCCGCCGAGT

LymneaTrk CTTCGA------------------------------------------AATCATCATCAT

AmphioxusROR ---CCC------------------------------------------GAGTCAGCCAAC

DrosophilaNRK TCCATG------------------------------------------CGGCGACCCAAC

AplysiaROR ---CGG------------------------------------------GAGGGGGACAAC

DaphniaTrkL ------------------------------------------------CATCATCCGAGC

AmphioxusTrk GTGTTGAAGATCAACGCCCCTCCTCGTATCCTGAGTCTGGAGTATAAG---GTCCTCTTC

DaphniaROR TGTTTCCGTATTGGAATTCCTCACATGGCTCAAATTGTGGAAGAACAA------------

DpulexTrk CTAATCGTGAATTCGGCACCCATTATTGTCAAATTTGAGATCCGCCAA------GGTTTC

HumanTrkB AACCTCACTGTGCATTTTGCACCAACTATCACATTTCTCGAATCTCCAACCTCAGACCAC

DrosophilaROR TGCCTATCCTTGGGCATCACC------ATCGAGGTGGATAAGACGGAG------------

HumanROR1 TGTATCCGGATTGGAATTCCCATGGCAGATCCTATAAATAAAAATCAC------------

HumanTrkA CAGGTCAACGTCTCCTTCCCGGCCAGTGTGCAGCTGCACACGGCGGTG---GAGATGCAC

HumanTrkC GCCCTCACTGTCTACTATCCCCCACGTGTGGTGAGCCTGGAGGAGCCTGAGCTGCGCCTG

AplysiaTrkL GTGTACAAC---------------------------------------------------

DaphniaNRK TGCTCGCGAGCCCATTTGACTCAGCTCGTTCCAGCCGAAGTGACCACT------------

LymneaTrk GTGCTTAGAAGCGGCTCCTCTCAGTTCAAGTTGACCGATTTCAAATCA------------

AmphioxusROR TGCATCCGGATAGGGATTCCCACCATGAACCACATAAACCGCAGTCAC------------

DrosophilaNRK TGCTCCTACATCGGTCTCACCGAACTCAAGGAGTCCGAAGTGAGCTAC------------

AplysiaROR TGTATCCGTATTGGGATGCCCCCTGGCTCCACGTCCGGACGCGGCCGCCCCAAAGGGGGC

DaphniaTrkL GCAAACAAAAAGACCTTCATTAGCATCCGCAAA---------------------------

AmphioxusTrk CACAAGAGC---------------------------------------------------

DaphniaROR ------------------------------------------------------------

DpulexTrk TACTGGTGT---------------------------------------------------

HumanTrkB CACTGGTGC---------------------------------------------------

DrosophilaROR ------------------------------------------------------------

HumanROR1 ------------------------------------------------------------

HumanTrkA CACTGGTGC---------------------------------------------------

HumanTrkC GAGCACTGC---------------------------------------------------

AplysiaTrkL ------------------------------------------------------------

DaphniaNRK ------------------------------------------------------------

LymneaTrk ------------------------------------------------------------

AmphioxusROR ------------------------------------------------------------

DrosophilaNRK ------------------------------------------------------------

AplysiaROR AACCCGAGCTGGAACAATCCGGGAACCCGCCGTGACCCTCCCAGGGGGTCAAAGGGCAGC

DaphniaTrkL ------------------------------------------------------------

AmphioxusTrk ------------------------------------------------------------

DaphniaROR ------------------------------------------------------------

DpulexTrk ------------------------------------------------------------

HumanTrkB ------------------------------------------------------------

DrosophilaROR ------------------------------------------------------------

HumanROR1 ------------------------------------------------------------

HumanTrkA ------------------------------------------------------------

HumanTrkC ------------------------------------------------------------

AplysiaTrkL ------------------------------------------------------------

DaphniaNRK ------------------------------------------------------------

LymneaTrk ------------------------------------------------------------

AmphioxusROR ------------------------------------------------------------

DrosophilaNRK ------------------------------------------------------------

AplysiaROR GGAAGTAAACGACCGACGTCTGATAAGGATACTGGTCGCGGCCAGCAAGGACCAACAGAT

DaphniaTrkL ------------------------------------------------------------

AmphioxusTrk ---ATCTGCTTCACCGTGCACGGGTATCCTCTGCCAACCCTCACCTGGTACAAGGGTCGC

DaphniaROR ---AGCTGTTACAATGAAGATGGC------------------CGTGATTACCGCGGAATT

DpulexTrk ---ATCGATTACGAAATTAAAGGATATCCAACACCCAATCGAACGTGGTTCTTCAACGGG

HumanTrkB ---ATTCCATTCACTGTGAAAGGCAACCCAAAACCAGCGCTTCAGTGGTTCTATAACGGG

DrosophilaROR ---AATTGTTACTGGGAGGATGGA------------------TCGACATATAGAGGAGTG

HumanROR1 ---AAGTGTTATAACAGCACAGGT------------------GTGGACTACCGGGGGACC

HumanTrkA ---ATCCCCTTCTCTGTGGATGGGCAGCCGGCACCGTCTCTGCGCTGGCTCTTCAATGGC

HumanTrkC ---ATCGAGTTTGTGGTGCGTGGCAACCCCCCACCAACGCTGCACTGGCTGCACAATGGG

AplysiaTrkL ------------------------------------------------------------

DaphniaNRK ---TCGTGCATCAAAGGACGCGGC------------------CGCTTCTACCAAGGAAAC

LymneaTrk ------------GAATTCAATGGC------------------CAAGTGGTAACGGGTACG

AmphioxusROR ---ACTTGTTACGAGGGCAAGGGC------------------AGCGGTTACCGGGGAACG

DrosophilaNRK ---GATTGCCGCAATGGAAACGGA------------------CGCTTCTACATGGGCACA

AplysiaROR GTGTACTGCTACACCGGAAGAGGC------------------ACGAACTACCGTGGTGAG

DaphniaTrkL ------------------------------------------------------------

AmphioxusTrk GCGCTGTTCACGGCCAGGAACCACATGTACGTCAGCATGGAGAATTCGAAGGTCACGCCG

DaphniaROR GCGAGCCGCACGACCAGTAAGCAAGACTGCCTGCCATGGAACCGCCAAGCGGCTGTGAAA

DpulexTrk GCGAAGCTCCTTTACTCCAACGATATTACAGACGTGTTCCACATTCCGAGCGAAAGGCTA

HumanTrkB GCAATATTGAATGAGTCCAAATACATCTGTACTAAAATACATGTTACCAATCACACGGAG

DrosophilaROR GCCAACGTCTCCGCATCCGGAAAGCCATGTTTGCGATGGTCATGGCTGATGAAGGAAATC

HumanROR1 GTCAGTGTGACCAAATCAGGGCGCCAGTGCCAGCCATGGAATTCCCAGTATCCCCACACA

HumanTrkA TCCGTGCTCAATGAGACCAGCTTCATCTTCACTGAGTTCCTGGAGCCGGCAGCCAATGAG

HumanTrkC CAGCCTCTGCGGGAGTCCAAGATCATCCATGTGGAATACTACCAAGAGGGAGAGATTTCC

AplysiaTrkL ------------------------------------------------------------

DaphniaNRK GTGAGCGTGACTCGAGATGGGATTGCATGCCAGCGCTGGGATGCCCAGGAGCCGCACTCG

LymneaTrk ATTACCATTTTACCC---------------------------------------------

AmphioxusROR GTTGCCGTGACAAAGTCGGGGATTCCGTGCCAGGCGTGGAACAAGGAGACCCCCCACGTC

DrosophilaNRK ATGAACGTGTCCAAGTCGGGCATTCCCTGCCAGCGCTGGGACACTCAGTACCCGCACAAG

AplysiaROR GTCAGTGTGTCAAAGTCTGGGTTCATGTGCTTGGGCTGGAAGGACTCCGGCTTC------

DaphniaTrkL ------------------------------------------------------------

AmphioxusTrk TACGAGACCAGCGGCTGTCTGAAGTTCGAGGTGCCCTCTCACGTGGACAATGGGAACTAC

DaphniaROR TCTGCCGACCAT---------------------TCTGAGCTAATCGGTGGCCACAATTAT

DpulexTrk ---------AAAGGTTCTCTGGAATTTACAAGCACTAGTCTCAATCGACAAGGGCACTAC

HumanTrkB TAC------CACGGCTGCCTCCAGCTGGATAATCCCACTCACATGAACAATGGGGACTAC

DrosophilaROR TCCGATTTC------------------------CCTGAACTCATCGGTCAG---AATTAT

HumanROR1 CACACTTTCACCGCCCTTCGTTTC---------CCAGAGCTGAATGGAGGCCATTCCTAC

HumanTrkA ACCGTGCGGCACGGGTGTCTGCGCCTCAACCAGCCCACCCACGTCAACAACGGCAACTAC

HumanTrkC ---------GAGGGCTGCCTGCTCTTCAACAAGCCCACCCACTACAACAATGGCAACTAT

AplysiaTrkL ---------------------------------CCCAGCTTACAGGACATCCTCAAACAC

DaphniaNRK CACAACCGTCCGCCACTCAACATTTTC------CCTGAAATGCAGGGCGCCGAGAATTTC

LymneaTrk ---------------------------------CACATGGAGACGTCACAGACAACTTAC

AmphioxusROR CACTTCCTGCGGGCCTCGCAGTTC---------CCCGAGCTCGCCGGCGGCCACAACTAC

DrosophilaNRK CACTTCCAGCCACCACTGGTCTTC---------CATCAGCTCCTGGAGGGCGAAAACTAC

AplysiaROR ---------------------------------CCTGAATTA---GGTGATCACAACTAC

DaphniaTrkL ---------------------------------CCGTCAGCGGCCGGAGTC---------

AmphioxusTrk ACTCTGGTGGCGACCAACACATATGGACAGACCAGCAGGTCACTCGTGGTCACCTTCATG

DaphniaROR ---------TGCCGTAATCCCGGTGGTGTCGAGGTCCAACCTTGG---------------

DpulexTrk ACTTTATCCGTTTCCAATGTTTACGGAACAGCCAATCAAACTCTGAAAGTCAACTTTCAT

HumanTrkB ACTCTAATAGCCAAGAATGAGTATGGGAAGGATGAGAAACAGATTTCTGCTCACTTCATG

DrosophilaROR ---------TGCAGAAATCCTGGAAGCGTTGAAAATAGTCCTTGG---------------

HumanROR1 ---------TGCCGCAACCCAGGGAATCAAAAGGAAGCTCCCTGG---------------

HumanTrkA ACGCTGCTGGCTGCCAACCCCTTCGGCCAGGCCTCCGCCTCCATCATGGCTGCCTTCATG

HumanTrkC ACCCTCATTGCCAAAAACCCACTGGGCACAGCCAACCAGACCATCAATGGCCACTTCCTC

AplysiaTrkL ------------------------------------------------------------

DaphniaNRK ---------TGCCGGAACGCCGGCGGAGAAGAGCCCCGTCCTTGG---------------

LymneaTrk GTTCTAACAGCCGTCAACTCGAAAGGCCAAGCGAATCAAACGTTCCATCTGTATGATCAA

AmphioxusROR ---------TGCCGTAACCCCGGCAACGAGATGGACGCGCCCTTC---------------

DrosophilaNRK ---------TGCCGGAATGCTGGCGGTGAGGAGCCGCATCCCTGG---------------

AplysiaROR ---------TGYCGTAACCCTAACGGGCGGGAAGACGCCCCCTGG---------------

DaphniaTrkL ---------AACCAGACGGCCCAGCAGAGCAAACAGCAGTCACTC---------------

AmphioxusTrk AAACTCCCCCCAGTCAGGACAACAAACTACACCATCCGTGCACCGCCAAACTCATCGTCC

DaphniaROR ------TGTTTCGTTGCCGGAGTGGAAGGACCAAACTCC---CGTCCACGCAGAGAATTT

DpulexTrk CAACAGTTTGCCGACTTTCACCCGGGAATCCCCGGATTAGTCCCACCAATGCAGCTACCC

HumanTrkB GGCTGGCCTGGAATTGACGATGGTGCAAACCCAAATTATCCTGATGTAATTTATGAAGAT

DrosophilaROR ------TGTTTTGTGGACTCCTCACGT---------------GAACGCATAATCGAACTT

HumanROR1 ------TGCTTCACCTTGGATGAA------------------AACTTTAAGTCTGATCTG

HumanTrkA GACAACCCTTTCGAGTTCAACCCCGAGGACCCCATC------CCTGTCTCCTTCTCGCCG

HumanTrkC AAGGAGCCCTTTCCAGAGAGCACGGATAACTTTATCTTGTTTGACGAAGTGAGTCCCACA

AplysiaTrkL ------TCAGAGACGAATGAGACTTCCTCCATGTTGCGTCAGCCCGCCCACTCTGATGAC

DaphniaNRK ------TGTTACACCACCGATCCT------------------TTGGTCCGGTGGCAGCCC

LymneaTrk ACGACTCCAGCTTCAAGTATTCATATCCCGTTGTCAAATATTCCACCACGCATTTCTTCT

AmphioxusROR ------TGCTTCACCACGGACGAG------------------GGGACGCGAGCAGAGGAG

DrosophilaNRK ------TGCTACACTGTGGATGAA------------------TCAGTGCGCTGGCAGCAC

AplysiaROR ------TGTTTCACCAATGACCGG------------------AAGATGCCCAAGGAGCTG

DaphniaTrkL ---------------------------------------ATCGATCCATCCAGTCATCCC

AmphioxusTrk CACGGAACA------CCC---------------------------------GGGCCGACC

DaphniaROR TGTTCTTTG------CCC------------AAATGC------------------------

DpulexTrk CCTCACTTGCCACTACCATCGTCAATCGATCGTTCACATTCAAACGCCAAGCCCAAAGAA

HumanTrkB TATGGAACTGCAGCGAAT------------GACATCGGGGACACCACGAACAGAAGTAAT

DrosophilaROR TGTGATATT------CCA------------AAATGT------------------------

HumanROR1 TGTGACATC------CCA------------GCTTGC------------------------

HumanTrkA GTGGACACT------------------------------------------AACAGCACA

HumanTrkC CCTCCTATC---------------------------------------------------

AplysiaTrkL GTGGCCACG------CCC------------ATTACC------------------------

DaphniaNRK TGTGACATC------CCG------------CAATGC---------------GGTCATTCA

LymneaTrk GCAACAACT------CCC------------AGAGCC------------------------

AmphioxusROR TGCGACATC------CCC------------AAGTGT------------------------

DrosophilaNRK TGCGATATA------CCC------------ATGTGTCCGGATTATGTGGACCCCAATGCT

AplysiaROR TGCGCAGTG------CCC------------AAGTGT------------------AGCGAC

DaphniaTrkL AATTCGATT------CCC------------ACCTTG------------------CCGGGG

AmphioxusTrk ACAGATGATAACGTACCAGAAAACCAGACTACAACATACACACGAATCGAGACCTACATC

DaphniaROR ---------------------------TCTATGGGTTACGATATCAACTGGCTGTACATA

DpulexTrk CCGCCGCCGTCATCAGCGGGAGAAATGGAAGAAAATCATTTTCTTTCGGCTCAGATTGCC

HumanTrkB GAAATCCCTTCCACAGACGTCACTGATAAAACCGGTCGGGAACATCTCTCGGTCTATGCT

DrosophilaROR ---------------------------------GCGGACAAAATATGGATTGCCATTGTC

HumanROR1 ---------GATTCAAAGGATTCCAAGGAGAAGAATAAAATGGAAATCCTGTACATACTA

HumanTrkA TCTGGAGACCCGGTGGAGAAGAAGGACGAAACACCTTTTGGGGTCTCGGTGGCTGTGGGC

HumanTrkC ------ACTGTGACCCACAAACCAGAAGAAGACACTTTTGGGGTATCCATAGCAGTTGGA

AplysiaTrkL ---------------------------------------------------CACATTGTG

DaphniaNRK GAGGTTGAAGTGACTCTCCTTCCGATGGAAGCCACTTTTACGCCGGGATTCGTTCTCCTT

LymneaTrk ---------------------AGTCCCACTGAGGACTTTGGTCCACAGACGCAGGTCATA

AmphioxusROR ---------GAAATGCCGGAGGGCGGGACCGACCAGATCATGAGCCTGATCATCATCCTG

DrosophilaNRK GTCGATTTGAACACGCCCATCAAGATGGAGAAGTTCTTCACGCCATCGATGATCTTTCTC

AplysiaROR TACGACGAGGGTCACCCAAGCGAGGCTGACGAAGGCTCCAACAAGCTGATGTACATCCTC

DaphniaTrkL TCTCTTCCGGCTCTGGACGGACAGCTGAACGCTTCGGCGACGGCGGCCATCACTTGGCTG

AmphioxusTrk GCCGTAGCCGTGTCATCTGTAGTGTTCATCGTCGTGGCCATCGTGCTGTACTGCATGAGG

DaphniaROR ATTATTCCTGCCGCCGCCGTCGGATTACTTGTGATTGTCATTCTCGTCGTCTGCTGCGTC

DpulexTrk ATGGCAACCATCGCTTTGGTGGTAGGCCTGACCGTGACTGGATTCCTAATCGTCTGGGCT

HumanTrkB GTGGTGGTGATTGCGTCTGTGGTGGGATTTTGCCTTTTGGTAATGCTGTTTCTGCTTAAG

DrosophilaROR GGAACGACTGCAGCCATTATTCTAATATTCATAATTATATTTGCGATAATACTTTTCAAA

HumanROR1 GTGCCAAGTGTGGCCATTCCCCTGGCCATTGCTTTACTCTTCTTCTTCATTTGCGTCTGT

HumanTrkA CTGGCCGTCTTTGCCTGC---CTCTTCCTTTCTACGCTGCTCCTTGTGCTCAACAAATGT

HumanTrkC CTTGCTGCTTTTGCCTGTGTCCTGTTGGTGGTTCTCTTCGTCATGATCAACAAATATGGT

AplysiaTrkL GCACCTGTGATTGGCTCCGTTCTCGTGTGTGTCATCCTCGTCGTTGTCGCTGTCCTTTGC

DaphniaNRK TTGGCCGGCGTCGGTTTCCTTTCGTTACTCATCTTCCTCCTGTTTCTACTGCTCTGCCAG

LymneaTrk CTGCCAGTTGTAGGCGTTGTCATCCTCCTGATCTCCGCCGTGTTCATCATTTACCTGTGT

AmphioxusROR GTGCCCAGCATCTCCGTACCTCTCATCATCGCCCTCATCTTCTTCATCTACTGCACCTGC

DrosophilaNRK TTGGCTGGAATAGGTTTCGTGGCCATTGTGACCCTGCACTTGATGATATTGCTAGTCTAT

AplysiaROR ATCCCGTCCTTGACCGTGCCGCTGGCTCTGGGTATTCTGCTGGCGCTGATCTGCTTCTGT

DaphniaTrkL GTCCCGGCGGTGGGAATCGTCACGATCCTTTCGATCCTGTTTCTGCTGAGGCGTCGGATG

AmphioxusTrk TCGCGAAAGATCAGCCGGAGGGAGTGCAACATCAACGGGTCGGCGGCGCCCCTGCAGCGG

DaphniaROR CGCCGATCGAAATGCAAAGCCAAAAACTCATCGGTAATGATCAAGGGACCCAATAGC---

DpulexTrk AAAGTTCGTCCGTGCAAGAAAATGACGTCCGACAATCGAAGCAGTCGCTACAGTAAAATG

HumanTrkB TTGGCAAGACACTCCAAGTTTGGCATGAAAGGCCCAGCCTCCGTTATCAGCAATGATGAT

DrosophilaROR AGGAGAACAATCATGCACTATGGAATGAGGAATATTCATAATATCAACACACCCAGCGCC

HumanROR1 CGGAAT------AACCAGAAGTCATCGTCGGCACCAGTCCAGAGGCAACCA---------

HumanTrkA GGACGGAGA---AACAAGTTTGGGATCAACCGCCCGGCTGTGCTGGCTCCAGAGGATGGG

HumanTrkC CGACGG------TCCAAATTTGGAATGAAGGGTCCCGTGGCTGTCATCAGTGGTGAGGAG

AplysiaTrkL CGTCGGCGACAAGTGAGGAAAAAACTCTGCTCCAAGCGGAACGGCGATAACATTGTCAGA

DaphniaNRK AGGGCCTTCAAGCACAAACAAGGCTACCAGCTCCCCTCATTA------------------

LymneaTrk CAAAGGGCGAAGCATCGGTCGCACGCCAGGCAGAGGTGCAAGAAGGCGCTGCTGGACAAG

AmphioxusROR CGTCGCCATGGGGACGCCCGCGGCAACGGAACACACGTGATGAAGGGCACGGCAAGC---

DrosophilaNRK AAGTTGTCCAAGCACAAG---GATTACTCTCAGCCTGCGGGAGCAGCCACTGCCGAATGC

AplysiaROR CAGAAGTCTCACAACACCAGAGCCTCCAGGCCTAACAACAAGCAGGCCCAGCCGGTA---

DaphniaTrkL CGCAACAAAAAGAAATCGCGACGGGCCGCCAGCTCGTCCGGCGTCGTAGAAAGCCGACAC

AmphioxusTrk TCCACCAGCGAGTCCAACGGGGAGGTGGTCTCCCGG------------------------

DaphniaROR ------------------------------------------------------------

DpulexTrk TTCTGCCTGCTTCACTTTTTCCACGGGCCTCGTAACGCGACAGAAGAAAACAGTCCAATG

HumanTrkB GACTCTGCCAGCCCACTCCATCACATCTCCAATGGGAGTAACACTCCATCTTCTTCGGAA

DrosophilaROR GATAAAAATATCTACGGAAATTCGCAGCTTAATAACGCACAA------------------

HumanROR1 ------------------------------------------------------------

HumanTrkA CTGGCCATGTCCCTGCATTTCATGACATTGGGTGGCAGCTCCCTGTCCCCCACCGAG---

HumanTrkC GACTCAGCCAGCCCACTGCACCACATCAACCACGGCATCACCACGCCCTCGTCACTGGAT

AplysiaTrkL AACACTGACGGC------------------------------------------------

DaphniaNRK ------------------------------------------------------------

LymneaTrk AAGTTCAACGAGTTTCAA------------------------------------------

AmphioxusROR ------------------------------------------------------------

DrosophilaNRK AGTGTTTCCATGCGTGGAGGAGGAGATTGTGGCGGCAATCTGAACACCAGTAGAGAAACC

AplysiaROR ------------------------------------------------------------

DaphniaTrkL CATCACCACCACCATCATTATCCATCGCCTGGCAAATTGACGGGCAATTCGCTTCTGCCG

AmphioxusTrk ---------------------------GACTCCCTGCGGCTCAGCAGACTTCATGTGGTG

DaphniaROR ---------------------------ATGGTTTCGACTTTGCAAGGACAGTCGACACCC

DpulexTrk TCTGCGACGGTGTTCCGAAGA------GACACGATCCCCATTCACGCG---ACCATGGCC

HumanTrkB GGTGGCCCAGATGCT------------GTCATTATTGGAATGACCAAGATCCCTGTCATT

DrosophilaROR ---------------------------GATGCTGGCAGGGGAAATCTGGGAAATCTATCC

HumanROR1 ---------------------------AAACACGTCAGAGGTCAAAATGTGGAGATGTCA

HumanTrkA ---------------------------GGCAAAGGCTCTGGGCTCCAAGGCCACATCATC

HumanTrkC GCCGGGCCCGACACT------------GTGGTCATTGGCATGACTCGCATCCCTGTCATT

AplysiaTrkL ---------------------------GCCCTGTTACTAGAGAGGATGAACAACATCAAC

DaphniaNRK ---------------------------CAAAAAGAATCGATCGACCTGCAAAAGCTGCGA

LymneaTrk ---------------------------GAGGGTGTGCCTCTGACGGGCCTGCAGCTGGTC

AmphioxusROR ---------------------------GAGACCATCCCGCTGCAGAACCTGTTCGCTAAA

DrosophilaNRK CTCGGAGGCAATGGA------------AACACGAACACCTTGGCAAAATGGGGCACCATC

AplysiaROR ---------------------------GAGATGAGTCCGCTCAACCCCAAGTCAGCTAGC

DaphniaTrkL GCCGCCGGGGAAATGCCCGTCTGTCTGACCAAGAACGTGTTGCACCAGGAGCGATTCACC

AmphioxusTrk GAGAATCCAAACTAC---------------------------------------TTCCGA

DaphniaROR AGCCGTATTCATGTC---------------------------------------------

DpulexTrk GAAAATCCTCACTACGAAGCCCATCACAGCTTCCCGTCCAATAACGATTCGAAAGAGGAT

HumanTrkB GAAAATCCCCAGTAC------------------------TTTGGCATCACCAACAGTCAG

DrosophilaROR GATCACGTTGCTTTG---------------------------AACTCCAAACTTATCGAA

HumanROR1 ATGCTGAATGCATAT---------------------------------------------

HumanTrkA GAGAACCCACAATAC---------------------------------------------

HumanTrkC GAGAACCCCCAGTAC------------------------TTCCGTCAGGGACACAACTGC

AplysiaTrkL AAGAACCCGACTTAC------------------------TTCAGTCCTTCCGGGGCTTCT

DaphniaNRK GACAACTCGACGTAC---------------------------CAGTGTGTCGGAGCCCGG

LymneaTrk GACAATCCAAACTAC---------------------------AATTTAACCAAGAAGAAA

AmphioxusROR CACGTGCCGCGCTGT---------------------------------------------

DrosophilaNRK AGGAGCACGGCCACAATACACAGCAATTGCGTGGCCCTTACTACGGTGACCAATGTGTCT

AplysiaROR AGGGCAAGGGAATTT---------------------------------------------

DaphniaTrkL AACAATCCCGAATACGACTGGAACGCGGATCGAGGATCGACGGGCAGCGGAGGCGATACG

AmphioxusTrk GACTCTAAGGAACTAGTCATC------------------------------CGCCATATC

DaphniaROR ---------------------------------------------------CCGGAGATC

DpulexTrk CGGGACCAGGTCTCCATGCCCCTGCTCGTCTTGGACAGGAAA---------CCGGAATAC

HumanTrkB CTCAAGCCAGACACATTTGTT------------------------------CAGCACATC

DrosophilaROR AGAAATACTCTGCTGAGGATA------------------------------AACCATTTT

HumanROR1 ---AAACCCAAGAGCAAGGCT------------------------------AAAGAGCTA

HumanTrkA ---TTCAGTGATGCCTGTGTT------------------------------CACCACATC

HumanTrkC CACAAGCCGGACACGTATGTG------------------------------CAGCACATT

AplysiaTrkL GCCCGACGGCACGCCGTG---------------------------------CAGGAAGTG

DaphniaNRK ATCGATCCATCGCTAGAGAAA------------------------------CTGGAATAT

LymneaTrk CACGTCGCCACCACGTGTCCG------------------------------AAAACGGTC

AmphioxusROR ---------------------------------------------------CCGCAGTTC

DrosophilaNRK GATGCGAAGGGCACGAAACCGAATGCACGCCTGGAGAAG------------TTGGAGTAC

AplysiaROR ------------------------------------------------------------

DaphniaTrkL AGCCAGCTCCTGCAGGCCAGCGGCAGCAGCGGCGGCCCAGGCGACCAGTTCAAACCATTT

AmphioxusTrk ---------CAGCGCGACACCATCCACTTCGTGGGCGAGCTCGGGGAGGGGGCCTTCGGA

DaphniaROR ---------TCTTTACACGCTGTGCGGTTCCAGCACGATCTCGGAGAAGGAGCTTTTGGC

DpulexTrk AAAGTCATTTCGTCGTCCTGTTTGCATTCGCTCAAAGACTTGGGAGAAGGTGTTTTCGGC

HumanTrkB ---------AAGCGACATAACATTGTTCTGAAAAGGGAGCTAGGCGAAGGAGCCTTTGGA

DrosophilaROR ---------ACGCTGCAGGATGTTGAGTTTCTGGAGGAGCTGGGCGAAGGAGCTTTTGGA

HumanROR1 ---------CCTCTTTCTGCTGTACGCTTTATGGAAGAATTGGGTGAGTGTGCCTTTGGA

HumanTrkA ---------AAGCGCCGGGACATCGTGCTCAAGTGGGAGCTGGGGGAGGGCGCCTTTGGG

HumanTrkC ---------AAGAGGAGAGACATCGTGCTGAAGCGAGAACTGGGTGAGGGAGCCTTTGGA

AplysiaTrkL ---------CCCGCAGACTGCCTCACTCTCATCGAGGTGGTGGGCGAGGGGGCGTTTGGA

DaphniaNRK ---------CCCCGCAACGACATTATCTACATCAGAGACATTGGCCAGGGAGCATTCGGC

LymneaTrk ---------AGGCTTCAGACCATACTGCTCATGCGCGTCATCGGGGAGGGAGCCTTTGGG

AmphioxusROR ---------CCGATCGGCGCGGTGCGCTTCCTCACCGAGCTGGGAGAGGGCGCTTTTGGC

DrosophilaNRK ---------CCACGCGGGGATATAGTGTATGTGAGATCATTGGGTCAAGGAGCCTTCGGT

AplysiaROR ---------CCCATGCCGAACATCCGCTTTCTTCAGGAACTTGGCGAAGGGGCTTTCGGG

DaphniaTrkL GTCATCATCCGGCCAGAATGGATCGAATTGAAACAGGAAATTGGAGAGGGCTGCTTTGGC

AmphioxusTrk AGAGTCTACCTCGGCAAA------TGCGAGAAG---------CTTAAACCCGACGAA---

DaphniaROR AAAGTAAGAAAAATCAAATTAATTTACCCAGGTCAGATTTACTTAAATCCCGGTGGT---

DpulexTrk AAAGTTCACCTGTCGACTTACTCCTCGTCGCAA------------GACCCGGAAGCC---

HumanTrkB AAAGTGTTCCTAGCTGAA------TGCTATAAC---------CTCTGTCCTGAGCAG---

DrosophilaROR AAAGTCTACAAGGGACAGCTC---------------------CTGCAGCCGAACAAA---

HumanROR1 AAAATCTATAAAGGCCATCTCTATCTCCCAGGC---------ATGGACCATGCTCAG---

HumanTrkA AAGGTCTTCCTTGCTGAG------TGCCACAAC---------CTCCTGCCTGAGCAG---

HumanTrkC AAGGTCTTCCTGGCCGAG------TGCTACAAC---------CTCAGCCCGACCAAG---

AplysiaTrkL CAGGTGTTCAGAGGAGAACTGCGCAGCGCAGAG------------------GGCGGG---

DaphniaNRK CGGGTGTTTCAAGCCAAA------GCTCCGAAT---------CTGGTCAAAGGTGAG---

LymneaTrk CGCGTGTTTCTGGGCACG------TGCGCCCAC---------CTGATCCAGAAGAAC---

AmphioxusROR AAGGTGTACAAAGGTGAGCTGGTGGTCACGGGG---------TCAGGCGAGGACGCC---

DrosophilaNRK CGCGTCTTCCAGGCCAGG------GCTCCTGGA---------CTTGTTCCCGATCAG---

AplysiaROR AAGGTCTACAAGGGGGAA------CTGGTCGGC---------TTGTACGGAGAGAGC---

DaphniaTrkL AAAGTCTTTCGTGGATCGCTGCGACGGCCACCA---------TCGGCGCCCAACGCACAA

AmphioxusTrk ---------------------------------------------GATGCCTCCCTGGTG

DaphniaROR ------------------------------------------------GTCATGCCCATT

DpulexTrk ---------------------------------------------GGAAAGTTTCTCGTC

HumanTrkB ---------------------------------------------GACAAGATCTTGGTG

DrosophilaROR ---------------------------------------------ACCACCATAACAGTT

HumanROR1 ------------------------------------------------------CTGGTT

HumanTrkA ---------------------------------------------GACAAGATGCTGGTG

HumanTrkC ---------------------------------------------GACAAGATGCTTGTG

AplysiaTrkL ---------------------------------------------GCTTGTCATCAAGTA

DaphniaNRK ---------------------------------------------GCCTGCTCCATTGTG

LymneaTrk ---------------------------------------------GAGTTTGCGATCGTC

AmphioxusROR ---------------------------------------------AAGAAGATCCTGGTG

DrosophilaNRK ---------------------------------------------GAAGATCTACTAGTC

AplysiaROR ---------------------------------------------TCTGTTACGACAGTG

DaphniaTrkL CCAGACGACGAACAAGAAGAAGAAGAAGATGAAGAAATTCACTTTGATGATGAAGCGGTG

AmphioxusTrk GCGGTGAAGACGCTGAAGGAGATGAGTGTCGAGGACGCGCGGAAGGACTTTGATCGGGAA

DaphniaROR GCCATCAAGACGCTGAAAGCAAACGCTTCGGTGAAAACCCAGCAAGATTTTCGCCGTGAA

DpulexTrk GCTGTGAAAATTCTCAAG---TTTTGCGGAGAAGAATCCGCCAAGGATTTCGACCGTGAA

HumanTrkB GCAGTGAAGACCCTGAAGGAT---GCCAGTGACAATGCACGCAAGGACTTCCACCGTGAG

DrosophilaROR GCCATCAAGGCGTTGAAGGAAAACGCCTCGGTGAAAACGCAGCAGGACTTTAAGCGCGAA

HumanROR1 GCTATCAAGACCTTGAAAGACTATAACAACCCCCAGCAATGGATGGAATTTCAACAAGAA

HumanTrkA GCTGTCAAGGCACTGAAGGAG---GCGTCCGAGAGTGCTCGGCAGGACTTCCAACGTGAG

HumanTrkC GCTGTGAAGGCCCTGAAGGAT---CCCACCCTGGCTGCCCGGAAGGATTTCCAGAGGGAG

AplysiaTrkL GCTGTCAAAGTACTCAAGGACGGGGCGTCGCCGGACGCCCATGAAGACTTCGAGAGGGAG

DaphniaNRK GCCGTCAAGACGCTGAAGGAGGAGGCCGACGATGAAATGTGCGCCAATTTCGAGAAGGAG

LymneaTrk GCCGTGAAGACGCTGAAAGGGAGCTGTAGCGATTCCTTGAAGAGAGACTTTGAAAGAGAA

AmphioxusROR GCCATCAAGACGCTGAAGGAGAACGCGACCTTGAAGACGCAGCACGATTTCCACCGCGAG

DrosophilaNRK GCTGTTAAGATGCTAAAGGACGACGCCAGCGACCAGATGCAGATGGATTTCGAGCGCGAG

AplysiaROR GCAATCAAGACGCTGAAGGAGAACGCGTTGCCCAAAGTGCAGAATGACTTTCGACGAGAG

DaphniaTrkL GCTGTCAAAGTTTTAAAAGCCGCAGCTGGACCAGCTGCCCAGGAGGATTTGCTCCAGGAA

AmphioxusTrk GCGGAGCTCCTGACCAACATGCAGCATGAGAACATTGTGAAGTTTTACGGGGTCTGCACG

DaphniaROR GTGGAGCTCATGTCTGAATTGCGTCATCCCAATATCGTATGTCTATTAGGAGTCGTCACA

DpulexTrk GCGTCGCTTTTGACTCAGCTGACTCACAAAAACATTGTCCAATTTCACGGAGCCTGCGTC

HumanTrkB GCCGAGCTCCTGACCAACCTCCAGCATGAGCACATCGTCAAGTTCTATGGCGTCTGCGTG

DrosophilaROR ATCGAACTAATCTCGGATCTAAAGCATCAGAATATAGTGTGCATATTGGGCGTAGTGCTC

HumanROR1 GCCTCCCTAATGGCAGAACTGCACCACCCCAATATTGTCTGCCTTCTAGGTGCCGTCACT

HumanTrkA GCTGAGCTGCTCACCATGCTGCAGCACCAGCACATCGTGCGCTTCTTCGGCGTCTGCACC

HumanTrkC GCCGAGCTGCTCACCAACCTGCAGCATGAGCACATTGTCAAGTTCTATGGAGTGTGCGGC

AplysiaTrkL GTTGAGATCATGTCAGCCTTTGACCATGACAACATTCTCAAGCTGCTGGGAATTGTGGTG

DaphniaNRK GCCTGCCTATTGGCCGAGCTGGACCATCCCAACATAATCGGCTTGCTGGGCGTCTGCGCC

LymneaTrk GCAGAGATGCTGGCCACGATAGAGCACGCCAACATTGTCACATTCTACGGCGTGTGTACG

AmphioxusROR GTCGACATGTTGGCTGACCTGCGTCACCAGAACATCGTGTGTCTGCTGGGGGTGGTGATG

DrosophilaNRK GCCTGTTTGCTGGCCGAGTTCGATCATCCCAATATCGTGAGGCTGCTGGGGGTGTGCGCC

AplysiaROR GTTGACCTGATGTCCGACATGCGTCACCCTAACATCGTATGCCTCCTGGGCGTGTGCATG

DaphniaTrkL GCTGAAATTATGGTTTCCTTCTCTCATCCAAACATCCTCTCGCTCAAAGGGATCGTCATC

AmphioxusTrk GAGGGA---------GAGCCCTGGCTGATGATCTTTGAGTACATGGAAAACGGGGACCTC

DaphniaROR CGGGAT---------CAACCGCAATGCATGCTGTTTGAATACATGGCGCAGGGAGATCTC

DpulexTrk GACGAA---------AAACCCTGGAAAATGGTCTTCGAGTACATGGAAAACGGAGATTTG

HumanTrkB GAGGGC---------GACCCCCTCATCATGGTCTTTGAGTACATGAAGCATGGGGACCTC

DrosophilaROR AATAAG---------GAGCCCTACTGCATGCTGTTCGAGTACATGGCCAATGGTGATCTG

HumanROR1 CAGGAA---------CAACCTGTGTGCATGCTTTTTGAGTATATTAATCAGGGGGATCTC

HumanTrkA GAGGGC---------CGCCCCCTGCTCATGGTCTTTGAGTATATGCGGCACGGGGACCTC

HumanTrkC GATGGG---------GACCCCCTCATCATGGTCTTTGAATACATGAAGCATGGAGACCTG

AplysiaTrkL CAAGGTGTGGAAGGAGCTCCTTAC---ATGGTGTTTGAGTACATGGAACACGGAGATCTC

DaphniaNRK GTCGGC---------AAGCCCATGTGTCTCCTGCTCGAATTTATGGAGCTGGGCGACCTG

LymneaTrk GAGAGT---------GATCAATGGATGATGATCTTTGAGTTCATGGAGAACGGGGATCTC

AmphioxusROR CGGGAC---------CAGCCCATGTGCATGCTGTTCGAGTACATGCGCTACGGAGACCTA

DrosophilaNRK TTGGGC---------AGACCCATGTGCCTGCTCTTCGAGTACATGGCTCCTGGCGATCTA

AplysiaROR AAACAA---------GAACCCATGTGCATGTTGTTCGAGTACATGGCTCAAGGCGACTTG

DaphniaTrkL AACGAGCCCAATATTGGACCTTGG---CTCGTGTTCGAGTACATGGCGCTGGGAGATCTG

AmphioxusTrk AACAACTACCTGAGATCTCACGGTCCGGACGCGGCCTTCCTG------------------

DaphniaROR CATGAATTTTTGGTAGCTCACTCACCGGCCGGCGATGGTTCG------------------

DpulexTrk AACCAGTTTTTGAGAGTCCGGGGACCCGACGCCCACCTCCTGGAAGCCCGTCACGACCCA

HumanTrkB AACAAGTTCCTCAGGGCACACGGCCCTGATGCCGTGCTGATG------------------

DrosophilaROR CACGAATTCCTAATCTCAAACTCACCC---------------------------------

HumanROR1 CATGAGTTCCTCATCATGAGATCCCCACACTCTGATGTTGGC------------------

HumanTrkA AACCGCTTCCTCCGATCCCATGGACCTGATGCCAAGCTGCTG------------------

HumanTrkC AATAAGTTCCTCAGGGCCCATGGGCCAGATGCAATGATCCTT------------------

AplysiaTrkL TCCGAGCTGTTGAGGAGGAACGACCCGCAC------CTGAGG------------------

DaphniaNRK AGACAATACTTGCGCTCCTGCTGCCCGTCCAATTACATCGCC------------------

LymneaTrk AATAAGTACTTGAGGATGCACGGCCCAGACGCTGCGTTTCTG------------------

AmphioxusROR CACGAGTTCCTGGTAATGCGATCCCCGCACTCCGACGTCGGG------------------

DrosophilaNRK AGCGAGTTCTTGCGCGCCTGCTCCCCATATGCCACACACCAG------------------

AplysiaROR CACGAGTACTTGCTCTCCCACTCACCACACTCTGACGTCACG------------------

DaphniaTrkL GCCCAGCTTCTACGG------TCCGCCAATGGCAATCTATTT------------------

AmphioxusTrk ------------ATCAAGAACCCGGCCACTCAC------------------------AAG

DaphniaROR ------------GTCTCCGGAATTGGTGCTGGAAGTGACGATGGAACAGCG------AGT

DpulexTrk ACTTGTATCAATATTTCTGGTCGCCATACCGAAGACGGCGGCCCCATGGATCCTCCGCAG

HumanTrkB ------------GCTGAGGGCAACCCGCCC---------------------------ACG

DrosophilaROR ------------ACCGAAGGC------------------------------------AAG

HumanROR1 ------------TGCAGCAGTGATGAAGATGGGACTGTGAAA---------------TCC

HumanTrkA ------------GCTGGTGGGGAGGATGTGGCTCCA---------------------GGC

HumanTrkC ------------GTGGATGGACAGCCACGCCAGGCCAAG------------------GGT

AplysiaTrkL ------------AGTGCCGACTCAAAGACT---------------------------TTC

DaphniaNRK ------------ATGCCCGAGTCGTCGGCCGGCGGATCGTCTGGCGACATCAAAGACGTC

LymneaTrk ------------AAAGATAGAGATTCAATGGACTCAGATGAA---------------GGG

AmphioxusROR ------------GGCAGCTCGGATGACGCGGGGTCGCAC------------------TCG

DrosophilaNRK ------------GCGCCGACACAGGATCGT---------------------------CTG

AplysiaROR ------------GCGGCTGAAGACGACAGCGGTACCGGAGGAGGA------------CAC

DaphniaTrkL ------------GCCAAAACCAAAACTGCG---------------------------CAT

AmphioxusTrk GAGCTGAGCATCGTGGAGCTGCTGCAGATCTCTGTGCAGGTCGCCTCCGGTGTGGAGTAC

DaphniaROR ACGCTGGAGCAATCGGATTTCCTATACATTGCAATTCAGATCGCTGCCGGAATGGAGTAT

DpulexTrk AAGCTTAGCTTGCTTATTTTGCTCCAAATGGCCAGAGATATTTCACAAGGGATGGAATAT

HumanTrkB GAACTGACGCAGTCGCAGATGCTGCATATAGCCCAGCAGATCGCCGCGGGCATGGTCTAC

DrosophilaROR TCGCTGTCGCAGTTGGAATTCCTGCAAATAGCTCTACAAATCAGCGAAGGAATGCAGTAT

HumanROR1 AGCCTGGACCACGGAGATTTTCTGCACATTGCAATTCAGATTGCAGCTGGCATGGAATAC

HumanTrkA CCCCTGGGTCTGGGGCAGCTGCTGGCCGTGGCTAGCCAGGTCGCTGCGGGGATGGTGTAC

HumanTrkC GAGCTGGGGCTCTCCCAAATGCTCCACATTGCCAGTCAGATCGCCTCGGGTATGGTGTAC

AplysiaTrkL CGGCTGAACAAGTCTGACCTGGTGGAGATCAGCGTCCAAATTGCTACCGGAATGCGTTAC

DaphniaNRK AAACTCTCGGCCGCCGATTTGACGAGCATGGGCCGCCAAATCGCCGACGGAATGGTCTAC

LymneaTrk CAGCTTACACGGGAACAGCTCATGAAGATTGTTTTACAGATAGCCAGTGCCATGGAGTAT

AmphioxusROR TCGCTGGACCACACGGACTTTCTCTGCATCGCTAACCAGATCGCGGGCGGGATGGATTAT

DrosophilaNRK CAGTTGAACGAGCTACATCTGCTGCAGATGGCGGCCAACATTGCAGCGGGCATGCTGTAT

AplysiaROR ATTCTGGAATATTCCGAAATGTTACACGTGTCCACACAGGTGGCAGCTGGCATGGAATAC

DaphniaTrkL TGTTTGAATCAGGAGGATCTGCACTCGATCGCGGCGCAAATCGCAGACGGGATGGCCTAT

AmphioxusTrk ATGGCGTCGCAGCACTTCGTGCACCGGGACCTGGCCACACGCAACTGTCTGGTGGGGGAC

DaphniaROR CTAGCTAGCCATCACTACGTCCATCGTGACTTGGCAGCAAGGAACTGTTTGATTAGTGAC

DpulexTrk CTCGCGTCGATGCACTACGTCCACAGGGATTTGGCGACGCGCAATTGCTTAGTGGGTAAG

HumanTrkB CTGGCGTCCCAGCACTTCGTGCACCGCGATTTGGCCACCAGGAACTGCCTGGTCGGGGAG

DrosophilaROR CTGTCGGCCCATCATTACGTACATCGCGACTTGGCAGCTCGGAATTGCCTGGTAAACGAG

HumanROR1 CTGTCTAGTCACTTCTTTGTCCACAAGGACCTTGCAGCTCGCAATATTTTAATCGGAGAG

HumanTrkA CTGGCGGGTCTGCATTTTGTGCACCGGGACCTGGCCACACGCAACTGTCTAGTGGGCCAG

HumanTrkC CTGGCCTCCCAGCACTTTGTGCACCGAGACCTGGCCACCAGGAACTGCCTGGTTGGAGCG

AplysiaTrkL CTGGCAGCTCAGAGGTTTGTCCACCGAGACCTGGCCACCCGAAACTGCCTGGTCGGCACA

DaphniaNRK CTCTCGCAGAGGGGCTTCGTCCACCGCGACCTGGCCACCCGCAACTGCCTGGTCAGCTCC

LymneaTrk CTGGCGTTGCAACATTTCGTCCATCGAGACCTGGCGACCAGAAACTGTTTGGTAGGCTGT

AmphioxusROR CTCGCCTCCAAACACTTCTGCCACCGCGACCTGGCCGCTCGAAACTGCCTCGTCGGGGAC

DrosophilaNRK CTTTCGGAGAGAAAATTCGTCCACCGGGATTTGGCCACCAGGAATTGCCTGATCAACGAG

AplysiaROR CTCGCCAGTCACCATTTCGTTCACAGAGATCTGGCCGCGAGGAACATCCTCGTGGCTGAC

DaphniaTrkL CTCTCGTCGCAGCATTTCGTCCATCGCGACTTGGCGTGCCGCAATTGTCTGGTGGGTGAA

AmphioxusTrk AAA------------------------CTCGTGGTGAAGATCGGAGACTTCGGGATGTCT

DaphniaROR AAC------------------------CTGATTGTGAAAATATCCGATTTTGGTCTGTCT

DpulexTrk AAT------------------------TTAGTGGTCAAAATAGGCGACTTTGGTATGTCC

HumanTrkB AAC------------------------TTGCTGGTGAAAATCGGGGACTTTGGGATGTCC

DrosophilaROR GGT------------------------CTGGTTGTGAAGATATCCGATTTTGGACTATCC

HumanROR1 CAA------------------------CTTCATGTAAAGATTTCAGACTTGGGGCTTTCC

HumanTrkA GGA------------------------CTGGTGGTCAAGATTGGTGATTTTGGCATGAGC

HumanTrkC AAT------------------------CTGCTAGTGAAGATTGGGGACTTCGGCATGTCC

AplysiaTrkL GCGCCACCT---------GGTGCCGGACTTCTGGTGAAGATTTCCGACTTTGGCATGTCT

DaphniaNRK AACGGGCCGCCCAGCAGCGGCGGAGGCGTCACCGTCAAGATCGCCGACTTTGGCCTGTCG

LymneaTrk GAT------------------------CTTGTGGTCAAACTCGGTGACTTTGGCATGTCC

AmphioxusROR AAC------------------------CTGCTCATCAAGATATCCGATTTCGGCCTTTCC

DrosophilaNRK CAC------------------------ATGGCGGTAAAGATCGCCGACTTTGGGCTCTCG

AplysiaROR GGG------------------------CTAACTGTGAAGATCTCCGACTTTGGTTTGTCC

DaphniaTrkL CGACCCCGA---------GGCGGCGGTCTGGCCGTCAAAATCTCCGATTTCGGAATGAGC

AmphioxusTrk CGGGACATCTACAGCACCGACTACTATAGA------------------------------

DaphniaROR CGAGACATTTACTCGTCTGATTACTACCGT------------------------------

DpulexTrk AGGGATATTTACAGCTCCGACTACTATCGGGTAAGTGCAGGATCACACCCTGAAGAAAGA

HumanTrkB CGGGACGTGTACAGCACTGACTACTACAGG------------------------------

DrosophilaROR AGAGACATTTACAGCTCAGATTATTATCGA------------------------------

HumanROR1 AGAGAAATTTACTCCGCTGATTACTACAGG------------------------------

HumanTrkA AGGGATATCTACAGCACCGACTATTACCGT------------------------------

HumanTrkC AGAGATGTCTACAGCACGGATTATTACAGGCTC---------------------------

AplysiaTrkL AGGGATATCTACACTAACGACTACTACAAG------------------------------

DaphniaNRK CAGCGCGTCCACTGGCAGCAGTACTACTACACG---------------------------

LymneaTrk AGAGATGTGTACACTACGGACTACTACAGG------------------------------

AmphioxusROR CGCGATATCTACTCCTCGGATTACTACCGT------------------------------

DrosophilaNRK CACAAGATCTATTTGCAGGACTATTACAAA------------------------------

AplysiaROR AGAGATGTCTACTCTTCTGATTACTACAGA------------------------------

DaphniaTrkL CGTGACGTTTACACTTGCGATTATTACAAG------------------------------

AmphioxusTrk ------------------------------------------------GTTGGGGGACAC

DaphniaROR ------------------------------------------------GTCCAGGGCAAA

DpulexTrk ATGAGACAAATTACAGCTTGTCAAAGTGAAAAATTGATATTTTACAAGGTTGGTGGACAC

HumanTrkB ------------------------------------------------GTCGGTGGCCAC

DrosophilaROR ------------------------------------------------GTTCAGTCAAAG

HumanROR1 ------------------------------------------------GTCCAGAGTAAG

HumanTrkA ------------------------------------------------GTGGGAGGCCGC

HumanTrkC ---------TTTAATCCATCTGGAAATGATTTTTGTATATGGTGTGAGGTGGGAGGACAC

AplysiaTrkL ------------------------------------------------ATCGGCGGGTCT

DaphniaNRK ------------------------------------------------GGGACGGACAAC

LymneaTrk ------------------------------------------------GTTGAAGGCACG

AmphioxusROR ------------------------------------------------GTGCAGTCCAAG

DrosophilaNRK ------------------------------------------------GGCGATGAGAAC

AplysiaROR ------------------------------------------------GTGCAGAGCAAG

DaphniaTrkL ------------------------------------------------ATTGGAGGTTCT

AmphioxusTrk ACGATGCTGCCTGTACGGTGGATGCCACCAGAAAGCGTGCTCTACCGGAAATTCACCATC

DaphniaROR TCAATGTTGCCTGTTCGTTGGATGCCACCAGAAGCTATTCTATACGGAAAATTCACAATT

DpulexTrk ACTTTACTTCCAGTCCGGTGGATGCCTCCAGAGAGTGTTATGTACCGCAAATTCACGTCC

HumanTrkB ACAATGCTGCCCATTCGCTGGATGCCTCCAGAGAGCATCATGTACAGGAAATTCACGACG

DrosophilaROR TCGCTATTGCCTGTAAGGTGGATGCCCTCGGAATCGATATTGTATGGAAAGTTTACGACC

HumanROR1 TCCTTGCTGCCCATTCGCTGGATGCCCCCTGAAGCCATCATGTATGGCAAATTCTCTTCT

HumanTrkA ACCATGCTGCCCATTCGCTGGATGCCGCCCGAGAGCATCCTGTACCGTAAGTTCACCACC

HumanTrkC ACCATGCTCCCCATTCGCTGGATGCCTCCTGAAAGCATCATGTACCGGAAGTTCACTACA

AplysiaTrkL CGAATGCTTCCAATCCGCTGGATGTCACCGGAAGCCATCAAGTATGGCCGCTTCACATGT

DaphniaNRK GATGCCATACCCATCCGCTGGATGCCGCTGGAGAGCATCATCTTCAACCGCTACACCACG

LymneaTrk GCCATGCTGCCTGTAAGATGGATGCCGCCGGAGAGCATCATCTACAGGACGTTCACCACT

AmphioxusROR TCGCTCCTGCCGGTTCGCTGGATGCCGCCGGAGGCGATCATGTACGGGAAGTTTTCCACG

DrosophilaNRK GACTTCATCCCGATCCGCTGGATGCCACTTGAGAGCATACTGTACAACAAGTTCTCGCTT

AplysiaROR TCTTTGCTCCCCGTCAGATGGATGCCCCCGGAAGCGATCTTGTACGGAAAGTTCACCACA

DaphniaTrkL CGGATGCTGCCGGTGCGATGGATGGCTCCCGAATCGATCCTCTACGGTAAATTCACGCTA

AmphioxusTrk GAAAGTGACATCTGGAGTTTCGGCGTCGTGCTATGGGAAATCTTTACGTTTGGGAAACAG

DaphniaROR GAATCAGATGTTTGGAGCTTCGGCGTTGTCCTCTGGGAAATCTACAGTTTTGCATTACAG

DpulexTrk GAGTCGGACGTCTGGTCATTTGGAGTGGTGCTTTGGGAAATCTTCTCCTTTGGCAAACAG

HumanTrkB GAAAGCGACGTCTGGAGCCTGGGGGTCGTGTTGTGGGAGATTTTCACCTATGGCAAACAG

DrosophilaROR GAGAGCGATGTTTGGTCCTTTGGAGTCGTTCTTTGGGAAATATACAGCTATGGAATGCAG

HumanROR1 GATTCAGATATCTGGTCCTTTGGGGTTGTCTTGTGGGAGATTTTCAGTTTTGGACTCCAG

HumanTrkA GAGAGCGACGTGTGGAGCTTCGGCGTGGTGCTCTGGGAGATCTTCACCTACGGCAAGCAG

HumanTrkC GAGAGTGATGTATGGAGCTTCGGGGTGATCCTCTGGGAGATCTTCACCTATGGAAAGCAG

AplysiaTrkL GAGAGTGATGTGTGGGCCTATGGAGTTGTCTTATGGGAGATTTTCAGTTACGGCAGACAA

DaphniaNRK TCGTCGGATGTCTGGGCCTTTGGCGTCTGCCTCTGGGAGATCTTCTCCTACGCCCAGCAG

LymneaTrk GAATCAGACGTCTGGAGCTTTGGCGTAACGCTCTGGGAGGTCTTCACGTACGGCAAGCAG

AmphioxusROR GACAGCGATGTTTGGTCGTTCGGCGTGGTTCTGTGGGAGATCTTCAGCTACGGACTCCAG

DrosophilaNRK GAGTCGGATGTGTGGGCATACGGCATCTGTCTGTGGGAGGTCTTCTCCTTCGCCTTGCAG

AplysiaROR GACAGTGATGTGTGGGCTTTTGGCGTTGTCCTCTGGGAGGTCTTCAGCTATGGACTGCAG

DaphniaTrkL GAATCGGATGTCTGGAGTTTCGGCGTCGTCCTGTGGGAGGTCTTTGCCCTAGGGGTACAG

AmphioxusTrk CCGTGGTACGAGCTGGCCAATCACGAGGTGATCGAGTGCATCACCAGCGGCAGACTGTTG

DaphniaROR CCGTATTATGGATACAATAACCAAGATGTCATCGACATGGTGCGTTCTAGACAGCTACTC

DpulexTrk CCTTGGTACGGCTATTCCAATCAAGAGGTCATACAACTAGTCACAGGAGGCCAAGTACTT

HumanTrkB CCCTGGTACCAGCTGTCAAACAATGAGGTGATAGAGTGTATCACTCAGGGCCGAGTCCTG

DrosophilaROR CCATACTACGGTTTTAGCAATCAGGAAGTAATCAATCTCATCCGTTCACGGCAACTGCTC

HumanROR1 CCATATTATGGATTCAGTAACCAGGAAGTGATTGAGATGGTGAGAAAACGGCAGCTCTTA

HumanTrkA CCCTGGTACCAGCTCTCCAACACGGAGGCAATCGACTGCATCACGCAGGGACGTGAGTTG

HumanTrkC CCATGGTTCCAACTCTCAAACACGGAGGTCATTGAGTGCATTACCCAAGGTCGTGTTTTG

AplysiaTrkL CCATATTTTGGACATTCGAATGAGGAGGTTATTCACTTTCTAGATCAAGGAATTCTCCTC

DaphniaNRK CCCTACCACGGAATGTCTCACGAGGAAGTCGTCCGCTACTTGCAGGCCGGCGGAATGCTC

LymneaTrk CCGTGGTTTGAGTACTCCAATAGTGAGGTCATAGAACACATCAAAAATAGCCGGACCCTC

AmphioxusROR CCGTACTACGGGTACAGCAACCAGGAGGTGATCGAGATGATCCGGTCGCGCCAGCTGCTG

DrosophilaNRK CCCTACTTTGGGTTAACCCACGAGGAGGTGATCAAATACATCAAGGAGGGCAACGTACTC

AplysiaROR CCGTATTACGGTTTCTCCAATCAAGAGGTCATTGAGATGATACGCTCCAGGCAGATTCTG

DaphniaTrkL CCCTACTACGGCCATTCAAACGAACAGGTCGTCAAACTGATCCTACAAGGGATCCTCCTG

AmphioxusTrk GGG---TGCCCGCGTGGCTGCCCACGGAACGTGCGGGCCCTCATGCTCGGCTGCTGGAAG

DaphniaROR TCT---TGCCCATCAGAATGCCCGTCACGAATTTATTCCTTGATGATCGAGTGCTGGTCT

DpulexTrk CCA---TGTCCTCTTGCCACTCCTCCCGATGCGTATCAATTGATGTTGAATTGCTGGCAA

HumanTrkB CAG---CGACCCCGCACGTGCCCCCAGGAGGTGTATGAGCTGATGCTGGGGTGCTGGCAG

DrosophilaROR TCC---GCTCCGGAAAACTGTCCCACTGCTGTCTACTCGCTAATGATCGAGTGCTGGCAT

HumanROR1 CCA---TGCTCTGAAGACTGCCCACCCAGAATGTACAGCCTCATGACAGAGTGCTGGAAT

HumanTrkA GAG---CGGCCACGTGCCTGCCCACCAGAGGTCTACGCCATCATGCGGGGCTGCTGGCAG

HumanTrkC GAG---CGGCCCCGAGTCTGCCCCAAAGAGGTGTACGATGTCATGCTGGGGTGCTGGCAG

AplysiaTrkL CAG---CGACCGGAAGACTGCCCCTCCACCGTCTATCACGTGATGCTTGGTTGCTGGAAG

DaphniaNRK CAG---CCGCCGGCCCACGCGTCCTGTGCCATTTACGCCGTCATGCGCTCCTGCTGGCAC

LymneaTrk AAGCGGCCTCCCAGGACCTGTACAGACGGCGTCTACAGAGTAATGCAGGGGTGTTGGAAG

AmphioxusROR CCG---TGCCCCGACAACTGCCCGGCCAGGATGTACTCCCTGATGCTGGAGTGCTGGAAC

DrosophilaNRK GGC---TGTCCGGACAACACGCCGCTCTCCGTCTACGCGCTGATGCGTCGCTGCTGGAAC

AplysiaROR GGC---TGTCCTGAAGAATGCCCGGCTCGTATTTACGGTCTGATGGTGGAGTGTTGGCAC

DaphniaTrkL ACT---CCTCCTTCTTCAGCCCCGCCCCTCATTTGCCAGTTGCTAAACGGTTGCTGGAAA

AmphioxusTrk AAAACCCCCGCCCAGCGCACCAACATC---------------------CAGGACATCCAC

DaphniaROR GAAGTTCCTCTGAGGAGGCCCACATTC---------------------ACAGAGGTTCAC

DpulexTrk ACACAACCCAATCAGCGGAGCACGATG---------------------AAAGCGGTCAAT

HumanTrkB CGAGAGCCCCACATGAGGAAGAACATC---------------------AAGGGCATCCAT

DrosophilaROR GAGCAGTCAGTAAAACGTCCAACATTC---------------------ACAGATATTTCG

HumanROR1 GAGATTCCTTCTAGGAGACCAAGATTT---------------------AAAGATATTCAC

HumanTrkA CGGGAGCCCCAGCAACGCCACAGCATC---------------------AAGGATGTGCAC

HumanTrkC AGGGAACCACAGCAGCGGTTGAACATC---------------------AAGGAGATCTAC

AplysiaTrkL GGTGACCCCAGGCAGAGAATCGTCTTTGACCGTCTCCTCAAGTACCTGACAGACTACCGA

DaphniaNRK AGCTCAGCCGGAGAGCGGCCCAGCTTC---------------------GTCGACCTGCAC

LymneaTrk CCCAACCCACAAGACAGACTGACTATG---------------------AAAGATATCGCG

AmphioxusROR GAGATCCCGGCGCGCAGACCGAGCTTC---------------------AACCAGATCCAC

DrosophilaNRK CGCAAGCCCAGTGAGCGACCTGGCTTC---------------------GCCGAGATCAAC

AplysiaROR GAGATGCCCGCCAGGCGTCCTCCTTTC---------------------CGGGAAATTCAC

DaphniaTrkL ACACAGCCGGGGGATCGGCTAACGTTC---------------------GCCGAAATCCAT

AmphioxusTrk AAGAAGCTCAAGGAAATG------------------------------------------

DaphniaROR AACAGATTGCGTTCGTGG------------------------------------------

DpulexTrk GAACGTTTAGTCGAGTTG------------------------------------------

HumanTrkB ACCCTCCTTCAGAACTTG------------------------------------------

DrosophilaROR AACCGTCTCAAAACTTGG------------------------------------------

HumanROR1 GTCCGGCTTCGGTCCTGG------------------------------------------

HumanTrkA GCCCGGCTGCAAGCCCTG------------------------------------------

HumanTrkC AAAATCCTCCATGCTTTG------------------------------------------

AplysiaTrkL GACCGGCTGAACAAACCG------------------------------------------

DaphniaNRK GACGAGCTTGTGGCTATC------------------------------------------

LymneaTrk GAGCTCCTCCGCGAAGAA------------------------------------------

AmphioxusROR ACCCGGTTACGGGCTTGG------------------------------------------

DrosophilaNRK CACTGCATCCAGCACAGC------------------------------------------

AplysiaROR ACTCGACTTCGCACCTGGCGTAGCGAGCTGACCACGTCCAACCCGTGGTCCCTCTCGCAG

DaphniaTrkL TCCAAGTTAAAGCGTCGC------------------------------------------

AmphioxusTrk ------------------------------------------------------------

DaphniaROR ------------------------------------------------------------

DpulexTrk ------------------------------------------------------------

HumanTrkB ------------------------------------------------------------

DrosophilaROR ------------------------------------------------------------

HumanROR1 ------------------------------GAGGGACTCTCAAGTCACACAAGCTCTACT

HumanTrkA ------------------------------------------------------------

HumanTrkC ------------------------------------------------------------

AplysiaTrkL ---------------------ACACATTGTCAACAGATGCCAATGTCAAGGCCTGTTGCT

DaphniaNRK ------------------------------------------------------------

LymneaTrk ------------------------------------------------------------

AmphioxusROR ---------------------GAGGGCATGGCGCACTCCGGGAGCCACAGCAGCGGGAGC

DrosophilaNRK ------------------------------------------------------------

AplysiaROR AGTCAGAGCGGTCAGTCTTCGTCCACCCACCAGAGCACGCAGTCGCAGCCGTCCCACCAC

DaphniaTrkL ------------------------------------------------------CCACTC

AmphioxusTrk ------------------------------------------------------------

DaphniaROR ------------------------GAAGGACTGGCATCAGTTGGAGCTCCCAGCAGTTCG

DpulexTrk ------------------------------------------------------------

HumanTrkB ------------------------------------------------------------

DrosophilaROR ------------------------------------------------------------

HumanROR1 ACTCCTTCAGGGGGAAATGCCACCACACAGACAACCTCCCTCAGTGCCAGCCCAGTGAGT

HumanTrkA ------------------------------------------------------------

HumanTrkC ------------------------------------------------------------

AplysiaTrkL GACTGCGCCCAGCTAGGAGACTCTCCCTCTGTCTCGCCGCGGACAGACGCCACTTGCAAA

DaphniaNRK ------------------------------------------------------GAAGAA

LymneaTrk ------------------------------------------------------------

AmphioxusROR AACCACTCCAACGCTAACGTGCAGGGGGCCCCCTCATCCGCTTCACAATCCCCAGTACAC

DrosophilaNRK ------------------------------------------------------------

AplysiaROR AGCAGCACGGGGCCCAGCAACACCACGGCCGTCACCGGACTGACCGGCAGCAGCAACACG

DaphniaTrkL TCAAACGACCATCAGGGAACTTGGACGACGCAATCGACAACCGTCACCTACGTCCATTTG

AmphioxusTrk ------------------TTGGAGAACCGCCACCAAGTTCAGGTTGACATTGTTGCC---

DaphniaROR ATTGGAGGACCACCACCCATCCCGGCTACTTTACCTCCCACCAATGGGGCTATGCTA---

DpulexTrk ---------TGTTCACCTTGTGTCAAAAATTCTACCCTCTATTTGAACGTAATAGAG---

HumanTrkB ---------------------GCCAAGGCATCTCCGGTCTACCTGGACATTCTAGGC---

DrosophilaROR ---------CACGAGGGCCACTTTAAGGCCAGTAATCCAGAAATG---------------

HumanROR1 AATCTCAGTAACCCCAGATATCCTAATTACATGTTCCCGAGCCAGGGTATTACACCACAG

HumanTrkA ---------------------GCCCAGGCACCTCCTGTCTACCTGGATGTCCTGGGC---

HumanTrkC ---------------------GGGAAGGCCACCCCAATCTACCTGGACATTCTTGGC---

AplysiaTrkL GGGGAAATAACTCGCGTCTCTATTGCCGACGTCAGTTGTGAGGCTGGCGACGACGTGACA

DaphniaNRK GCCCTTTGTTGCCAACCTCGTCCCGATACTGATCCACCTCCCGTTCACCTT---------

LymneaTrk ---------------------GTCTCCGGTGATCCAGTGTACATTGACATCATTGCA---

AmphioxusROR ACCCCCAAGCACTTCCCCAACCCCCCCTTCAACCCCCCCTCCTCCCAATCAGCCTTC---

DrosophilaNRK ---------------------ATCGCCGAGAGCGAGTGCAAGGCAATGCTC---------

AplysiaROR TCAGAGCCCAGCCCGGGCCAGCCCATGTACACGCCACACTACATGCCTTACAACAACCAC

DaphniaTrkL AGACATAACGAGGACAACAACGTCGATTCCAACGCCGAATACCTTCAAACGCTACCG---

AmphioxusTrk ------------------------------------------------------------

DaphniaROR ------------------------------------------------------------

DpulexTrk ------------------------------------------------------------

HumanTrkB ------------------------------------------------------------

DrosophilaROR ------------------------------------------------------------

HumanROR1 GGCCAGATTGCTGGTTTCATTGGCCCGCCA------------------------------

HumanTrkA ------------------------------------------------------------

HumanTrkC ------------------------------------------------------------

AplysiaTrkL TTTGTGGTGGACATGAACGGAGTAGAGAAAAAATTGACGTCGTATCGTGAACARAAACAT

DaphniaNRK ------------------------------------------------------------

LymneaTrk ------------------------------------------------------------

AmphioxusROR ------------------------------------------------------------

DrosophilaNRK ------------------------------------------------------------

AplysiaROR GGCGTGGCCGGGGGCTCGCTCTCGCCCCCGCCCTACAACGGAATACAACCACAGCAACCA

DaphniaTrkL ------------------------------------------------------------

AmphioxusTrk ------------------------------------------------------------

DaphniaROR ------------------------------------------------------------

DpulexTrk ------------------------------------------------------------

HumanTrkB ------------------------------------------------------------

DrosophilaROR ------------------------------------------------------------

HumanROR1 ------------------------------------------------------------

HumanTrkA ------------------------------------------------------------

HumanTrkC ------------------------------------------------------------

AplysiaTrkL TGTGGCGCTCCAAACTCAGAGTGGAACTCGGGGACCCTGAACGAGCCTCGTTCCAAGTCA

DaphniaNRK ------------------------------------------------------------

LymneaTrk ------------------------------------------------------------

AmphioxusROR ------------------------------------------------------------

DrosophilaNRK ------------------------------------------------------------

AplysiaROR CAACAGCACGGCATCACTGCCGCACCAACAGCTGTTACCACAGCGACTCCTGCTGGGAAC

DaphniaTrkL ------------------------------------------------------------

AmphioxusTrk ------------------------------------------------------------

DaphniaROR ------------------------------------------------------------

DpulexTrk ------------------------------------------------------------

HumanTrkB ------------------------------------------------------------

DrosophilaROR ------------------------------------------------------------

HumanROR1 ------------------------------------------------------------

HumanTrkA ------------------------------------------------------------

HumanTrkC ------------------------------------------------------------

AplysiaTrkL GCTGCAGCTAAAAGCTGCGCCGGAAGTTCGCTAGTTGCCACACCACCTATGAAGGAGCAC

DaphniaNRK ------------------------------------------------------------

LymneaTrk ------------------------------------------------------------

AmphioxusROR ------------------------------------------------------------

DrosophilaNRK ------------------------------------------------------------

AplysiaROR ATTCAGCAACAGCAGCAGCAGCAGCAACTGCAGCAACAACAACTGCAGCAACAGCAACTG

DaphniaTrkL ------------------------------------------------------------

AmphioxusTrk ------------------------------------------------------------

DaphniaROR ------------------------------------------------------------

DpulexTrk ------------------------------------------------------------

HumanTrkB ------------------------------------------------------------

DrosophilaROR ------------------------------------------------------------

HumanROR1 ------------------------------------------------------------

HumanTrkA ------------------------------------------------------------

HumanTrkC ------------------------------------------------------------

AplysiaTrkL AAGTTCCCTGGAAGTAGCAAAGGATCAAGTGGCAGATCTCGAGTCCCTCTAGAACCTACC

DaphniaNRK ------------------------------------------------------------

LymneaTrk ------------------------------------------------------------

AmphioxusROR ------------------------------------------------------------

DrosophilaNRK ------------------------------------------------------------

AplysiaROR CAGCAGCAATACAAAGTGAATCCATTTTTGGGTCAGATGGGAGGAGTAGGAGGTCAGTAC

DaphniaTrkL ------------------------------------------------------------

AmphioxusTrk ------------------------------------------------------------

DaphniaROR ------------------------------------------------------------

DpulexTrk ------------------------------------------------------------

HumanTrkB ------------------------------------------------------------

DrosophilaROR ------------------------------------------------------------

HumanROR1 ------------------------------------ATACCTCAGAACCAGCGATTCATT

HumanTrkA ------------------------------------------------------------

HumanTrkC ------------------------------------------------------------

AplysiaTrkL GTTCTCTCTCGACAAGGATCTCACGGCAGGATCGAGGCGAATCGCAACATGATTCCCTAT

DaphniaNRK ------------------------------------------------------------

LymneaTrk ------------------------------------------------------------

AmphioxusROR ------------------------------------------------------------

DrosophilaNRK ------------------------------------------------------------

AplysiaROR GTTCAGTACCCTGGTGGCCAGCCAGCTGTCTTGAATCTCCAGTCTGGCCAGGTTCAAATC

DaphniaTrkL ------------------------------------------------------------

AmphioxusTrk ------------------------------------------------------------

DaphniaROR ------------------------------------------------------------

DpulexTrk ------------------------------------------------------------

HumanTrkB ------------------------------------------------------------

DrosophilaROR ------------------------------------------------------------

HumanROR1 CCCATCAAT---GGATACCCAATACCTCCTGGATATGCAGCGTTTCCAGCTGCCCACTAC

HumanTrkA ------------------------------------------------------------

HumanTrkC ------------------------------------------------------------

AplysiaTrkL ACTGAGGCAATGACCTTTCACCTCCGCCGCTCCAGCAGCGCGTGCTACCTGAAGCATATC

DaphniaNRK ------------------------------------------------------------

LymneaTrk ------------------------------------------------------------

AmphioxusROR ------------------------------------------------------------

DrosophilaNRK ------------------------------------------------------------

AplysiaROR CCGCGCAACGTGGGACAAGCCATGCCCCCTGTCAAC------CCGACCACAGCAGGGCTG

DaphniaTrkL ------------------------------------------------------------

AmphioxusTrk ------------------------------------------------------------

DaphniaROR ------------------------------------------------------------

DpulexTrk ------------------------------------------------------------

HumanTrkB ------------------------------------------------------------

DrosophilaROR ------------------------------------------------------------

HumanROR1 CAGCCAACAGGTCCTCCCAGAGTGATTCAGCACTGCCCACCTCCCAAGAGTCGGTCCCCA

HumanTrkA ------------------------------------------------------------

HumanTrkC ------------------------------------------------------------

AplysiaTrkL ACAGACTCTGCCCTCAACGAACCTCGKGTGTCGTGTTCGTTCAGCTGGTCGAACATCAAC

DaphniaNRK ------------------------------------------------------------

LymneaTrk ------------------------------------------------------------

AmphioxusROR ------------------------------------------------------------

DrosophilaNRK ------------------------------------------------------------

AplysiaROR GCCAACAATGGTCCCAGCAAGGTGTCGCCCGCTGGTTCTGTGGCCAGCTCCAAGTCTTCA

DaphniaTrkL ------------------------------------------------------------

AmphioxusTrk ------------------------------------------------------------

DaphniaROR ------------------------------------------------------------

DpulexTrk ------------------------------------------------------------

HumanTrkB ------------------------------------------------------------

DrosophilaROR ------------------------------------------------------------

HumanROR1 AGCAGTGCCAGTGGGTCGACTAGCACTGGCCATGTGACTAGCTTGCCCTCATCAGGATCC

HumanTrkA ------------------------------------------------------------

HumanTrkC ------------------------------------------------------------

AplysiaTrkL CGTAGCAGAGACATTGCTTCGAGCATGATCGGTTCATCTTCTCCCAGCTCACACGAAGGG

DaphniaNRK ------------------------------------------------------------

LymneaTrk ------------------------------------------------------------

AmphioxusROR ------------------------------------------------------------

DrosophilaNRK ------------------------------------------------------------

AplysiaROR AACAGCGCTTCCTCCACCCACAACAGCGGGGGTGTTGGGGGCGTGCCTCCCCGACAGGGC

DaphniaTrkL ------------------------------------------------------------

AmphioxusTrk ------------------------------------------------------------

DaphniaROR ------------------------------------------------------------

DpulexTrk ------------------------------------------------------------

HumanTrkB ------------------------------------------------------------

DrosophilaROR ------------------------------------------------------------

HumanROR1 AATCAGGAAGCAAATATTCCTTTACTACCACACATGTCAATTCCAAATCATCCTGGTGGA

HumanTrkA ------------------------------------------------------------

HumanTrkC ------------------------------------------------------------

AplysiaTrkL AACAAACCCGCCACTTTCAGAATTCTCAAAGCATCCACAAAATGCACCGGGCGTTCAGGA

DaphniaNRK ------------------------------------------------------------

LymneaTrk ------------------------------------------------------------

AmphioxusROR ------------------------------------------------------------

DrosophilaNRK ------------------------------------------------------------

AplysiaROR ATGGCCCACGCCGGTCAGAACACGAGCGGACAGCCAATGATGAACGCTAACTACAAGCTC

DaphniaTrkL ------------------------------------------------------------

AmphioxusTrk ------------------------------------------------------------

DaphniaROR ------------------------------------------------------------

DpulexTrk ------------------------------------------------------------

HumanTrkB ------------------------------------------------------------

DrosophilaROR ------------------------------------------------------------

HumanROR1 ATGGGTATCACCGTTTTTGGCAACAAATCTCAAAAA---------CCCTACAAAATTGAC

HumanTrkA ------------------------------------------------------------

HumanTrkC ------------------------------------------------------------

AplysiaTrkL CGAGGGAGAAAAGTCAAAGGTTCCCCCTCGCGATTTGCTGATCGTCTTCCTTCCTTCAGT

DaphniaNRK ------------------------------------------------------------

LymneaTrk ------------------------------------------------------------

AmphioxusROR ------------------------------------------------------------

DrosophilaNRK ------------------------------------------------------------

AplysiaROR CAACCACAGGCTTTCAACGGAGGCGGGGCCTCGAACATTTCCACCCCGCCCCCTGTCAGT

DaphniaTrkL ------------------------------------------------------------

AmphioxusTrk ------------------------------------------------------------

DaphniaROR ------------------------------------------------------------

DpulexTrk ------------------------------------------------------------

HumanTrkB ------------------------------------------------------------

DrosophilaROR ------------------------------------------------------------

HumanROR1 TCAAAGCAAGCATCTTTACTAGGAGAC---------------------------GCCAAT

HumanTrkA ------------------------------------------------------------

HumanTrkC ------------------------------------------------------------

AplysiaTrkL CGCCTTGACGACGCTATAGGAGCAGGGTTACCCACACCCGTGAGRGAATCAGCGCCTCTG

DaphniaNRK ------------------------------------------------------------

LymneaTrk ------------------------------------------------------------

AmphioxusROR ---------------------------------------------------AACCCCCCC

DrosophilaNRK ------------------------------------------------------------

AplysiaROR ATTGCTGAGTGCAATGGGTACAACAGC---------------------------TTTTCG

DaphniaTrkL ------------------------------------------------------------

AmphioxusTrk ---------------------------------------

DaphniaROR ------------ACAAGCCGGAGTCATTCAGGTTGGTTA

DpulexTrk ---------------------------------------

HumanTrkB ---------------------------------------

DrosophilaROR ---------------------------------------

HumanROR1 ATTCATGGACACACCGAATCTATGATTTCTGCAGAACTG

HumanTrkA ---------------------------------------

HumanTrkC ---------------------------------------

AplysiaTrkL AACGAGTACTCGATACCAAAGTCGAGTCATTCATTGCTG

DaphniaNRK ---------------------------------------

LymneaTrk ---------------------------------------

AmphioxusROR TACCACAGACACACGCCCCCCATCCCCAACGGCTATCCC

DrosophilaNRK ---------------------------------------

AplysiaROR CATTCTGCTTACAGCCCTGATCAGAGAACATCGAACATC

DaphniaTrkL ---------GACCTGCCCGTCCACCACTACTCCAACACA
