# Supplementary material for: The genome sequence of the protostome Daphnia pulex encodes respective orthologues of a neurotrophin, a Trk and a p75NTR: Evolution of neurotrophin signaling components and related proteins in the bilateria
Source: BMC Evol Biol. 2009 Oct 6;9:243. doi: 10.1186/1471-2148-9-243 (PMC2772990; doi:10.1186/1471-2148-9-243)
Supplement: Additional file 3 — Nucleotide alignment supporting the RTK tree with sequence portions encoding the tyrosine kinase domain only. The data represents an alignment of nucleotide sequences encoding the tyrosine kinase domain of RTK proteins. This alignment was used to compute the phylogenetic tree presented in Figure 9B. [file 1471-2148-9-243-S3.DOC]

CLUSTAL X (1.64b) multiple sequence alignment - created by revtrans

DrosophilaROR GTTGAGTTTCTGGAGGAGCTGGGCGAAGGAGCTTTTGGAAAAGTC---------------

SeaUrchinROR ATCAAGTTCAGCGAGGCCCTTGGGGATGGCACGTTTGGCAAGGTA---------------

ChickenTrkB ATTGTTCTCAAAAGAGAACTGGGAGAAGGAGCCTTTGGAAAAGTG---------------

HoneyBeeROR GTTGTACTATTACAAGAACTTGGAGAAGGAGCTTTCGGAAAAGTA---------------

LottiaTrkL TTAAAATTGTCAACTGTGGTTGGAGAAGGGGCATTTGGGCAGGTG---------------

PeaAphidTrkL ATATCGTCAATGGAAGAAATCGGGCAAGGATTTTTCGGAAAAGTT---------------

DaphniaTrk TTGCATTCGCTCAAAGACTTGGGAGAAGGTGTTTTCGGCAAAGTT---------------

JewelwaspNRK ATTATATACGTGAGAGATCTAGGCCAGGGAGCCTTTGGCAGAGTT---------------

AmphioxusROR GTGCGCTTCCTCACCGAGCTGGGAGAGGGCGCTTTTGGCAAGGTG---------------

DrosophilaNRK ATAGTGTATGTGAGATCATTGGGTCAAGGAGCCTTCGGTCGCGTC---------------

JewelWaspROR GTCATGTTATTACAAGAGCTAGGAGAAGGAGCCTTTGGAAAGGTT---------------

CapitellaROR ATTCGCTTCTTGGAAGAACTGGGAGAGGGAACCTTCGGCAAGGTT---------------

ChickenTrkA ATTGTGCTCAAGTGGGAGCTGGGAGAGGGCGCCTTTGGGAAGGTC---------------

ZebrafishTrkA ATTGTTTTGAAGTGGGAACTGGGTGAAGGAGCTTTTGGCAAGGTT---------------

ZebrafishTrkC2 ATCGTGCTCAAGAGGGAACTGGGCGAAGGGGCCTTTGGGAAGGTG---------------

DaphniaNRK ATTATCTACATCAGAGACATTGGCCAGGGAGCATTCGGCCGGGTG---------------

PeaAphidROR ATCACATTTTTACAGGAAATTGGAGAAGGCGCTTTTGGCAAAGTT---------------

PeaAphidNRK ATAATATATGTAAAAGACTTGGGCCAAGGAGCATTTGGGCGTGTA---------------

LottiaNRK GTGGTGTATATACGTGATCTGGGACAAGGTGCCTTCGGTCGTGTT---------------

AplysiaTrkL CTCACTCTCATCGAGGTGGTGGGCGAGGGGGCGTTTGGACAGGTG---------------

SeaUrchinTrk ATCCGGTTCATCGGCGAGCTTGGGGAAGGTGCGTTCGGCGTGGTG---------------

CapitellaTrk ATCCACATGGTGAGGGAGCTGGGTGAGGGAGCATTCGGTCGAGTG---------------

AplysiaROR ATCCGCTTTCTTCAGGAACTTGGCGAAGGGGCTTTCGGGAAGGTC---------------

ChickenROR GTTCGTTTCATGGAAGAACTGGGTGAATGTGCTTTCGGCAAAATC---------------

CapitellaNRK GTGGTGTATGTACGGGATATCGGCCAGGGGGCTTTTGGACGCGTA---------------

ChickenTrkC ATCGTTTTGAAGAGGGAGCTGGGAGAAGGTGCCTTTGGGAAGGTG---------------

ZebrafishTrkB1 ATTGTGCTAAAGAGGGAACTTGGAGAGGGAGCATTTGGGAAAGTG---------------

HoneyBeeNRK ATTATATATGTTCGAGATTTGGGACAGGGCGCTTTTGGTAGAGTA---------------

HumanROR GTACGCTTTATGGAAGAATTGGGTGAGTGTGCCTTTGGAAAAATC---------------

ZebrafishTrkB2 ATTTTACTGAAGAGGGAGCTGGGAGAGGGGGCTTTCGGGAAAGTG---------------

JewelWaspTrkL CTCGTCTTCCTCCAAGACATCGGCGAGGGCTGCTTCGGAAAAGTC---------------

LottiaTrk ATTACCTTTATAAGAAGCTTAGGTGAAGGAGCGTTCGGTCGAGTG---------------

DaphniaTrkL ATCGAATTGAAACAGGAAATTGGAGAGGGCTGCTTTGGCAAAGTC---------------

LymneaTrk ATACTGCTCATGCGCGTCATCGGGGAGGGAGCCTTTGGGCGCGTG---------------

DrosophilaDTrk AGCACCACGCTAAGTACTCTAAATGAGAAGCGTCGCTCCAAGACC---------------

AmphioxusTrk ATCCACTTCGTGGGCGAGCTCGGGGAGGGGGCCTTCGGAAGAGTC---------------

DaphniaROR GTGCGGTTCCAGCACGATCTCGGAGAAGGAGCTTTTGGCAAAGTAAGAAAAATCAAATTA

ZebrafishTrkC1 ATCGTGCTCAAGAGGGAACTGGGCGAAGGGGCCTTTGGGAAGGTG---------------

HumanTrkC ATCGTGCTGAAGCGAGAACTGGGTGAGGGAGCCTTTGGAAAGGTC---------------

HoneyBeeTrkL CTTGCATTTTTGCAAGAAATTGGCGAAGGGTGTTTTGGAAAAGTA---------------

HumanTrkA ATCGTGCTCAAGTGGGAGCTGGGGGAGGGCGCCTTTGGGAAGGTC---------------

LottiaROR GTAAGATTTCTACAGGAGCTGGGAGAAGGAGCATTTGGGAAGGTC---------------

HumanTrkB ATTGTTCTGAAAAGGGAGCTAGGCGAAGGAGCCTTTGGAAAAGTG---------------

DrosophilaROR ---TACAAGGGA---------CAG---------------CTCCTGCAGCCGAACAAAACC

SeaUrchinROR ---TGGCGAGGA---------GAG---CTGATG------GGTCTAAACAACCAGTACAGC

ChickenTrkB ---TTTCTGGCA---------GAA---TGTTAC------AACCTCTGTCCCGAACAGGAC

HoneyBeeROR ---TATAAGGGA---------GAA---TTACAA------ACGGGCAATAAATGCGAACCT

LottiaTrkL ---TTTAAAGGT---------GAG------------------CTCACTGACGACAATGGT

PeaAphidTrkL ---TACAAAGGT---------ATC------------------CTCCAACAGACCGAAGGC

DaphniaTrk ---CACCTGTCG---------ACTTACTCCTCG------TCGCAAGACCCGGAAGCCGGA

JewelwaspNRK ---TTCCAAGCG---------AAG---GCGCCA------GGATTAGTGCCCAACGAAGAG

AmphioxusROR ---TACAAAGGT---------GAG---CTGGTGGTCACGGGGTCAGGCGAGGACGCCAAG

DrosophilaNRK ---TTCCAGGCC---------AGG---GCTCCT------GGACTTGTTCCCGATCAGGAA

JewelWaspROR ---TATAAAGGA---------GAA---TTACAA------ACAGGAAATAAGGCTGATGAA

CapitellaROR ---TATAAAGGA---------GAA---CTGCTT------GGTTACTATGGAGATGGCACT

ChickenTrkA ---TTCCTGGCC---------GAG---TGTTCC------CACCTCCTCCCGGAGCAGGAG

ZebrafishTrkA ---TATCTGGCT---------GAA---TGTGCC------AATCTCTGTCCTGATACTGAC

ZebrafishTrkC2 ---TTCCTGGCA---------GAG---TGTTAC------AACCTAAGCCCCACTAAGGAC

DaphniaNRK ---TTTCAAGCC---------AAA---GCTCCG------AATCTGGTCAAAGGTGAGGCC

PeaAphidROR ---TTCAAAGGT---------CAA---GTGGCC------AATGATATGGGT------ACC

PeaAphidNRK ---TTCCAGGCC---------AAA---GCACCT------GGTCTGCTGAAAGACGAAGAA

LottiaNRK ---TTTAAAGCA---------AAA---GCTCCT------GAATTAGTAAAAGGAGAAACA

AplysiaTrkL ---TTCAGAGGA---------GAA------------------CTGCGCAGCGCAGAGGGC

SeaUrchinTrk ---TGCCTGGGA---------CAG---TGTGAG------CACCTCCCCGGCGCAGATGGT

CapitellaTrk ---TACCTCGGA---------CTC---TGCGAA------GGTTTGACCCCCAACGATGAC

AplysiaROR ---TACAAGGGG---------GAA---CTGGTC------GGCTTGTACGGAGAGAGCTCT

ChickenROR ---TACAAGGGA---------CAC---CTCTAC---------CTTCCAGGCATGGACCAC

CapitellaNRK ---TTCCAAGCG---------AAG---GCCCCG------GGAGTCACACATGGTCATGAT

ChickenTrkC ---TTCTTGGCC---------GAG---TGTTAC------AACCTCAGCCCCACCAATGAC

ZebrafishTrkB1 ---TTCCTGGCT---------GAG---TGCTAC------AATCTGTCGGCAGACCAGGAG

HoneyBeeNRK ---TTTCAAGCA---------AAA---GCACCT------GGTCTTGTTCCAGGTGAAGAA

HumanROR ---TATAAAGGC---------CAT---CTCTAT---------CTCCCAGGCATGGACCAT

ZebrafishTrkB2 ---TTCTTAGCT---------GAA---TGCTAC------AACTTGTCTCCTGACCAAGAG

JewelWaspTrkL ---TTCAAAGGT---------GAG---CGACCACCAGGTGAGCTGACCTGCGAAGACGCG

LottiaTrk ---TACCTAGCA---------ACA---TGCTAT------GGTTTATTAGCAGAAGATGAT

DaphniaTrkL ---TTTCGTGGATCGCTGCGACGGCCACCATCGGCGCCCAACGCACAACCAGACGACGAA

LymneaTrk ---TTTCTGGGC---------ACG---TGCGCC------CACCTGATCCAGAAGAACGAG

DrosophilaDTrk ---TCCATGGACGACATTGAGGAGATCAAGGAGGAGGAGCAGGATCAGCACAATCAATCG

AmphioxusTrk ---TACCTCGGC---------AAA---TGCGAG------AAGCTTAAACCCGACGAAGAT

DaphniaROR ATTTACCCAGGT---------CAG---ATTTAC---------TTAAATCCCGGTGGTGTC

ZebrafishTrkC1 ---TTCCTGGCA---------GAG---TGTTAC------AACCTAAGCCCCACTAAGGAC

HumanTrkC ---TTCCTGGCC---------GAG---TGCTAC------AACCTCAGCCCGACCAAGGAC

HoneyBeeTrkL ---TACAAAGGA---------GAA------------------TTATGCATAGGGGATTCG

HumanTrkA ---TTCCTTGCT---------GAG---TGCCAC------AACCTCCTGCCTGAGCAGGAC

LottiaROR ---TATAAAGGA---------GAG---CTAATG------GGACTGTATGGTGACAATACT

HumanTrkB ---TTCCTAGCT---------GAA---TGCTAT------AACCTCTGTCCTGAGCAGGAC

DrosophilaROR ------------------------------ACC------ATAACAGTTGCCATCAAGGCG

SeaUrchinROR ------------------------------TGT------ACATCCATCGTCATCAAGACA

ChickenTrkB ------------------------------AAG------ATCTTGGTAGCAGTGAAGACT

HoneyBeeROR ------------------------------CCA------ATTTATGTGGCAGTGAAAACG

LottiaTrkL ------------------------------GACTTTGTACAAAATGTAGCAATTAAAGTT

PeaAphidTrkL ------------------------------AAATAT---GAAACAGTCGCCATAAAAGTC

DaphniaTrk ------------------------------AAG------TTTCTCGTCGCTGTGAAAATT

JewelwaspNRK ------------------------------TTT------ACTAACGTGGCTGTAAAAATG

AmphioxusROR ------------------------------AAG------ATCCTGGTGGCCATCAAGACG

DrosophilaNRK ------------------------------GAT------CTACTAGTCGCTGTTAAGATG

JewelWaspROR ------------------------------GTA------ATTTATGTAGCTGTTAAAACT

CapitellaROR ------------------------------GTT------ATGAAGGTGGCCATCAAAACG

ChickenTrkA ------------------------------AAG------ACATTGGTGGCTGTGAAGGCG

ZebrafishTrkA ------------------------------AAG------ATGCTGGTTGCTATCAAGACT

ZebrafishTrkC2 ------------------------------AAG------ATGCTGGTGGCTGTCAAGACC

DaphniaNRK ------------------------------TGC------TCCATTGTGGCCGTCAAGACG

PeaAphidROR ------------------------------ATA------ATTATGGTAGCCATTAAAACA

PeaAphidNRK ------------------------------TTT------ACATTGGTTGCTGTAAAAATG

LottiaNRK ------------------------------TTT------ACTTTTATAGCTGTTAAAATG

AplysiaTrkL ------------------------------GGGGCTTGTCATCAAGTAGCTGTCAAAGTA

SeaUrchinTrk ------------------------------CCG------ACCATGGTAGCGATCAAGACC

CapitellaTrk ------------------------------CTC------ACGATGGTGGCGATCAAGACG

AplysiaROR ------------------------------GTT------ACGACAGTGGCAATCAAGACG

ChickenROR ------------------------------GCT------CAGCTCATTGCAATCAAGACA

CapitellaNRK ------------------------------TGG------AGATATGTTGCAGTGAAGATG

ChickenTrkC ------------------------------AAA------ATGCTGGTGGCAGTGAAGGCG

ZebrafishTrkB1 ------------------------------AAG------ATCTTAGTGGCTGTCAAGACT

HoneyBeeNRK ------------------------------TTT------ACCAATGTTGCTGTAAAAATG

HumanROR ------------------------------GCT------CAGCTGGTTGCTATCAAGACC

ZebrafishTrkB2 ------------------------------AAG------ATTTTGGTGGCTGTTAAGACT

JewelWaspTrkL ------------------------------ACG------GAGATAGTGGCGATAAAGGTC

LottiaTrk ------------------------------GTG------ACAATGGTGGCCATCAAAATG

DaphniaTrkL CAAGAAGAAGAAGAAGATGAAGAAATTCACTTTGATGATGAAGCGGTGGCTGTCAAAGTT

LymneaTrk ------------------------------TTT------GCGATCGTCGCCGTGAAGACG

DrosophilaDTrk ------------------------------GGTCTCGAGCAGCTCGTTCTGGTCAAAGCC

AmphioxusTrk ------------------------------GCC------TCCCTGGTGGCGGTGAAGACG

DaphniaROR ---------------------------------------ATGCCCATTGCCATCAAGACG

ZebrafishTrkC1 ------------------------------AAG------ATGCTGGTGGCTGTCAAGACC

HumanTrkC ------------------------------AAG------ATGCTTGTGGCTGTGAAGGCC

HoneyBeeTrkL ------------------------------AAA------GAGATCGTCGCTATAAAAGTT

HumanTrkA ------------------------------AAG------ATGCTGGTGGCTGTCAAGGCA

LottiaROR ------------------------------GTA------ACAAAAGTAGCAATTAAAACT

HumanTrkB ------------------------------AAG------ATCTTGGTGGCAGTGAAGACC

DrosophilaROR TTGAAGGAAAACGCCTCGGTGAAAACGCAGCAGGACTTTAAGCGCGAAATCGAACTAATC

SeaUrchinROR TTGGACAAGGAAGCGAGCCCCGCCCTTCAGCAGGACTATCGTAACGAGACCAGTGTGATG

ChickenTrkB TTGAAG---GATGCCAGTGACAATGCCCGCAAGGACTTCCACCGTGAGGCAGAGCTGCTG

HoneyBeeROR TTGAAAGAGAATGCAAGTCCAAAAACACAGAGCGACTTCAAACGGGAAGTCGATTTGATG

LottiaTrkL TTAAAAGAAGGAGCTACAAATGAACTTAAAGAAGATTTTTATAGAGAAGTTGAAATCATG

PeaAphidTrkL TTAAAGGATCACACTAACTTGGAGGCCAAAGAAGACTTTATGAGGGAAGTAGAGATAATG

DaphniaTrk CTCAAG---TTTTGCGGAGAAGAATCCGCCAAGGATTTCGACCGTGAAGCGTCGCTTTTG

JewelwaspNRK CTAAAAGAAGAGGCCTCTGACGATCTACTCGTAGATTTTGAACGAGAGGCTTGCCTCCTC

AmphioxusROR CTGAAGGAGAACGCGACCTTGAAGACGCAGCACGATTTCCACCGCGAGGTCGACATGTTG

DrosophilaNRK CTAAAGGACGACGCCAGCGACCAGATGCAGATGGATTTCGAGCGCGAGGCCTGTTTGCTG

JewelWaspROR TTAAAAGAAAACGCAAGTCCAAAAACTCAAAGTGACTTCAAACGCGAAGTTGATCTCATG

CapitellaROR CTGAAGGAGAATGCTGTCTCCAAAGTGCAGCATGATTTCAGGAGGGAAGTGGACCTGATG

ChickenTrkA CTGAAG---GAGGTGACGGAGAACGCGCGGCTCGACTTCCAGCGGGAAGCAGAACTGCTG

ZebrafishTrkA TTGAAA---ATAGCCAATGAGTCCACCCGGCAGGACTTCCAGCGCGAGGCTGAGCTGCTC

ZebrafishTrkC2 TTGAAG---GATCCCACACTTGCGGCAAGGAAAGATTTCCAGCGTGAGGCCGAATTGTTG

DaphniaNRK CTGAAGGAGGAGGCCGACGATGAAATGTGCGCCAATTTCGAGAAGGAGGCCTGCCTATTG

PeaAphidROR TTGAAAGAAGGTGCAAGCGCTAAGACTGCAGCAGACTTTAAACGTGAAACTGATTTAATG

PeaAphidNRK TTAAAAGATGAAGCTTCAAACGATTTACAAGTTGATTTTGAACGAGAAGCTTGCCTATTG

LottiaNRK TTAAAGGAAGATGCTTCAGAAGACTTACAAGCTGATTTTGAACGTGAAGCTTCCTTATTG

AplysiaTrkL CTCAAGGACGGGGCGTCGCCGGACGCCCATGAAGACTTCGAGAGGGAGGTTGAGATCATG

SeaUrchinTrk CTCAAAGACGCCTCCGTGGGCGACGCCAGGACCGACTTTGAAAGAGAGGCCGAGTTGCTG

CapitellaTrk CTGAAGGACAATTGCCAAGAAGACTTGAGGAAGGACTTTGACCGCGAAGCAGAACTACTC

AplysiaROR CTGAAGGAGAACGCGTTGCCCAAAGTGCAGAATGACTTTCGACGAGAGGTTGACCTGATG

ChickenROR CTAAAAGACTTTAACAACCCCCAGCAGTGGGCAGAGTTCCAGCAAGAAGCATCTCTTATG

CapitellaNRK CTAAAAGATGACGCAACAGATGATATGCAGAGGGATTTTGAGCGAGAAGCTCTTCTCATG

ChickenTrkC CTGAAA---GACCCCACCTTGGCAGCCCGCAAGGATTTCCAGAGGGAGGCAGAGCTGCTC

ZebrafishTrkB1 TTAAAA---GAGGCCAGTGAGAATGCCAGGAAGGACTTCCACCGCGAGGCCGAGCTGTTG

HoneyBeeNRK TTAAAAGAAGAAGCATCAGATGATCTGCTCAAAGATTTCGAACGAGAAGCATGTCTTCTT

HumanROR TTGAAAGACTATAACAACCCCCAGCAATGGATGGAATTTCAACAAGAAGCCTCCCTAATG

ZebrafishTrkB2 TTGAAA---GAAGCCAGTGAAAGTGGGCGAGCAGATTTCCACAGAGAGGCAGAACTCCTG

JewelWaspTrkL CTAAAGGACACGGCGAGCCGCGAGGCCGAGGAGGACTTCATGCGCGAGGTCGACATAATG

LottiaTrk TTAAAAAATGCCAGCATTGAGTCAATAAAAAAGGATTTTGAAAGAGAAGCTGAATTGTTG

DaphniaTrkL TTAAAAGCCGCAGCTGGACCAGCTGCCCAGGAGGATTTGCTCCAGGAAGCTGAAATTATG

LymneaTrk CTGAAAGGGAGCTGTAGCGATTCCTTGAAGAGAGACTTTGAAAGAGAAGCAGAGATGCTG

DrosophilaDTrk CTGAATAAAGTGAAGGATGAACAGGCCTGCCAGGAGTTTCGACGACAGTTAGATCTGCTG

AmphioxusTrk CTGAAGGAGATGAGTGTCGAGGACGCGCGGAAGGACTTTGATCGGGAAGCGGAGCTCCTG

DaphniaROR CTGAAAGCAAACGCTTCGGTGAAAACCCAGCAAGATTTTCGCCGTGAAGTGGAGCTCATG

ZebrafishTrkC1 TTGAAG---GATCCCACACTTGCGGCAAGGAAAGATTTCCAGCGTGAGGCCGAATTGTTG

HumanTrkC CTGAAG---GATCCCACCCTGGCTGCCCGGAAGGATTTCCAGAGGGAGGCCGAGCTGCTC

HoneyBeeTrkL TTGAAAGAGACTGCACCGCGAGAAGCTGAAGAAGATTTTATGCGAGAAGTCGACATCATG

HumanTrkA CTGAAG---GAGGCGTCCGAGAGTGCTCGGCAGGACTTCCAACGTGAGGCTGAGCTGCTC

LottiaROR CTGAAGGAAAATGCAGCACCTAAAGTTCAAAATGATTTTCGGAGAGAAGTGGATCTAATG

HumanTrkB CTGAAG---GATGCCAGTGACAATGCACGCAAGGACTTCCACCGTGAGGCCGAGCTCCTG

DrosophilaROR TCGGATCTAAAGCATCAGAATATAGTGTGCATATTGGGCGTAGTGCTCAATAAGGAG---

SeaUrchinROR GCGTCCCTCAACCATCCCAACATCATCACTCTCTTAGGGGTCTGCACCAAGGAGAAG---

ChickenTrkB ACTAATCTGCAACATGAGCACATTGTCAAGTTCTATGGTGTCTGTGTCGAGGGTGAT---

HoneyBeeROR ACAGACCTAAGGCATCCAAATATTATTTGCCTACTGGGCGTAATACTGAAAGGGGAA---

LottiaTrkL AGTACTTTTGATCATGATAACATTTTACATTTACTGGGGATAGTCACACAAAATGCTGGG

PeaAphidTrkL ACGTATTTCAGACATCCGAATATCTTGACATTGATCGGAGTTTGCCCCCAAGATGATAAT

DaphniaTrk ACTCAGCTGACTCACAAAAACATTGTCCAATTTCACGGAGCCTGCGTCGACGAAAAA---

JewelwaspNRK TCGGAATTCGACCACCCGAACATCGTCAAACTTTTAGGCGTTTGCGCTCTGGGACGT---

AmphioxusROR GCTGACCTGCGTCACCAGAACATCGTGTGTCTGCTGGGGGTGGTGATGCGGGACCAG---

DrosophilaNRK GCCGAGTTCGATCATCCCAATATCGTGAGGCTGCTGGGGGTGTGCGCCTTGGGCAGA---

JewelWaspROR ACAGATCTTAGGCATCCAAATATTATTTGCTTACTTGGTGTAATACTTAACGGAGAG---

CapitellaROR AGCGATCTACAGCATCCGCACATCGTGTGTCTGCTAGGTGTGTGCATCAAGGAGCAT---

ChickenTrkA ACGGTGCTGCAGCACGAACACATCGTCAAGTTCTACGGCGTGTGCACCGAGGGTGAT---

ZebrafishTrkA ACGGTGCTTCAGCACGAACACATTGTGCGCTTCTACGGTGTGTGCGCAGACGGAGAA---

ZebrafishTrkC2 ACCAACCTTCAGCACGAGCACATAGTCAAGTTCTATGGCGTCTGTGTGGACGGAGAC---

DaphniaNRK GCCGAGCTGGACCATCCCAACATAATCGGCTTGCTGGGCGTCTGCGCCGTCGGCAAG---

PeaAphidROR GGAGAACTACGACATCCAAATGTGGTTTGCTTGGTAGGCATGTGTTCACGT---------

PeaAphidNRK TCAGAATTTGATCATCCTAACATTGTTAAGTTATTGGGAGTTTGTGCTTTAGGTAAA---

LottiaNRK GTTGAATTTAATCATCCAAACATTGTGAAATTATTGGGTGTATGTGCAGTAGGAAAA---

AplysiaTrkL TCAGCCTTTGACCATGACAACATTCTCAAGCTGCTGGGAATTGTGGTGCAAGGTGTGGAA

SeaUrchinTrk ACCAACTTGCAGCATAAGAACATTGTGGCGTTCTACGGAGTGTGTACCGATCACGAG---

CapitellaTrk ACCAGCCTCCACCACCAAAACATTGTCACTTTCCACGGGGTCTCTCAAGATACACAA---

AplysiaROR TCCGACATGCGTCACCCTAACATCGTATGCCTCCTGGGCGTGTGCATGAAACAAGAA---

ChickenROR GCTGAGCTCCACCACCCTAACATTGTTTGCCTCTTAGGTGTGGTGACCCAGGAGCAA---

CapitellaNRK GTGGAGTTTAACCATAAGAACATAGTGCGTTTATTAGGTGTATGTGCTGTGGGTAAA---

ChickenTrkC ACCAACCTGCAGCATGAGCACATTGTCAAGTTCTATGGTGTGTGTGGTGACGGTGAC---

ZebrafishTrkB1 ACCAATCTTCAGCATGAGCACATTGTCACGTTCTACGGCGTTTGTGTGGAGAGCGAT---

HoneyBeeNRK GCCGAATTCGATCATCCAAATATCGTCAAATTATTAGGTGTATGCGCTTTAGGTCGA---

HumanROR GCAGAACTGCACCACCCCAATATTGTCTGCCTTCTAGGTGCCGTCACTCAGGAACAA---

ZebrafishTrkB2 ACCAACCTGCAGCATGAGCACATTGTCAAATTTTACGGCGTGTGTGTAGAAAGTGAC---

JewelWaspTrkL TCGACCTTCCGCCACGACAACATCCTCTCTCTCGTCGGAGTCGTTCTGCGCGACAGCAGC

LottiaTrk ACCAATCTACAACATCATCACATTGTCAAATTTTATGGTGTCAGTGTTGATGGTGAT---

DaphniaTrkL GTTTCCTTCTCTCATCCAAACATCCTCTCGCTCAAAGGGATCGTCATCAACGAGCCCAAT

LymneaTrk GCCACGATAGAGCACGCCAACATTGTCACATTCTACGGCGTGTGTACGGAGAGTGAT---

DrosophilaDTrk CGCGCCATCTCGCACAAGGGAGTAGTACGTCTGTTTGGCCTGTGTCGCGAAAAGGAT---

AmphioxusTrk ACCAACATGCAGCATGAGAACATTGTGAAGTTTTACGGGGTCTGCACGGAGGGAGAG---

DaphniaROR TCTGAATTGCGTCATCCCAATATCGTATGTCTATTAGGAGTCGTCACACGGGATCAA---

ZebrafishTrkC1 ACCAACCTTCAGCACGAGCACATAGTCAAGTTCTATGGCGTCTGTGTGGACGGAGAC---

HumanTrkC ACCAACCTGCAGCATGAGCACATTGTCAAGTTCTATGGAGTGTGCGGCGATGGGGAC---

HoneyBeeTrkL TCCACATTTGGGCATAGAAATATTTTGTCGTTGAAGGGTGCAGTGCTTCGTGAAGGTAAT

HumanTrkA ACCATGCTGCAGCACCAGCACATCGTGCGCTTCTTCGGCGTCTGCACCGAGGGCCGC---

LottiaROR TCTGAAATGCGGCATCCGAATATTGTGTGTTTATTAGGTGTGTCTATGAAACAGGAT---

HumanTrkB ACCAACCTCCAGCATGAGCACATCGTCAAGTTCTATGGCGTCTGCGTGGAGGGCGAC---

DrosophilaROR ------CCCTACTGCATGCTGTTCGAGTACATGGCCAATGGTGATCTGCACGAATTCCTA

SeaUrchinROR ------CCCAACTGTATGTTGTTTGAGTTCTTACCTCATGGTGATCTGCATGAATTCCTA

ChickenTrkB ------CCACTCATCATGGTCTTTGAGTACATGAAGCATGGAGATCTGAACAAATTCCTC

HoneyBeeROR ------CCAATGTGCATGTTATTCGAGTACATGACCCAAGGCGATTTACACGAGTTTCTC

LottiaTrkL GAATCGCCGTAT---ATGGTCTTTGAGTTTATGATACATGGTGATCTAGCTGAATTACTA

PeaAphidTrkL TGTTCACCGTGG---ATGATATTCGAGTTCATGGCATATGGAGATTTAACCGAAGTTCTG

DaphniaTrk ------CCCTGGAAAATGGTCTTCGAGTACATGGAAAACGGAGATTTGAACCAGTTTTTG

JewelwaspNRK ------CCCATGTGTCTCCTGTTCGAGTACATGGGTCGGGGAGACTTGAACGAATTCCTA

AmphioxusROR ------CCCATGTGCATGCTGTTCGAGTACATGCGCTACGGAGACCTACACGAGTTCCTG

DrosophilaNRK ------CCCATGTGCCTGCTCTTCGAGTACATGGCTCCTGGCGATCTAAGCGAGTTCTTG

JewelWaspROR ------CCAATGTGTATGCTTTTTGAGTATATGACTCAAGGAGACTTGCATGAATTTCTG

CapitellaROR ------CCTCGCTGCATGCTCTTTGAGTACATGGCCAATGGGGACCTCCACGAGTACCTC

ChickenTrkA ------CCCCTCATCATGGTCTTCGAGTACATGAAGCATGGAGACCTCAACCGCTTCCTC

ZebrafishTrkA ------CCCCTGGCCATGGTGTTTGAGTACATGCGACACGGAGACCTCAACCGCTTTCTA

ZebrafishTrkC2 ------CCACTCATCATGGTCTTCGAGTACATGAAACATGGAGACCTCAACAAGTTCCTC

DaphniaNRK ------CCCATGTGTCTCCTGCTCGAATTTATGGAGCTGGGCGACCTGAGACAATACTTG

PeaAphidROR ------CCAGCATGCTTATTGTTTGAATACATGGTTGGTGGAGACTTACATGAATTCCTT

PeaAphidNRK ------CCAATGTGTTTACTTTTTGAATACATGGGCCGTGGGGACTTAAATGAATTTCTT

LottiaNRK ------CCGATGTGTTTATTATTTGAGTATATGAGTAAAGGGGATCTAAATGAATTCCTG

AplysiaTrkL GGAGCTCCTTAC---ATGGTGTTTGAGTACATGGAACACGGAGATCTCTCCGAGCTGTTG

SeaUrchinTrk ------CCGTTCTTTATGGTCTTCGAGTATATGGAGAATGGAGACCTTAACAACTATCTC

CapitellaTrk ------CCTTTTATGATGATTTTCGAGTACATGGAGAATGGTGATTTAAATAACTTCTTG

AplysiaROR ------CCCATGTGCATGTTGTTCGAGTACATGGCTCAAGGCGACTTGCACGAGTACTTG

ChickenROR ------CCTGTCTGCATGCTCTTCGAGTACATGAACCAAGGAGACCTCCACGAGTTCCTC

CapitellaNRK ------CCCATGTGCCTAATCTTTGAATACATGAGCAAAGGGGACCTGAATGAGTTCCTC

ChickenTrkC ------CCGCTCATCATGGTCTTCGAGTACATGAAGCATGGGGACCTGAACAAGTTCCTG

ZebrafishTrkB1 ------CCTCTCATTATGGTGTTTGAGTACATGATGCACGGCGACCTCAACAAGTTCCTC

HoneyBeeNRK ------CCAATGTGTCTTCTTTTTGAATACATGGGTCGTGGTGATTTGAATGAATTTCTC

HumanROR ------CCTGTGTGCATGCTTTTTGAGTATATTAATCAGGGGGATCTCCATGAGTTCCTC

ZebrafishTrkB2 ------CCCCTCATTATGGTGTTTGAGTACATGAAACACGGAGATCTCAACAAGTTTCTC

JewelWaspTrkL AGCAGTCCCTGG---ATGGTCTTCGAGTACATGCCCTACGGTGACTTGGCCGAGGTTCTC

LottiaTrk ------GAAATGCTGATGATATTTGAGTATATGATGAATGGTGATCTCAATAATTATATT

DaphniaTrkL ATTGGACCTTGG---CTCGTGTTCGAGTACATGGCGCTGGGAGATCTGGCCCAGCTTCTA

LymneaTrk ------CAATGGATGATGATCTTTGAGTTCATGGAGAACGGGGATCTCAATAAGTACTTG

DrosophilaDTrk ------CCGCACTACATGGTGCTGGAGTACACGGATTGGGGCGATCTCAAGCAGTTCCTG

AmphioxusTrk ------CCCTGGCTGATGATCTTTGAGTACATGGAAAACGGGGACCTCAACAACTACCTG

DaphniaROR ------CCGCAATGCATGCTGTTTGAATACATGGCGCAGGGAGATCTCCATGAATTTTTG

ZebrafishTrkC1 ------CCACTCATCATGGTCTTCGAGTACATGAAACATGGAGACCTCAACAAGTTCCTC

HumanTrkC ------CCCCTCATCATGGTCTTTGAATACATGAAGCATGGAGACCTGAATAAGTTCCTC

HoneyBeeTrkL AGTAGTCCATGG---ATGGTTTTCGAATACATGCCGTATGGAGATCTCGCTGAAGTTTTA

HumanTrkA ------CCCCTGCTCATGGTCTTTGAGTATATGCGGCACGGGGACCTCAACCGCTTCCTC

LottiaROR ------CCAATGTGTATGTTATTTGAATATATGACGCATGGTGATCTACATGAGTATCTA

HumanTrkB ------CCCCTCATCATGGTCTTTGAGTACATGAAGCATGGGGACCTCAACAAGTTCCTC

DrosophilaROR ATCTCAAACTCACCC---------------------------------------------

SeaUrchinROR GTCAGGCACTCTCCTAATTCAGACGTTGGCTTC---------------------------

ChickenTrkB AGGGCACATGGACCAGATGCAGTACTGATGGCA---------------------------

HoneyBeeROR ATTTGCCATTCACCAAGATCAGATGTTCCATTG---------------------------

LottiaTrkL AGAAAATCAGACCAAGCTGTACAA------------------------------------

PeaAphidTrkL AGAAACAGCAGTGATCAGTTTACA------------------------------------

DaphniaTrk AGAGTCCGGGGACCCGACGCCCACCTCCTGGAAGCCCGTCACGACCCAACTTGTATCAAT

JewelwaspNRK CGCTCGTGCTCTCCGGGCAACTACATAATACGT---------------------------

AmphioxusROR GTAATGCGATCCCCGCACTCCGACGTC---------------------------------

DrosophilaNRK CGCGCCTGCTCCCCATATGCCACACACCAGGCG---------------------------

JewelWaspROR ATCTGTCATTCACCAAGATCTGACGTCCCTTTA---------------------------

CapitellaROR AACACCCACTCCCCTCACTCGGACGTCAGCGTC---------------------------

ChickenTrkA AGGTCCCACGGGCCAGATGCCAAGATCCTGGAC---------------------------

ZebrafishTrkA AGGGCTCACGGTCCCGATGCTCGTATATTAGAT---------------------------

ZebrafishTrkC2 AGAGCTCATGGTCCAGATGCCATGATCCTGGTT---------------------------

DaphniaNRK CGCTCCTGCTGCCCGTCCAATTACATCGCCATG---------------------------

PeaAphidROR ATGGCCCGCTCTCCACATTCACCA------------------------------------

PeaAphidNRK CGTTCATGTGCACCTAGCAATTACATTGTACAT---------------------------

LottiaNRK CGTATTTGTAGTCCTGATCATTTTATCATTAAT---------------------------

AplysiaTrkL AGGAGGAACGACCCGCACCTGAGG------------------------------------

SeaUrchinTrk AAATCGCGAGGGCCCGATGCCGATTGTTTCACC---------------------------

CapitellaTrk AGGTCACATGGTCCAGACGCCGTGTTTTTAGGA---------------------------

AplysiaROR CTCTCCCACTCACCACACTCTGACGTCACGGCG---------------------------

ChickenROR ATCATGAGATCACCACACTCAGATGTTGGGTGT---------------------------

CapitellaNRK AGGAACTGTAGCCCTGATCACTTCATTGTGCGC---------------------------

ChickenTrkC AGGGCACATGGCCCAGATGCTATGATCCTCGTG---------------------------

ZebrafishTrkB1 AGGGCTCATGGTCCTGATGCGGTTCTGATGTCT---------------------------

HoneyBeeNRK CGATCTTGTTCACCGGGAAATTATATTATTCGA---------------------------

HumanROR ATCATGAGATCCCCACACTCTGATGTTGGCTGC---------------------------

ZebrafishTrkB2 AGGGCTCATGGTCCAGATGCGGTGCTAATGGCA---------------------------

JewelWaspTrkL CGGGCGAACTCGGCCACGCTACGC------------------------------------

LottiaTrk CGGTGCCACGGGCCAGATGCTTCTATTATTTCG---------------------------

DaphniaTrkL CGGTCCGCCAATGGCAATCTATTT------------------------------------

LymneaTrk AGGATGCACGGCCCAGACGCTGCGTTTCTGAAA---------------------------

DrosophilaDTrk CTGGCCACCGCCGGAAAAGTGAACACTGCCACC---------------------------

AmphioxusTrk AGATCTCACGGTCCGGACGCGGCCTTCCTGATC---------------------------

DaphniaROR GTAGCTCACTCACCGGCCGGCGATGGTTCGGTC---------------------------

ZebrafishTrkC1 AGAGCTCATGGTCCAGATGCCATGATCCTGGTT---------------------------

HumanTrkC AGGGCCCATGGGCCAGATGCAATGATCCTTGTG---------------------------

HoneyBeeTrkL CGATCGAACTCTCGACAATTTAAT------------------------------------

HumanTrkA CGATCCCATGGACCTGATGCCAAGCTGCTGGCT---------------------------

LottiaROR ATAGCTCATAGCCCTAATTCAGATATCTCAAAT---------------------------

HumanTrkB AGGGCACACGGCCCTGATGCCGTGCTGATGGCT---------------------------

DrosophilaROR ------------------------------------------ACCGAAGGCAAGTCGCTG

SeaUrchinROR ---------------------------GGGAGCGGAGACGACGACACCCAGTCCTCCTTG

ChickenTrkB ---------------------GAAGGC---------AACCGACCAGCTGAG------CTG

HoneyBeeROR ------------------------------------AACAATGGAAACGGAAAAATTCTG

LottiaTrkL ------------------------------------CATAGAGATAATAGTGTTGTCCTA

PeaAphidTrkL ---------------------------------AATTATTCTGCACATTTGCCGAGCTTG

DaphniaTrk ATTTCTGGTCGCCATACCGAAGACGGCGGCCCCATGGATCCTCCGCAGAAG------CTT

JewelwaspNRK ---------------------AATGTTGAGAAGGATGATACCTTTACGGACTCGCGACTG

AmphioxusROR ---------------------GGGGGCAGCTCGGATGACGCGGGGTCGCACTCGTCGCTG

DrosophilaNRK ------------------------------------CCGACACAGGATCGTCTGCAGTTG

JewelWaspROR ------------------------------------AATAATGCGAGTGGAAAAGTATTA

CapitellaROR ------------------------AGTGATCAGTGTGGAGAAGGAGCTCCAGGAGTGATG

ChickenTrkA ---------------------CAAGGGCAG---GGGCAGCCCTGCGGGCAG------CTG

ZebrafishTrkA ---------------------GAGATGAAG---GTTCCTCCGATGGGTCAG------TTG

ZebrafishTrkC2 ---------------------GACGGACAGCCGCTACAGACCAATGGAGAG------CTG

DaphniaNRK ---------CCCGAGTCGTCGGCCGGCGGATCGTCTGGCGACATCAAAGACGTCAAACTC

PeaAphidROR ------------------------------------AATTCCCCAATAGCACCACCACTT

PeaAphidNRK ------------------TCAGCTGAAGCTAGGGGTGATGTTTTTCGTGATTTAAATCTT

LottiaNRK ------------------CCTCGTAGTCATGACATATATTCTTATGAATATTCATCTTTA

AplysiaTrkL ---------------------------------AGTGCCGACTCAAAGACTTTCCGGCTG

SeaUrchinTrk ------------------------------------CGAAACCAGGCCTTACTGCCCCTG

CapitellaTrk ---------------------------------AAGAGCAGAGGAGAGGTGAAGCAACTG

AplysiaROR ---------------------GCTGAAGACGACAGCGGTACCGGAGGAGGACACATTCTG

ChickenROR ------------------------AGCAGCGATGAGGATGGAACTGTAAAATCCAGCCTG

CapitellaNRK ------------------CGGCGTAGCACAGAAGTATTGTCTCGGGACGAACCCAAACTG

ChickenTrkC ---------------------GATGGGCAGCCTCGACAAGCCAAAGGGGAG------CTA

ZebrafishTrkB1 ---------------------GACGGACAG---ATCCAGCAGCAGGCAGAG------CTC

HoneyBeeNRK ---------------------AGCCTAGAAAAAGATGAACATTTCACAGATTCTCGTTTA

HumanROR ------------------------AGCAGTGATGAAGATGGGACTGTGAAATCCAGCCTG

ZebrafishTrkB2 ---------------------GACGGACAG---CAGAGTTTGCTGGTGGAG------TTG

JewelWaspTrkL ---------------------------------ACGCCGACCCCCGGACTGCAACCGTTG

LottiaTrk ---------------------TCTAACAGGTCCAATGACTCATCTGGTGAGGAAATACTG

DaphniaTrkL ---------------------------------GCCAAAACCAAAACTGCGCATTGTTTG

LymneaTrk ---------------------GATAGAGATTCAATGGACTCAGATGAAGGGCAG---CTT

DrosophilaDTrk ---------------------------------GCGGGCAGCTCCTCACCGCCGCCACTC

AmphioxusTrk ---------------------------------AAGAACCCGGCCACTCACAAGGAGCTG

DaphniaROR ---------------TCCGGAATTGGTGCTGGAAGTGACGATGGAACAGCGAGTACGCTG

ZebrafishTrkC1 ---------------------GACGGACAGCCGCTACAGACCAATGGAGAG------CTG

HumanTrkC ---------------------GATGGACAGCCACGCCAGGCCAAGGGTGAG------CTG

HoneyBeeTrkL ------------------------------AGATCGCCGAAACCTGAGATGCAACCTTTA

HumanTrkA ---------------------GGTGGGGAGGATGTGGCTCCAGGCCCC---------CTG

LottiaROR ------------------------------TTCGAGGAAAGTGGTCAGCAAAAGATATTA

HumanTrkB ---------------------GAGGGC---------AACCCGCCCACGGAA------CTG

DrosophilaROR TCGCAGTTGGAATTCCTGCAAATAGCTCTACAAATCAGCGAAGGAATGCAGTATCTGTCG

SeaUrchinROR GATGCCTCAGATTTCTTGAACATCGCCATCCAGATCGTATCTGGCATGGATTATCTCTCT

ChickenTrkB ACACAGTCCCAGATGCTTCATATTGCCCAGCAGATTGCAGCTGGTATGGTTTACCTGGCA

HoneyBeeROR GAACAACCGGAATTTCTGCATATCGCATTACAAATAGCATCTGGTATGGAATATTTAGCC

LottiaTrkL CAGAAGACCGATCTGGTAGATATAGCAACACAGATATCAAATGGTATGGCATATCTGACG

PeaAphidTrkL GATATAGATGCACTGTTGGTGATATCATTACAAATATCGTCGGGCATGAAGTACTTGGCG

DaphniaTrk AGCTTGCTTATTTTGCTCCAAATGGCCAGAGATATTTCACAAGGGATGGAATATCTCGCG

JewelwaspNRK TCGCACATGGATCTCATAAACATCGCGCGACAGATCGCCTCAGGCATGGTGTACTTATCG

AmphioxusROR GACCACACGGACTTTCTCTGCATCGCTAACCAGATCGCGGGCGGGATGGATTATCTCGCC

DrosophilaNRK AACGAGCTACATCTGCTGCAGATGGCGGCCAACATTGCAGCGGGCATGCTGTATCTTTCG

JewelWaspROR GAGCAACCTGAATTTTTGCACATCGCTCTTCAAATTGCTTCCGGGATGGAGTATCTAGCC

CapitellaROR TTGCCTGGTGATATGATCCATGTGGCCATTCAAGTTGCGTCCGGAATGGAGTACTTGGCA

ChickenTrkA ACACTGAGCCACATGCTGCAGATCGCCACGCAGATCGCCTCAGGGATGGTTTACCTGGCA

ZebrafishTrkA ACTCTTCCTCAGATGCTCCACATTGCCGCTCAGATCGCCTCAGGAATGGTGTACCTGGCG

ZebrafishTrkC2 GGCTTGTCTCAGATGCTGCACATCGCCAGTCAGATCGCATCAGGGATGGTTTACCTGGGC

DaphniaNRK TCGGCCGCCGATTTGACGAGCATGGGCCGCCAAATCGCCGACGGAATGGTCTACCTCTCG

PeaAphidROR AACCAGGCAGACTTTATGTATATTGCTACTCAGATAGCTTCAGGCATGGAATACTTATGT

PeaAphidNRK ACAAACTTGGATATGCTGAAAATAGCTCAACAGATTGCTTCTGGTATGGTTTACCTATCC

LottiaNRK GACTCTGTTAACCAGTTGAATATTGCTAAACAAATTGCCAGTGGTATGGTATATTTATCT

AplysiaTrkL AACAAGTCTGACCTGGTGGAGATCAGCGTCCAAATTGCTACCGGAATGCGTTACCTGGCA

SeaUrchinTrk ACCGTGAAAGAACTCCTCTACATCGCTAAACAAATAGCTTCGGGTATGGTGTACATGGCT

CapitellaTrk AGTCTTCCTGAATTGCTTCACATCGCTTCGCACATTTCTGCTGGTGTCGAATATTTAGCC

AplysiaROR GAATATTCCGAAATGTTACACGTGTCCACACAGGTGGCAGCTGGCATGGAATACCTCGCC

ChickenROR GACCATKGGGATTTCCTGCATATTGCAGTTCAGATTGCAGCAYGGATGGRATACTTATCA

CapitellaNRK AATCACGTCGAGCAAGTGGACATAGCCCAGCAAATAGCTGCTGGCATGGTGTACCTTTCT

ChickenTrkC GGGCTATCCCAGATGCTCCACATCGCTAGCCAGATCGCCTCTGGAATGGTGTACCTTGCC

ZebrafishTrkB1 ACTCAGTCTCAGATGCTGCACATCGCACAGCAGATCGCCGCAGGCATGGTCTATCTGGCC

HoneyBeeNRK TCGCATATGGATTTGATTAATATTGCGCTTCAAGTAGCATCTGGAATGGTATATTTGTCA

HumanROR GACCACGGAGATTTTCTGCACATTGCAATTCAGATTGCAGCTGGCATGGAATACCTGTCT

ZebrafishTrkB2 ACTCAGCCTCAGATGCTGCACATTGCTCAGCAGATAGCAGCAGGAATGGTGTATCTGGCT

JewelWaspTrkL ACCAAGGACTCCCTCCACTGGATAACGACGCAGATAGCCGCGGGCATGACCTACCTCTCG

LottiaTrk ATGCCAGCGCAACTCTTACATATATCAAATCAAATAGCTATGGGGATGGAATATCTTGCT

DaphniaTrkL AATCAGGAGGATCTGCACTCGATCGCGGCGCAAATCGCAGACGGGATGGCCTATCTCTCG

LymneaTrk ACACGGGAACAGCTCATGAAGATTGTTTTACAGATAGCCAGTGCCATGGAGTATCTGGCG

DrosophilaDTrk ACCACCAGTCAGGTTTTGGCCGTCGCCTATCAAATTGCCCGAGGAATGGACGCCATCTAC

AmphioxusTrk AGCATCGTGGAGCTGCTGCAGATCTCTGTGCAGGTCGCCTCCGGTGTGGAGTACATGGCG

DaphniaROR GAGCAATCGGATTTCCTATACATTGCAATTCAGATCGCTGCCGGAATGGAGTATCTAGCT

ZebrafishTrkC1 GGCTTGTCTCAGATGCTGCACATCGCCAGTCAGATCGCATCAGGGATGGTTTACCTGGGC

HumanTrkC GGGCTCTCCCAAATGCTCCACATTGCCAGTCAGATCGCCTCGGGTATGGTGTACCTGGCC

HoneyBeeTrkL ACTGAGGAATCTTTACATTGGATAACAATCCAAATTGCAGCTGGTATGACATATTTATCG

HumanTrkA GGTCTGGGGCAGCTGCTGGCCGTGGCTAGCCAGGTCGCTGCGGGGATGGTGTACCTGGCG

LottiaROR GATTATTTGGATATGTTACATATTGCTACTCAGATTGGTGCTGGAATGGAATACCTTGCT

HumanTrkB ACGCAGTCGCAGATGCTGCATATAGCCCAGCAGATCGCCGCGGGCATGGTCTACCTGGCG

DrosophilaROR GCCCATCATTACGTACATCGCGACTTGGCAGCTCGGAATTGCCTGGTA------------

SeaUrchinROR AGCAGGCATTTTGTCCATAGGGACCTTGCTGCTAGGAACTGCATGGTT------------

ChickenTrkB TCACAGCACTTCGTGCATCGAGACCTTGCTACCCGGAATTGCCTGGTT------------

HoneyBeeROR AGTCATCATTATGTTCACCGAGATTTAGCCGCGAGGAATTGCCTAGTT------------

LottiaTrkL TCACAGCATTTTGTGCACCGCGATTTGGCCACCAGAAATTGTCTAGTG------------

PeaAphidTrkL AGTCAGCGGTTCGTCCACAGAGACTTGGCTTGTAGGAATTGTCTAGTC------------

DaphniaTrk TCGATGCACTACGTCCACAGGGATTTGGCGACGCGCAATTGCTTAGTG------------

JewelwaspNRK GACAGAAAGTTCGTTCATCGAGACTTGGCAACGAGAAACTGTCTGATC------------

AmphioxusROR TCCAAACACTTCTGCCACCGCGACCTGGCCGCTCGAAACTGCCTCGTC------------

DrosophilaNRK GAGAGAAAATTCGTCCACCGGGATTTGGCCACCAGGAATTGCCTGATC------------

JewelWaspROR AGCCATCACTACGTTCATAGAGATTTAGCAGCCAGAAATTGTTTGGTT------------

CapitellaROR GGACATCGTTATGTACACCGCGATCTGGCTGCGAGGAACATCTTGGTT------------

ChickenTrkA TCACTGCACTTCGTGCACCGTGACCTGGCCACACGCAACTGCCTGGTG------------

ZebrafishTrkA TCCCTTCATTTTGTCCACAGAGACCTGGCCACAAGGAACTGTCTAGTG------------

ZebrafishTrkC2 TCTCAGCACTTCGTGCACCGCGACCTGGCCACACGCAACTGCCTGGTG------------

DaphniaNRK CAGAGGGGCTTCGTCCACCGCGACCTGGCCACCCGCAACTGCCTGGTCAGCTCCAACGGG

PeaAphidROR GGCCATCATTACGTGCACAGAGATTTAGCTGCTAGAAATTGTTTAGTC------------

PeaAphidNRK GACAGAAAATTTGTACACAGAGATTTAGCGACCAGAAATTGTTTGATC------------

LottiaNRK GAGAAAGGATTTGTGCATCGCGATCTGGCCTCCAGAAACTGTCTTGTG------------

AplysiaTrkL GCTCAGAGGTTTGTCCACCGAGACCTGGCCACCCGAAACTGCCTGGTC------------

SeaUrchinTrk TCTCAACACTTCGTCCATCGAGACATGGCCACAAGGAATTGCCTTGTA------------

CapitellaTrk TCCCAACATTTTGTCCATCGTGATCTCGCAACAAGAAACTGTTTAGTG------------

AplysiaROR AGTCACCATTTCGTTCACAGAGATCTGGCCGCGAGGAACATCCTCGTG------------

ChickenROR AGCCATTTCTTTGTCCATAAAGATCTCGCCACTCGTAACATTTTAATT------------

CapitellaNRK GAGAGAGGTTTTGTGCATCGTGATCTCGCCACTCGCAACTGTTTGGTC------------

ChickenTrkC TCCCAGCACTTTGTCCATAGAGACCTGGCCACCAGGAACTGCTTGGTC------------

ZebrafishTrkB1 TCCCAGCACTTTGTGCACAGAGATCTGGCCACACGAAACTGCCTAGTC------------

HoneyBeeNRK GATCGAAAATTCGTTCATCGAGATCTCGCAACGAGAAATTGTTTGATC------------

HumanROR AGTCACTTCTTTGTCCACAAGGACCTTGCAGCTCGCAATATTTTAATC------------

ZebrafishTrkB2 TCACAGCATTTTGTGCACAGAGACCTGGCCACACGCAACTGCCTGGTG------------

JewelWaspTrkL GCGCAAAGATTCGTTCATCGGGATCTCGCCTGCAGGAACTGCCTGGTG------------

LottiaTrk TCACAACATTTTGTACACAGAGATTTAGCAACTAGAAATTGTTTAGTT------------

DaphniaTrkL TCGCAGCATTTCGTCCATCGCGACTTGGCGTGCCGCAATTGTCTGGTG------------

LymneaTrk TTGCAACATTTCGTCCATCGAGACCTGGCGACCAGAAACTGTTTGGTA------------

DrosophilaDTrk AGAGCTCGCTTCACCCATAGGGATCTGGCCACTCGCAACTGCGTAATT------------

AmphioxusTrk TCGCAGCACTTCGTGCACCGGGACCTGGCCACACGCAACTGTCTGGTG------------

DaphniaROR AGCCATCACTACGTCCATCGTGACTTGGCAGCAAGGAACTGTTTGATT------------

ZebrafishTrkC1 TCTCAGCACTTCGTGCACCGCGACCTGGCCACACGCAACTGCCTGGTG------------

HumanTrkC TCCCAGCACTTTGTGCACCGAGACCTGGCCACCAGGAACTGCCTGGTT------------

HoneyBeeTrkL GGTCAAAGATTCGTTCATAGAGATTTGGCATGTAGAAATTGTCTAGTT------------

HumanTrkA GGTCTGCATTTTGTGCACCGGGACCTGGCCACACGCAACTGTCTAGTG------------

LottiaROR AGTCATCATTTTGTTCACCGTGATTTGGCTGCTAGAAATGTGTTGGTT------------

HumanTrkB TCCCAGCACTTCGTGCACCGCGATTTGGCCACCAGGAACTGCCTGGTC------------

DrosophilaROR ------------AACGAG---------------GGTCTGGTTGTGAAGATATCCGATTTT

SeaUrchinROR ------------GGCGAG---------------CACCATCAGATTAAGATCACTGATTTT

ChickenTrkB ------------GGTGAG---------------AACCTACTGGTGAAGATTGGGGATTTT

HoneyBeeROR ------------GGGGAT---------------AATTTAACAGTAAAAATCTCCGATTTC

LottiaTrkL ------------GGTGAA---------------GGGCTGATTGTTAAAATATCAGATTTT

PeaAphidTrkL ------------GGCAAC---------------AACTTGACTGTGAAAATCGCTGATTTT

DaphniaTrk ------------GGTAAG---------------AATTTAGTGGTCAAAATAGGCGACTTT

JewelwaspNRK ------------AACGAC---------------GATATGGTAGTGAAGATAGCGGACTTT

AmphioxusROR ------------GGGGAC---------------AACCTGCTCATCAAGATATCCGATTTC

DrosophilaNRK ------------AACGAG---------------CACATGGCGGTAAAGATCGCCGACTTT

JewelWaspROR ------------GGAGAA---------------AACCTAACGGTAAAAATTTCAGACTTC

CapitellaROR ------------GGAGAC---------------AGTTTGACCATCAAGATATCTGACTTT

ChickenTrkA ------------GGCCAC---------------GACCTGGTGGTGAAGATTGGGGACTTC

ZebrafishTrkA ------------GGAGAA---------------GGGCTGGTAGTGAAGATCGGGGACTTT

ZebrafishTrkC2 ------------GGGAAT---------------GGGCTGCTTGTGAAAATAGGAGACTTT

DaphniaNRK CCGCCCAGCAGCGGCGGA---------------GGCGTCACCGTCAAGATCGCCGACTTT

PeaAphidROR ------------GCTGAA---------------AATCTTACAGTGAAAATATCAGATTTT

PeaAphidNRK ------------GATGAT---------------ACAATGACAGTAAAAATAGCAGATTTT

LottiaNRK ------------GGTGAA---------------GATTTAAAAGTAAAAATATCCGACTTC

AplysiaTrkL ------------GGCACAGCGCCACCTGGTGCCGGACTTCTGGTGAAGATTTCCGACTTT

SeaUrchinTrk ------------GGGGAT---------------CGACTCATCGTCAAGATTGCAGACTTT

CapitellaTrk ------------GGTGAA---------------AGACTAACTGTGAAAATCGGAGACTTT

AplysiaROR ------------GCTGAC---------------GGGCTAACTGTGAAGATCTCCGACTTT

ChickenROR ------------GGRGWA---------------CAGCTTCATGTGRAAATTTCAGACCTT

CapitellaNRK ------------GGTGAG---------------CACATGGATGTCAAGATCTCAGACTTT

ChickenTrkC ------------GGAGCC---------------AACCTGCTGGTGAAGATTGGAGACTTT

ZebrafishTrkB1 ------------GGAGAA---------------AATCTCTTGGTGAAGATTGGAGACTTT

HoneyBeeNRK ------------AATGAT---------------CAAATGATTGTAAAGATAGCAGATTTC

HumanROR ------------GGAGAG---------------CAACTTCATGTAAAGATTTCAGACTTG

ZebrafishTrkB2 ------------GGGGAA---------------AACCTGCTTGTAAAGATAGGAGACTTT

JewelWaspTrkL ------------GGCTCC---------------GGACTCGTCGTTAAAATCGCCGACTTC

LottiaTrk ------------GGTGAG---------------AAGTTGTTAGTGAAGATCGGAGATTTT

DaphniaTrkL ------------GGTGAACGACCCCGAGGCGGCGGTCTGGCCGTCAAAATCTCCGATTTC

LymneaTrk ------------GGCTGT---------------GATCTTGTGGTCAAACTCGGTGACTTT

DrosophilaDTrk ------------TCCAGT---------------GAGTTTATAGTGAAGGTATCCTATCCG

AmphioxusTrk ------------GGGGAC---------------AAACTCGTGGTGAAGATCGGAGACTTC

DaphniaROR ------------AGTGAC---------------AACCTGATTGTGAAAATATCCGATTTT

ZebrafishTrkC1 ------------GGGAAT---------------GGGCTGCTTGTGAAAATAGGAGACTTT

HumanTrkC ------------GGAGCG---------------AATCTGCTAGTGAAGATTGGGGACTTC

HoneyBeeTrkL ------------GGTTAT---------------GATTTCATTGTTAAAATAGCAGATTTT

HumanTrkA ------------GGCCAG---------------GGACTGGTGGTCAAGATTGGTGATTTT

LottiaROR ------------GGTGAT---------------AATCTTATTATTAAGATATCTGATTTT

HumanTrkB ------------GGGGAG---------------AACTTGCTGGTGAAAATCGGGGACTTT

DrosophilaROR GGACTATCCAGAGACATTTACAGCTCAGATTATTATCGA---------------------

SeaUrchinROR GGTCTGGCAAGGGATATTTATTCAGGGGATTACTATAGG---------------------

ChickenTrkB GGGATGTCTAGAGATGTGTACAGCACAGACTACTACAGG---------------------

HoneyBeeROR GGTTTATCTCGTGATATATACAGTAGTGATTACTACAGA---------------------

LottiaTrkL GGAATGTCCCGGGATATATACACCTGTGATTATTATAAG---------------------

PeaAphidTrkL GGAATGAGCCGGGATATTTACACCTGTGACTATTACAAA---------------------

DaphniaTrk GGTATGTCCAGGGATATTTACAGCTCCGACTACTATCGGGTAAGTGCAGGATCACACCCT

JewelwaspNRK GGACTCTCGCAGAAGATCTACCTTCAAGACTACTACAAA---------------------

AmphioxusROR GGCCTTTCCCGCGATATCTACTCCTCGGATTACTACCGT---------------------

DrosophilaNRK GGGCTCTCGCACAAGATCTATTTGCAGGACTATTACAAA---------------------

JewelWaspROR GGTCTTTCTCGGGATATCTATAGCAGCGATTACTATAGA---------------------

CapitellaROR GGACTGTCCCGGGACGTCTACTCGTCAGACTACTACCGT---------------------

ChickenTrkA GGCATGTCCCGTGATATCTACAGCACCGACTACTACCGG---------------------

ZebrafishTrkA GGGATGTCAAGAGACATTTACAGTACTGACTACTACAGG---------------------

ZebrafishTrkC2 GGGATGTCGAGAGACATTTACAGCACTGACTACTACCGG---------------------

DaphniaNRK GGCCTGTCGCAGCGCGTCCACTGGCAGCAGTACTACTAC---------------------

PeaAphidROR GGTCTTTCAAGGGATATTTATTCATCTGATTATTACAGA---------------------

PeaAphidNRK GGGCTTTCGCAGAAAATGTATTTACAAGACTATTACAAA---------------------

LottiaNRK GGTCTAGCAAGGACAGTTCACAGCATAGAATATTATCGT---------------------

AplysiaTrkL GGCATGTCTAGGGATATCTACACTAACGACTACTACAAG---------------------

SeaUrchinTrk GGCATGTCAAGAGATGTTTATAGCACAGATTACTACAGG---------------------

CapitellaTrk GGAATGTCCCGCGATGTCTACAGCACTGATTACTACAGG---------------------

AplysiaROR GGTTTGTCCAGAGATGTCTACTCTTCTGATTACTACAGA---------------------

ChickenROR GGACTCTCAAGRGAAATCTACTCAGCAGATTATTACAGA---------------------

CapitellaNRK GGCCTGACGCGGTACGTCGTGCCGGGGGAGTGCTTCCAG---------------------

ChickenTrkC GGGATGTCAAGAGATGTCTACAGCACTGATTACTACAGGGAAGGGCCGCGTCCGAAGGGC

ZebrafishTrkB1 GGGATGTCTCGAGATGTCTACAGTACGGATTACTATCGG---------------------

HoneyBeeNRK GGTTTATCGCAAAAAATCTATTTACAAGATTACTATAAA---------------------

HumanROR GGGCTTTCCAGAGAAATTTACTCCGCTGATTACTACAGG---------------------

ZebrafishTrkB2 GGCATGTCCAGAGATGTGTACAGCACCGACTACTATAGG---------------------

JewelWaspTrkL GGCATGTCGCGCGACGTCTACACCTGCGACTACTACAAG---------------------

LottiaTrk GGAATGTCGAGAGATATTTATAGTACAGATTATTATCGG---------------------

DaphniaTrkL GGAATGAGCCGTGACGTTTACACTTGCGATTATTACAAG---------------------

LymneaTrk GGCATGTCCAGAGATGTGTACACTACGGACTACTACAGG---------------------

DrosophilaDTrk GCTCTCTGCAAGGACAAGTATAGCCGCGAGTACCACAAA---------------------

AmphioxusTrk GGGATGTCTCGGGACATCTACAGCACCGACTACTATAGA---------------------

DaphniaROR GGTCTGTCTCGAGACATTTACTCGTCTGATTACTACCGT---------------------

ZebrafishTrkC1 GGGATGTCGAGAGACATTTACAGCACTGACTACTACCGG---------------------

HumanTrkC GGCATGTCCAGAGATGTCTACAGCACGGATTATTACAGG---------------------

HoneyBeeTrkL GGGATGTCACGAGATGTTTATACTTGCGATTACTATAAG---------------------

HumanTrkA GGCATGAGCAGGGATATCTACAGCACCGACTATTACCGT---------------------

LottiaROR GGGTTATCACGAGATATCTACTCTTCTGATTATTATAGA---------------------

HumanTrkB GGGATGTCCCGGGACGTGTACAGCACTGACTACTACAGG---------------------

DrosophilaROR ---------------------------------------------------------GTT

SeaUrchinROR ---------------------------------------------------------ATG

ChickenTrkB ---------------------------------------------------------GTT

HoneyBeeROR ---------------------------------------------------------GTT

LottiaTrkL ---------------------------------------------------------ATA

PeaAphidTrkL ---------------------------------------------------------GTC

DaphniaTrk GAAGAAAGAATGAGACAAATTACAGCTTGTCAAAGTGAAAAATTGATATTTTACAAGGTT

JewelwaspNRK ---------------------------------------------------------GGG

AmphioxusROR ---------------------------------------------------------GTG

DrosophilaNRK ---------------------------------------------------------GGC

JewelWaspROR ---------------------------------------------------------GTA

CapitellaROR ---------------------------------------------------------GTG

ChickenTrkA ---------------------------------------------------------GTG

ZebrafishTrkA ---------------------------------------------------------GTC

ZebrafishTrkC2 ---------------------------------------------------------GTG

DaphniaNRK ------------------------------------------------------ACGGGG

PeaAphidROR ---------------------------------------------------------GTA

PeaAphidNRK ---------------------------------------------------------GGT

LottiaNRK ---------------------------------------------------------GGC

AplysiaTrkL ---------------------------------------------------------ATC

SeaUrchinTrk ---------------------------------------------------------GTT

CapitellaTrk ---------------------------------------------------------GTT

AplysiaROR ---------------------------------------------------------GTG

ChickenROR ---------------------------------------------------------GTT

CapitellaNRK ---------------------------------------------------------GGG

ChickenTrkC CAGCTCAGCACA---GCGTGGCAGCGACACAGACTGGCACCGCCGGCAGCTGCAACAGTT

ZebrafishTrkB1 ---------------------------------------------------------GTT

HoneyBeeNRK ---------------------------------------------------------GGC

HumanROR ---------------------------------------------------------GTC

ZebrafishTrkB2 ---------------GTGTTTGACCCGTACGGCACCTCAGAACATCTTTGGTGCGATGTG

JewelWaspTrkL ---------------------------------------------------------ATC

LottiaTrk ---------------------------------------------------------GTC

DaphniaTrkL ---------------------------------------------------------ATT

LymneaTrk ---------------------------------------------------------GTT

DrosophilaDTrk ------------------------------------------------------------

AmphioxusTrk ---------------------------------------------------------GTT

DaphniaROR ---------------------------------------------------------GTC

ZebrafishTrkC1 ---------------------------------------------------------GTG

HumanTrkC ---------------CTCTTTAATCCATCTGGAAATGATTTTTGTATATGGTGTGAGGTG

HoneyBeeTrkL ---------------------------------------------------------ATA

HumanTrkA ---------------------------------------------------------GTG

LottiaROR ---------------------------------------------------------GTC

HumanTrkB ---------------------------------------------------------GTC

DrosophilaROR CAGTCAAAGTCGCTATTGCCTGTAAGGTGGATGCCCTCGGAATCGATATTGTATGGAAAG

SeaUrchinROR CCCTCGCAGGCCGTTCTGCCAATCCGCTGGATGTCTCCGGAAGCTATCATGTTCGGTCGC

ChickenTrkB GGGGGTCACACCATGCTGCCCATTCGCTGGATGCCTCCAGAAAGTATCATGTATAGGAAG

HoneyBeeROR CAATCTAAGAGTTTATTACCCGTTCGATGGATGCCACCAGAGTCAATTCTTTACGGCAAA

LottiaTrkL GGAGGATCTCGGATGCTGCCAATACGGTGGATGTCTCCGGAAAGTGTCAAATATGGAAAA

PeaAphidTrkL GGAGGATCCAGATTGCTTCCCGTAAGGTGGATGTCGCCAGAGAGCGTCGTATATGGGAAA

DaphniaTrk GGTGGACACACTTTACTTCCAGTCCGGTGGATGCCTCCAGAGAGTGTTATGTACCGCAAA

JewelwaspNRK GACGACCAGGACGCTATTCCCGTGAGATGGATGCCACTGGAGAGTATTTTATACAACAAA

AmphioxusROR CAGTCCAAGTCGCTCCTGCCGGTTCGCTGGATGCCGCCGGAGGCGATCATGTACGGGAAG

DrosophilaNRK GATGAGAACGACTTCATCCCGATCCGCTGGATGCCACTTGAGAGCATACTGTACAACAAG

JewelWaspROR CAATCAAAAAGTTTATTGCCTGTAAGATGGATGCCACCAGAATCGATCTTATACGGAAAA

CapitellaROR CAGAGCAAGTCTCTGATGCCTGTGCGCTGGATGCCTCCAGAGTCTATCCTGTACGGAAAA

ChickenTrkA GGCGGTCGGACCATGCTGCCCATCCGCTGGATGCCCCCTGAGAGCATCCTGTACCGCAAG

ZebrafishTrkA GGAGGCAGGACGATGCTGCCCATTCGCTGGATGCCACCGGAAAGCATTATGTACAGGAAG

ZebrafishTrkC2 GGAGGACACACTATGCTGCCCATTCGCTGGATGCCACCAGAAAGCATCATGTACAGGAAG

DaphniaNRK ACGGACAACGATGCCATACCCATCCGCTGGATGCCGCTGGAGAGCATCATCTTCAACCGC

PeaAphidROR CAATCAAAATCATTGTTACCTGTAAGATGGATGCCACCTGAATCTATTCTGTACGGTAAA

PeaAphidNRK GATGAACATGATGCCATACCTGTTCGATGGATGCCTTTAGAGAGCATTCTTTACAATAAA

LottiaNRK AGTGAAAATGATGCAATACCTATCAGGTGGATGCCTCCAGAGGCAATCTTGTACAATAAA

AplysiaTrkL GGCGGGTCTCGAATGCTTCCAATCCGCTGGATGTCACCGGAAGCCATCAAGTATGGCCGC

SeaUrchinTrk GGGGGACACACGATGCTACCCGTCCGATGGATGCCTCCAGAGAGCATCATCTATAGAACC

CapitellaTrk GGAGGTCAGACCATGCTACCTGTGAGGTGGATGCCTCCGGAGAGCCTCCTGTATCGAACA

AplysiaROR CAGAGCAAGTCTTTGCTCCCCGTCAGATGGATGCCCCCGGAAGCGATCTTGTACGGAAAG

ChickenROR CAAAACAAATCTCTTCTGCCGATTCGCTGGATGCCACCGGAGGCGATTATGTATGGCAAG

CapitellaNRK AGCGAAAACGACGCCATTCCTATTCGCTGGATGCCACTCGAGGCAATCCTCTACAACAAG

ChickenTrkC GGAGGACACACCATGCTGCCTATCCGCTGGATGCCTCCGGAGAGCATCATGTACAGGAAG

ZebrafishTrkB1 GGAGGTCACACCATGCTGCCAATCCGCTGGATGCCACCGGAGAGTATCATGTATCGGAAG

HoneyBeeNRK GATGAACAAGATGCTATTCCAGTTAGGTGGATGCCTTTAGAAAGTATATTATATAATAAG

HumanROR CAGAGTAAGTCCTTGCTGCCCATTCGCTGGATGCCCCCTGAAGCCATCATGTATGGCAAA

ZebrafishTrkB2 GGTGGTCACACCATGCTGCCCATCCGATGGATGCCCCCAGAGAGCATTATGTACAGAAGA

JewelWaspTrkL GGCGGCTCGCGGTTATTGCCGGTGCGTTGGATGTCTCCGGAGAGCGTGATGTACGGGAGA

LottiaTrk GGTGGAACCGCCATGTTGCCTATCAGATGGATGCCTCCAGAAAGCATGTTGTATCGAACA

DaphniaTrkL GGAGGTTCTCGGATGCTGCCGGTGCGATGGATGGCTCCCGAATCGATCCTCTACGGTAAA

LymneaTrk GAAGGCACGGCCATGCTGCCTGTAAGATGGATGCCGCCGGAGAGCATCATCTACAGGACG

DrosophilaDTrk CACCGCAACACGCTGCTCCCGATCCGCTGGCTGGCGCCCGAGTGCATCCAGGAAGACGAG

AmphioxusTrk GGGGGACACACGATGCTGCCTGTACGGTGGATGCCACCAGAAAGCGTGCTCTACCGGAAA

DaphniaROR CAGGGCAAATCAATGTTGCCTGTTCGTTGGATGCCACCAGAAGCTATTCTATACGGAAAA

ZebrafishTrkC1 GGAGGACACACTATGCTGCCCATTCGCTGGATGCCACCAGAAAGCATCATGTACAGGAAG

HumanTrkC GGAGGACACACCATGCTCCCCATTCGCTGGATGCCTCCTGAAAGCATCATGTACCGGAAG

HoneyBeeTrkL AAGGGCTCTAGACTTTTACCAATCCGTTGGATGTCACCGGAAAGTGTAATGTACGGGCGA

HumanTrkA GGAGGCCGCACCATGCTGCCCATTCGCTGGATGCCGCCCGAGAGCATCCTGTACCGTAAG

LottiaROR CAGAGTAAATCTCTACTCCCTGTTCGTTGGATGCCTCCAGAGTCAATAATGTATGGTAAA

HumanTrkB GGTGGCCACACAATGCTGCCCATTCGCTGGATGCCTCCAGAGAGCATCATGTACAGGAAA

DrosophilaROR TTTACGACCGAGAGCGATGTTTGGTCCTTTGGAGTCGTTCTTTGGGAAATATACAGCTAT

SeaUrchinROR TTCACGGTGGAGAGCGATATCTGGTCCTTTGGTGTGGTGTTATGGGAGATTTACTCGTAC

ChickenTrkB TTCACCACTGAAAGTGATGTCTGGAGCCTGGGAGTTGTATTATGGGAAATCTTTACCTAT

HoneyBeeROR TTTACAACAGAGTCCGATGTATGGAGTTTCGGAGTCGTATTGTGGGAAATTTATAGTTAC

LottiaTrkL TTTACAATAGAGTCAGATGTCTGGTCATACGGTGTTGTATTATGGGAAATATTCAGTGCT

PeaAphidTrkL TTTACGCTAGAATCTGACGTGTGGTCATTCGGTGTCGTGTTGTGGGAAATTTATTCGCTG

DaphniaTrk TTCACGTCCGAGTCGGACGTCTGGTCATTTGGAGTGGTGCTTTGGGAAATCTTCTCCTTT

JewelwaspNRK TACACAGTGGAGTCAGATGTCTGGGCTTTTGCTGTATGCTTATGGGAAATATTTAGCTTC

AmphioxusROR TTTTCCACGGACAGCGATGTTTGGTCGTTCGGCGTGGTTCTGTGGGAGATCTTCAGCTAC

DrosophilaNRK TTCTCGCTTGAGTCGGATGTGTGGGCATACGGCATCTGTCTGTGGGAGGTCTTCTCCTTC

JewelWaspROR TTCACAACAGAATCTGATGTTTGGAGTTTTGGTGTTGTTTTATGGGAGATTTACAGTTAT

CapitellaROR TTTACAATGGAAAGTGACGCCTGGTCATTTGGAGTCGTGTTGTGGGAGATCTATTCATTT

ChickenTrkA TTCACCACTGAGAGTGATATCTGGAGCTTCGGCGTGGTGCTTTGGGAGATCTTCACCTAT

ZebrafishTrkA TTCACCACAGAGAGTGACATCTGGAGCTTCGGAGTCGTTTTGTGGGAAATCTTCACCTAT

ZebrafishTrkC2 TTCACGACGGAGAGTGACGTGTGGAGTTTTGGGGTCATCCTCTGGGAAATATTTACCTAC

DaphniaNRK TACACCACGTCGTCGGATGTCTGGGCCTTTGGCGTCTGCCTCTGGGAGATCTTCTCCTAC

PeaAphidROR TTTACAACTGAATCTGATGTTTGGAGTTTTGGTGTTGTATTATGGGAAGTTTACAGCTAT

PeaAphidNRK TATACAGTTGAATCAGATGTCTGGGCATTTGGTGTGTGTCTTTGGGAAATTTTTTCTTTT

LottiaNRK TTTTCAGTTAAATCAGACGTATGGTCATTTGGTGTAGTTCTGTGGGAAATTTTTTCATTT

AplysiaTrkL TTCACATGTGAGAGTGATGTGTGGGCCTATGGAGTTGTCTTATGGGAGATTTTCAGTTAC

SeaUrchinTrk TACTCCGTGGAAAGTGATGTCTGGAGCTATGGGGTGGTCCTTTGGGAGATTTTCGAGTAT

CapitellaTrk TTCACGGTTGAGTCTGATGTATGGAGCTTTGGAGTGGTTTTATGGGAGATTTTTACGTAC

AplysiaROR TTCACCACAGACAGTGATGTGTGGGCTTTTGGCGTTGTCCTCTGGGAGGTCTTCAGCTAT

ChickenROR TTCTCTTCTGATTCAGATATTTGGTCATTCGGAGTGGTTTTATGGGAGATATTCAGCTTT

CapitellaNRK TTCACCAGCGAATCAGACGTGTGGTCTTTTGGAGTTCTTCTGTGGGAGATCTTCTCCTTC

ChickenTrkC TTCACAACGGAGAGTGACGTCTGGAGCTTCGGGGTGATCCTCTGGGAGATCTTCACCTAT

ZebrafishTrkB1 TTCACAACAGAGAGTGATGTCTGGAGTCTGGGAGTGGTGCTGTGGGAGATCTTTACCTAT

HoneyBeeNRK TATACCGTTGAATCTGATGTATGGGCGTTCGCGGTATGCTTGTGGGAAATATTTAGTTTT

HumanROR TTCTCTTCTGATTCAGATATCTGGTCCTTTGGGGTTGTCTTGTGGGAGATTTTCAGTTTT

ZebrafishTrkB2 TTCACCACAGAGAGTGACGTGTGGAGTTTGGGTGTAGTACTCTGGGAAATCTTCACCTAT

JewelWaspTrkL TTCACCCTGGAAAGCGACGTTTGGAGCTTCGGAGTCGTTCTCTGGGAGGTCTACTCCTTC

LottiaTrk TTTACCATAGAATCCGATGTATGGAGTTTTGGTGTTGTATTATGGGAAATATTCACATAT

DaphniaTrkL TTCACGCTAGAATCGGATGTCTGGAGTTTCGGCGTCGTCCTGTGGGAGGTCTTTGCCCTA

LymneaTrk TTCACCACTGAATCAGACGTCTGGAGCTTTGGCGTAACGCTCTGGGAGGTCTTCACGTAC

DrosophilaDTrk TACACCACCAAGAGCGACATCTTTGCCTACGGTGTGGTGGTGTGGGAGCTCTTCAACCAG

AmphioxusTrk TTCACCATCGAAAGTGACATCTGGAGTTTCGGCGTCGTGCTATGGGAAATCTTTACGTTT

DaphniaROR TTCACAATTGAATCAGATGTTTGGAGCTTCGGCGTTGTCCTCTGGGAAATCTACAGTTTT

ZebrafishTrkC1 TTCACGACGGAGAGTGACGTGTGGAGTTTTGGGGTCATCCTCTGGGAAATATTTACCTAC

HumanTrkC TTCACTACAGAGAGTGATGTATGGAGCTTCGGGGTGATCCTCTGGGAGATCTTCACCTAT

HoneyBeeTrkL TTCACATTGGAAAGTGACGTATGGAGTTTTGGCGTTGTTCTATGGGAAGTCTATTCCTTT

HumanTrkA TTCACCACCGAGAGCGACGTGTGGAGCTTCGGCGTGGTGCTCTGGGAGATCTTCACCTAC

LottiaROR TTCACCACCGATAGTGATGTATGGTCTTTTGGAGTTGTTCTGTGGGAAATATTCAGCTTT

HumanTrkB TTCACGACGGAAAGCGACGTCTGGAGCCTGGGGGTCGTGTTGTGGGAGATTTTCACCTAT

DrosophilaROR GGAATGCAG---CCATACTACGGTTTTAGCAATCAGGAA---------GTAATCAATCTC

SeaUrchinROR GGCTTGCAA---CCGTTCTATGGTTATAACAATAATGAG---------GTGATAGAGATG

ChickenTrkB GGCAAGCAG---CCATGGTACCAGCTCTCAAACAATGAG---------GTGATTGAGTGC

HoneyBeeROR GGTTTACAA---CCGTATTACGGATACAATAATCAAGAA---------GTAATAGACATG

LottiaTrkL GGACGACAG---CCATATTTTGGTCATTCGAATGAAGAG---------GTGATACAGTTT

PeaAphidTrkL GGAAAACAA---CCGTATTACGGTTATTCGAATGATGAG---------GTTTTAAAATTG

DaphniaTrk GGCAAACAG---CCTTGGTACGGCTATTCCAATCAAGAG---------GTCATACAACTA

JewelwaspNRK GCGCTTCAG---CCCTACTATGGTATGACGCACGAGGAA---------GTCGTTAAATAC

AmphioxusROR GGACTCCAG---CCGTACTACGGGTACAGCAACCAGGAG---------GTGATCGAGATG

DrosophilaNRK GCCTTGCAG---CCCTACTTTGGGTTAACCCACGAGGAG---------GTGATCAAATAC

JewelWaspROR GGCTTACAG---CCATATTACGGTTATAATAATCAAGAA---------GTAATTGACATG

CapitellaROR GGACTGCAG---CCGTACTACGGCTACTCCAATCAGGAG---------GTCATTGAGATG

ChickenTrkA GGGAAGCAG---CCCTGGTACCAACTCTCCAACACTGAG---------GCCATCGAGTGC

ZebrafishTrkA GGCAAACAA---CCCTGGTATCAGCTATCCAACAGTGAG---------GCCATTGAGTGC

ZebrafishTrkC2 GGCAAGCAG---CCATGGTTTCAGCTGGCAAATAATGAG---------GTAATCGAATGC

DaphniaNRK GCCCAGCAG---CCCTACCACGGAATGTCTCACGAGGAA---------GTCGTCCGCTAC

PeaAphidROR GGTTTACAA---CCATATTATGGATATAGCAATCAAGAA---------GTAATTGAAATG

PeaAphidNRK GCTCTTCAA---CCGTATTATGGCATGACACATGAAGAA---------GTCGTTAAATTC

LottiaNRK GCTATCCAG---CCTTACTATGGAATGACACATGAGGAA---------GTGGTGAAGTTT

AplysiaTrkL GGCAGACAA---CCATATTTTGGACATTCGAATGAGGAG---------GTTATTCACTTT

SeaUrchinTrk GGAAAGCAG---CCCTGGTTTGGCCTTTCCAATCACGAG---------GTGATCGAGTAC

CapitellaTrk GGTAAACAA---CCGTGGTACGAACTCTCCAACCACGAG---------GTGATTCAACAA

AplysiaROR GGACTGCAG---CCGTATTACGGTTTCTCCAATCAAGAG---------GTCATTGAGATG

ChickenROR GGACTTCAA---CCATATTACGGATTTAGTAATCAAGAA---------GTCATAGAGATG

CapitellaNRK GCCCTGCAG---CCCTATTACGGCATGGCACACGAAGAG---------GTCGTCAAGTAC

ChickenTrkC GGGAAGCAG---CCGTGGTTTCAGCTCTCAAACACAGAG---------GTCATTGAGTGC

ZebrafishTrkB1 GGAAAACAG---CCCTGGTACCAACTGTCAAATAATGAG---------GTGATCGAGTGC

HoneyBeeNRK GCGCTCCAG---CCTTATTATGGAATGACGCACGAAGAA---------GTTGTAAAATAT

HumanROR GGACTCCAG---CCATATTATGGATTCAGTAACCAGGAA---------GTGATTGAGATG

ZebrafishTrkB2 GGCAAGCAA---CCCTGGTATCAGCTCTCAAACAATGAG---------GTGATCGAGTGT

JewelWaspTrkL GGAAAGCAA---CCCTACTACGGACACAACAACGAGGAG---------GTCGTGAAGCTG

LottiaTrk GGTAAACAA---CCATGGTATGAACTATCAAACATGGAG---------GTTATTCAATAT

DaphniaTrkL GGGGTACAG---CCCTACTACGGCCATTCAAACGAACAG---------GTCGTCAAACTG

LymneaTrk GGCAAGCAG---CCGTGGTTTGAGTACTCCAATAGTGAG---------GTCATAGAACAC

DrosophilaDTrk GCCACCAAGCTGCCCCACGAGGAGCTGACCAACGAGCAG---------GTGGTGCAGCGT

AmphioxusTrk GGGAAACAG---CCGTGGTACGAGCTGGCCAATCACGAG---------GTGATCGAGTGC

DaphniaROR GCATTACAG---CCGTATTATGGATACAATAACCAAGAT---------GTCATCGACATG

ZebrafishTrkC1 GGCAAGCAG---CCATGGTTTCAGCTGGCAAATAATGAG---------GTAATCGAATGC

HumanTrkC GGAAAGCAG---CCATGGTTCCAACTCTCAAACACGGAG---------GTCATTGAGTGC

HoneyBeeTrkL GGCAAACAA---CCATATTACGGACATAATAATGAAGAAGTAAGTAAAGTAGTGAAGCTA

HumanTrkA GGCAAGCAG---CCCTGGTACCAGCTCTCCAACACGGAG---------GCAATCGACTGC

LottiaROR GGTCTTCAG---CCTTACTATGGTTTCTCTAATCAAGAA---------GTTATAGAAATG

HumanTrkB GGCAAACAG---CCCTGGTACCAGCTGTCAAACAATGAG---------GTGATAGAGTGT

DrosophilaROR ATCCGTTCACGGCAACTGCTCTCCGCT---CCGGAAAACTGTCCCACTGCTGTCTACTCG

SeaUrchinROR ATCCGAACCCGTCACATCCTGCCCAGC---CCTAGCGGTTGCCCTCAGTACATCTATGCA

ChickenTrkB ATCACTCAGGGCCGAGTCCTTCAGCGA---CCTCGCACGTGCCCCAAGGAAGTGTATGAC

HoneyBeeROR ATTCGATCACGGCAATTATTACCCTGC---CCCGAGGATTGTCCAACCATGATTTATAGT

LottiaTrkL CTAGATACTGGAATTCTACTACAGAGA---CCAAACGAGTGTCCATCTACCATATATCAC

PeaAphidTrkL ATTCACGATGGCGTGTTATTGGACATA---CCGATGAACTGCCCTCCTGTTATATGCGTG

DaphniaTrk GTCACAGGAGGCCAAGTACTTCCATGT---CCTCTTGCCACTCCTCCCGATGCGTATCAA

JewelwaspNRK ATCAAAGAGGGTAACGTTCTGCAGTGT---CCGGAAAACACTCCTCAATCTGTCTATGAT

AmphioxusROR ATCCGGTCGCGCCAGCTGCTGCCGTGC---CCCGACAACTGCCCGGCCAGGATGTACTCC

DrosophilaNRK ATCAAGGAGGGCAACGTACTCGGCTGT---CCGGACAACACGCCGCTCTCCGTCTACGCG

JewelWaspROR ATCCGCTCACGACAACTTCTTCCTTGC---CCAGAGGATTGCCCGACAATGATCTACAGT

CapitellaROR ATCCGTGCGCGGCAGATCCTGCCCTGC---CCTGAGGACTGTCCATCTCGTATTTATGCT

ChickenTrkA ATCACGCAGGGCCGGGAGCTGGAGCGG---CCCCGCACGTGTCCCTCCGAGGTGTATGAT

ZebrafishTrkA ATCACGCAGGGCCGAGAGCTGGAGCGG---CCTCGCACCTGCCCAAAAGAAGTGCACCTC

ZebrafishTrkC2 ATCACACAAGGACGGGTGCTAGAAAGG---CCACGGCTCTGTCCCAAGGAAGTGTACGAC

DaphniaNRK TTGCAGGCCGGCGGAATGCTCCAGCCG---CCGGCCCACGCGTCCTGTGCCATTTACGCC

PeaAphidROR ATTCGTTCGCGCCAACTACTTCCGTGC---CCTCAAGAATGTCCATCAAGAATGTACTCA

PeaAphidNRK ATCAAAGATGGCAACGTTATGAATTGT---CCTGAAAATACTCCAAAATTAGCATATGAG

LottiaNRK GTGAAAGAGGGTAAAGTGTTGGGTTGT---CCTGAAAACACTCCTCAACCTGTGTATGAA

AplysiaTrkL CTAGATCAAGGAATTCTCCTCCAGCGA---CCGGAAGACTGCCCCTCCACCGTCTATCAC

SeaUrchinTrk ATCCACAACGGGATATTACTGGACTGC---CCCAAAGGCTGCCCCAAGGAGGTCTACAAA

CapitellaTrk GTTCAGAGTGGTAAGCTGTTGGAATGC---CCGAGGCACTGCATGGAGACGCTATACAAA

AplysiaROR ATACGCTCCAGGCAGATTCTGGGCTGT---CCTGAAGAATGCCCGGCTCGTATTTACGGT

ChickenROR ATCAGGAAGCGACAGCTGCTGCCGTGC---TCTGAAGACTGTCCTCCTCGAATGTACAGC

CapitellaNRK GTTCGTGAGGGCAAAGTGCTCTCCTGT---CCGGATAACACTCCTCAGGAGATGTACGAC

ChickenTrkC ATTACCCAAGGCCGAGTTCTGGAAAGA---CCTCGAGTCTGCCCCAAGGAGGTATACGAC

ZebrafishTrkB1 ATCACCCAAGGTCGTGTACTGCAGCGA---CCCCGGACCTGTCCCAAGGAGGTGTATGAC

HoneyBeeNRK ATAAAGGAAGGCAATGTACTCCAATGT---CCCGAAAATACTCCACCGGCGATATATGAT

HumanROR GTGAGAAAACGGCAGCTCTTACCATGC---TCTGAAGACTGCCCACCCAGAATGTACAGC

ZebrafishTrkB2 ATCACACAGGGTCGGGTGTTACAGAGA---CCTCGTACTTGCCCCAAAGAAGTGTATGAT

JewelWaspTrkL ATCCTCCAAGGCATAATGCTGATCCCC---CCGGAAGACTGTCCCCCGTACGTCTGCCAG

LottiaTrk ATAAAGAACGGCCATATTTTAGAAAGA---CCACGTGATTGTCCAGAGGAAGTGTATAAA

DaphniaTrkL ATCCTACAAGGGATCCTCCTGACTCCT---CCTTCTTCAGCCCCGCCCCTCATTTGCCAG

LymneaTrk ATCAAAAATAGCCGGACCCTCAAGCGGCCTCCCAGGACCTGTACAGACGGCGTCTACAGA

DrosophilaDTrk TCCCAGGCGGGCTCCTTGGAGTGGTCAGTGGCCGAGGCGACGCCCGATAGCCTGCGAGAG

AmphioxusTrk ATCACCAGCGGCAGACTGTTGGGGTGC---CCGCGTGGCTGCCCACGGAACGTGCGGGCC

DaphniaROR GTGCGTTCTAGACAGCTACTCTCTTGC---CCATCAGAATGCCCGTCACGAATTTATTCC

ZebrafishTrkC1 ATCACACAAGGACGGGTGCTAGAAAGG---CCACGGCTCTGTCCCAAGGAAGTGTACGAC

HumanTrkC ATTACCCAAGGTCGTGTTTTGGAGCGG---CCCCGAGTCTGCCCCAAAGAGGTGTACGAT

HoneyBeeTrkL ATTTTTCAAGGCATAATGCTTATACCT---CCGGAAGGATGTCCACCATTTGTCTGCCAA

HumanTrkA ATCACGCAGGGACGTGAGTTGGAGCGG---CCACGTGCCTGCCCACCAGAGGTCTACGCC

LottiaROR ATCAGATCCCGTCAATTACTGCCTTGT---CCTGAAGAATGTCCAGCTAGAATCTATGGT

HumanTrkB ATCACTCAGGGCCGAGTCCTGCAGCGA---CCCCGCACGTGCCCCCAGGAGGTGTATGAG

DrosophilaROR CTAATGATCGAGTGCTGGCATGAGCAGTCAGTAAAACGTCCAACATTCACAGATATTTCG

SeaUrchinROR CTCATGACAGAATGCTGGAGTGAAATACCTGCTAGGAGACCTCCTTTCAAGGTCATCTCC

ChickenTrkB TTGATGCTGGGATGTTGGCAACGGGAACCTCATATGAGGCTCAACATCAAAGAAATCCAT

HoneyBeeROR CTAATGATAGAGTGCTGGCACGAGGTGGCTAATCGCAGACCGCAGTTCCCGGAGATTCAT

LottiaTrkL GTGATGTTAGGATGTTGGAAACAGGACCCAAAAGATCGCATGCCATTCCACAGAATCCAT

PeaAphidTrkL TTGATGAACGGATGTTGGAAAAGTGACCCCAAGGAACGCTTGCGGTTCATTGACATATAC

DaphniaTrk TTGATGTTGAATTGCTGGCAAACACAACCCAATCAGCGGAGCACGATGAAAGCGGTCAAT

JewelwaspNRK TTAATGAAACTTTGCTGGAATAGAAAGTCCTCAGACAGGCCTACTTTTAAATTCATCTAT

AmphioxusROR CTGATGCTGGAGTGCTGGAACGAGATCCCGGCGCGCAGACCGAGCTTCAACCAGATCCAC

DrosophilaNRK CTGATGCGTCGCTGCTGGAACCGCAAGCCCAGTGAGCGACCTGGCTTCGCCGAGATCAAC

JewelWaspROR CTGATGATAGAGTGTTGGCACGAGGTGGCCAATCGTCGGCCGCAGTTCCCGGAAATTCAC

CapitellaROR TTGATGGTCGAGTGTTGGCATGAGATGCCCATGCGTCGCCCTTCCTTCAAGGAAATCCAC

ChickenTrkA ATCATGCAGAGCTGCTGGCAGAGAGAGCCGCAGCAGCGC---AGCATCCAGGACATCCAC

ZebrafishTrkA CTGATGCAGGGCTGCTGGCAGAGAGAACCACAGCAAAGGTTGGTCATCAAGGACATCTAC

ZebrafishTrkC2 ATTATGCTGGGGTGCTGGCAGAGGGAACCACAACAGAGGTTAAACATCAAAGACATCCAG

DaphniaNRK GTCATGCGCTCCTGCTGGCACAGCTCAGCCGGAGAGCGGCCCAGCTTCGTCGACCTGCAC

PeaAphidROR CTAATGATGGAGTGTTGGCACGAGGCACCTGTACGACGACCCAATTTCACAGAAATCCAC

PeaAphidNRK CTTATGAAACAATGCTGGAGTCGAAAACCTGATTCACGACCTACTTTCCGTACAATTTAT

LottiaNRK CTGATGAATTTCTGTTGGATACGTAAACCAACTGAAAGACCAACATTCCGCAAATTACAC

AplysiaTrkL GTGATGCTTGGTTGCTGGAAGGGTGACCCCAGGCAGAGAATCGTCTTTGACCGTCTCCTC

SeaUrchinTrk ATCATGCTTGGATCCTGGCAGCGGCAACCAACACAGCGCATGCTCATTAAAGACTTGCAC

CapitellaTrk CTGATGCTTGGTTGCTGGAGAAGACAGCCTTCGGATCGCTTCACCATGAAAGAAATAAAC

AplysiaROR CTGATGGTGGAGTGTTGGCACGAGATGCCCGCCAGGCGTCCTCCTTTCCGGGAAATTCAC

ChickenROR CTGATGACGGAGTGCTGGCACGACTTGCCCTCTCGGAGGCCGCGATTTAAAGAAATCCAC

CapitellaNRK ATGATGCGCCTGTGTTGGAGTAAGCGTCCCGCCATGCGCCCTCCCTTCAGAGCCCTCCAC

ChickenTrkC ATCATGTTGGGCTGCTGGCAGAGAGAACCTCAGCAACGGCTCAACATCAAGGAGATCTAC

ZebrafishTrkB1 TTGATGCTGGGATGTTGGCAGAGAGAGCCGCACACCCGACTCAATATTAAAGAGATCCAT

HoneyBeeNRK TTGATGAAACTTTGTTGGAACAGGAGACCATCAGATAGACCTACTTTCAGAACAATTTAT

HumanROR CTCATGACAGAGTGCTGGAATGAGATTCCTTCTAGGAGACCAAGATTTAAAGATATTCAC

ZebrafishTrkB2 CTGATGCTGGGCTGCTGGCAGAGGGAGCCATACATGAGACTCAACATCAAAGAAATCCAC

JewelWaspTrkL ATAATGAGGGACTGCTGGAAGACGGAGCCCCGAGATCGTCTGCGCTTCGTCGACATCCTG

LottiaTrk GTGATGTTATGTTGTTGGAAAACTCAGCCACACGATCGAATGCCAATGAAAGAGATACAC

DaphniaTrkL TTGCTAAACGGTTGCTGGAAAACACAGCCGGGGGATCGGCTAACGTTCGCCGAAATCCAT

LymneaTrk GTAATGCAGGGGTGTTGGAAGCCCAACCCACAAGACAGACTGACTATGAAAGATATCGCG

DrosophilaDTrk ATATTGCTGTCCTGTTGGGTGTCCAATCCGAAGGAGCGACCTTCGTTCAGCCAACTGGGA

AmphioxusTrk CTCATGCTCGGCTGCTGGAAGAAAACCCCCGCCCAGCGCACCAACATCCAGGACATCCAC

DaphniaROR TTGATGATCGAGTGCTGGTCTGAAGTTCCTCTGAGGAGGCCCACATTCACAGAGGTTCAC

ZebrafishTrkC1 ATTATGCTGGGGTGCTGGCAGAGGGAACCACAACAGAGGTTAAACATCAAAGACATCCAG

HumanTrkC GTCATGCTGGGGTGCTGGCAGAGGGAACCACAGCAGCGGTTGAACATCAAGGAGATCTAC

HoneyBeeTrkL ATAATGCGAGAATGTTGGAAAACTGATCCTAAGGATAGAATTAAATTTCCGGAGATATTA

HumanTrkA ATCATGCGGGGCTGCTGGCAGCGGGAGCCCCAGCAACGCCACAGCATCAAGGATGTGCAC

LottiaROR TTAATGGTAGAGTGTTGGCATGAGATGCCTAATAGAAGACCACCATTCAGAGAGATTCAT

HumanTrkB CTGATGCTGGGGTGCTGGCAGCGAGAGCCCCACATGAGGAAGAACATCAAGGGCATCCAT

DrosophilaROR AACCGTCTC

SeaUrchinROR ACACGCCTC

ChickenTrkB TCCCTCCTT

HoneyBeeROR CATCGTCTG

LottiaTrkL AACTATCTT

PeaAphidTrkL GAACGTTTG

DaphniaTrk GAACGTTTA

JewelwaspNRK GAAACACTT

AmphioxusROR ACCCGGTTA

DrosophilaNRK CACTGCATC

JewelWaspROR CACCGGCTA

CapitellaROR CAGCGTCTC

ChickenTrkA AGCCGCCTG

ZebrafishTrkA AGTCGCCTC

ZebrafishTrkC2 AAAATTCTT

DaphniaNRK GACGAGCTT

PeaAphidROR TCTCGACTT

PeaAphidNRK CAAACTTTA

LottiaNRK AAAGCAATT

AplysiaTrkL AAGTACCTG

SeaUrchinTrk GACGCAATC

CapitellaTrk GCGAAATTG

AplysiaROR ACTCGACTT

ChickenROR GCCAGGCTG

CapitellaNRK GCCTCGCTC

ChickenTrkC AAGATCCTC

ZebrafishTrkB1 AACCTACTT

HoneyBeeNRK AATACTCTT

HumanROR GTCCGGCTT

ZebrafishTrkB2 AATCTGCTC

JewelWaspTrkL GAGAAGCTC

LottiaTrk AGACATTTA

DaphniaTrkL TCCAAGTTA

LymneaTrk GAGCTCCTC

DrosophilaDTrk GCTGCTCTC

AmphioxusTrk AAGAAGCTC

DaphniaROR AACAGATTG

ZebrafishTrkC1 AAAATTCTT

HumanTrkC AAAATCCTC

HoneyBeeTrkL GAAAGACTG

HumanTrkA GCCCGGCTG

LottiaROR GCCCGATTG

HumanTrkB ACCCTCCTT
